# The Synthetic Elicitor DPMP (2,4-dichloro-6-{(E)-[(3-methoxyphenyl)imino]methyl}phenol) Triggers Strong Immunity in *Arabidopsis thaliana* and Tomato

**DOI:** 10.1038/srep29554

**Published:** 2016-07-14

**Authors:** Yasemin Bektas, Melinda Rodriguez-Salus, Mercedes Schroeder, Adilene Gomez, Isgouhi Kaloshian, Thomas Eulgem

**Affiliations:** 1 Center for Plant Cell Biology, Institute for Integrative Genome Biology, University of California at Riverside, CA 92521, USA; 2 Department of Botany and Plant Sciences, University of California at Riverside, CA 92521, USA; 3 ChemGen Intergrative Graduate Education and Research Traineeship program, program, University of California at Riverside, CA 92521, USA; 4 Department of Nematology, University of California at Riverside, CA 92521, USA

## Abstract

Synthetic elicitors are drug-like compounds that are structurally distinct from natural defense elicitors. They can protect plants from diseases by activating host immune responses and can serve as tools for the dissection of the plant immune system as well as leads for the development of environmentally-safe pesticide alternatives. By high-throughput screening, we previously identified 114 synthetic elicitors that activate expression of the pathogen-responsive *CaBP22*^*−333*^*::GUS* reporter gene in *Arabidopsis thaliana* (Arabidopsis), 33 of which are [(phenylimino)methyl]phenol (PMP) derivatives or PMP-related compounds. Here we report on the characterization of one of these compounds, 2,4-dichloro-6-{(E)-[(3-methoxyphenyl)imino]methyl}phenol (DPMP). DPMP strongly triggers disease resistance of Arabidopsis against bacterial and oomycete pathogens. By mRNA-seq analysis we found transcriptional profiles triggered by DPMP to resemble typical defense-related responses.

Plant innate immunity is based on a complex set of integrated defense mechanisms protecting against microbial diseases^1^,^2^,^3^. Plants can recognize microbe-associated molecular patterns (MAMPs), which are highly conserved molecular structures of microbes, via pattern recognition receptors (PRRs) on the surfaces of plant cells. These interactions activate pattern-triggered immunity (PTI)^4^,^5^,^6^,^7^,^8^,^9^,^10^,^11^. To attenuate or block PTI, pathogens often secrete into plant cells effector molecules that enable them to use a given plant species as a host, resulting in compatible interactions, a condition also termed effector-triggered susceptibility (ETS). During this type of interaction plants can still exhibit a weakened immune response, called basal defense, which limits the spread of virulent pathogens, but is insufficient for preventing disease^11^,^12^. As a countermeasure to ETS, plants often can recognize the presence or activity of effector proteins by highly specific plant resistance (R) proteins and induce effector-triggered immunity (ETI). This leads to incompatible interactions leaving the pathogen avirulent and the plant resistant^5^,^13^.

PTI, basal defense and ETI are controlled by a common set of defense signals including reactive oxygen intermediates (ROIs), Ca^2+^, salicylic acid (SA), ethylene (ET) and jasmonic acid (JA)^14^. The massive release of ROIs at pathogen infection sites is one of the earliest observable features of a plant’s defense program. Induced changes of ion fluxes typically precede this oxidative burst^15^. The oxidative burst conditions a programmed form of localized cell death at infection sites, termed hypersensitive response (HR). HR can limit invasion of biotrophic pathogens, as these require host tissues to remain intact^16^. These early responses are coordinated by various components of SA-dependent signaling mechanisms^14^. In addition, crosstalk between the SA, JA and ET hormone pathways are important for the fine-tuning of plant defense responses^17^.

Inducible immune responses are tightly associated with extensive transcriptional- and metabolic–reprogramming controlled by a complex regulatory network^1^,^2^,^3^. This network can be subdivided into various defined sectors that can interact with each other^2^,^3^. For example, distinct defense signaling sectors dependent on early MAMP-activated MAP kinases or the defense hormones SA or JA, have been described for *Arabidopsis thaliana* (Arabidopsis).

Synthetic elicitors (aka plant activators) are small molecules, which activate plant immune responses and can protect plants from diseases without the need to be directly toxic to pathogens. One of the first classes of synthetic elicitors, low molecular weight polyacrylic acid derivatives, were identified in 1974 and were shown to activate resistance of tobacco against viruses^18^,^19^. Subsequently, a large number of synthetic compounds were found to exhibit defense elicitor activity in plants. Most of them can be broadly classified as SA analogs, imprimatins, sulfonamides, adipic acid derivatives or jasmonic acid analogs^20^. While some of them were used in basic research, others have been effectively used in crop protection.

The frequently used SA analogs 2,6-dichloro-isonicotinic acid (INA) and benzo (1,2,3) thiadiazole-7-carbothioic acid *S*-methyl ester (BTH) were discovered by Ciba-Geigy (now Syngenta) in early 1990s^21^,^22^,^23^. Interactions of these two compounds with the plant defense system have been well characterized^24^,^25^,^26^,^27^,^28^,^29^. Both BTH and INA trigger defense-associated effects similar to SA, but are less phytotoxic and more efficient than this natural defense hormone^20^,^22^,^26^,^27^. In addition to BTH, which has been marketed by Syngenta under the name Bion or Actigard, other SA analogs (Probenazole, Tiadinil, Isotianil) have been successfully used in agriculture to protect plants against disease^20^.

Initiating a chemical genomics (chemetics)–related study on the plant immune system, we identified by high throughput screening 114 synthetic elicitors. One of them, 3-5-dichloroanthranilic acid (DCA) induces in Arabidopsis fast and transient defense responses against the pathogenic oomycete *Hyaloperonospora arabidopsidis (Hpa*) and the bacterial pathogen *Pseudomonas syringae*^30^. In contrast to INA and BTH, DCA acts largely independent from NPR1, a central regulator of SA-responsive defense reactions in Arabidopsis. Another synthetic elicitor we recently characterized is 2-(5-bromo-2-hydroxy-phenyl)-thiazolidine-4- carboxylic acid (BHTC)^31^. Similar to DCA, it induces plant defense quickly and transiently, but its mode-of-action is different from that of DCA, since it strongly depends on NPR1.

Here, we report on another new synthetic elicitor identified by our previous high throughput screen, 2,4-dichloro-6-{(E)-[(3-methoxyphenyl)imino]methyl}phenol (DPMP), which is a member of the structural class of phenyl-imino-methyl-phenol derivatives (PMPs). DPMP is the most potent synthetic elicitor that we have identified so far, since it induces plant defense responses at very low concentrations. Its activity is distinct from that of DCA and similar to BHTC, since its ability to induce immunity against *Hpa* is completely blocked in *npr1* mutant plants. An mRNA-seq analysis of DPMP-induced transcriptional responses has further revealed that, although DPMP acts as a partial agonist of SA and mimics some SA functions, it also induces expression of 388 genes that are uniquely targeted by this compound.

## Results

### DPMP elicits *CaBP22* ^*−333*^ *::GUS* expression

Synthetic elicitors identified by our high throughput screen can be categorized into several structural classes. One structural class, phenyl-imino-methyl-phenol derivatives (PMPs) and related compounds, are represented by more than 30 members in our original set of 114 synthetic elicitor candidates. PMPs share a phenyl-imino-methyl-phenol skeleton. The PMP, 2,4-dichloro-6-{(E)-[(3-methoxyphenyl)imino]methyl}phenol (DPMP) particularly strongly induced *CaBP22*^*−333*^*::GUS* expression. One week-old liquid-grown Arabidopsis *CaBP22*^*−333*^*::GUS* seedlings continuously exposed to DPMP at a concentration as low as 1 μM for 24 h exhibited GUS reporter gene expression (see Supplementary Fig. S1a). We further tested for possible synthetic elicitor-induced phytotoxicity by trypan blue staining of *CaBP22*^*−333*^*::GUS* seedlings 24 h after incubation with various concentrations of DPMP (see Supplementary Fig. S1b). The results shown in Supplementary Fig. S1a and b can be compared, as the same types of treatment were performed. Seedlings treated with a concentration of 500 μM DPMP stained dark blue, indicating extensive cell death. While some cell death may occur at intermediate concentrations (50 μM and 100 μM), no cell death was observed at lower concentrations (1 μM, 3 μM and 10 μM), which induce *CaBP22*^*−333*^*::GUS* expression. Thus, DPMP-mediated phytotoxicity is not responsible for expression of this defense-associated reporter gene, but it may be the cause of the observed decline of GUS activity at higher concentrations.

### DPMP induces rapidly and transiently disease resistance of Arabidopsis against *Hpa*

**Figure 1:**
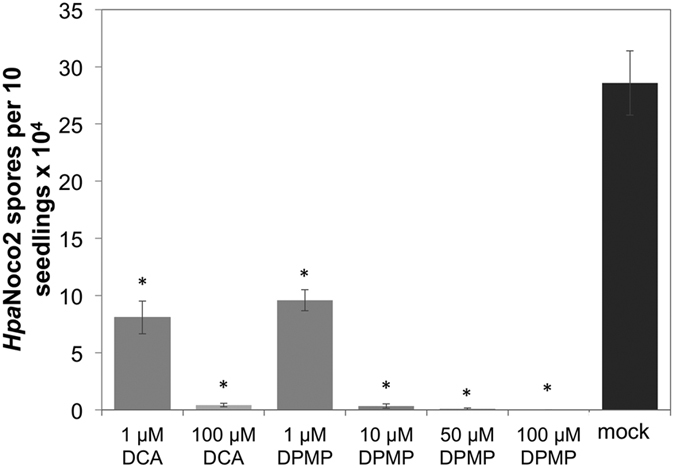
Dose-response analysis of DPMP-induced immunity of Arabidopsis against *Hpa*. Dose response curve for DPMP-elicited immunity against *Hpa*. Three-week-old seedlings were sprayed once with DPMP or DCA at the indicated concentrations or the mock solution (solvent only) 24 h prior to *Hpa*Noco2 (3 × 10^4^ spores mL^−1^) spray infection. All DPMP or DCA concentrations contain same amount of DMSO (0.2% DMSO). The same concentration of DMSO was used as mock treatment. *Hpa* spores were counted 7 days post infection. Data from three independent experiments were analyzed by one-way analysis of variance (one-way AOV) followed by levene’s homogeneity test. Dunnett’s test was used to compare mock vs treatments (P < 0.001). Spore numbers that are significantly reduced after synthetic elicitor treatments compared to mock-treatments were indicated by asterix.

We further examined whether DPMP induces disease resistance against the pathogenic oomycete *Hpa* in soil-grown plants. Wild type seedlings of the Arabidopsis ecotype Columbia (Col-0) were pretreated with different concentrations of DPMP once by a single foliar spray application 24 h prior to infection with the virulent *Hpa* isolate Noco2 (*Hpa*Noco2). *Hpa* spores were counted 7 days post infection (dpi). Plants sprayed with concentrations as low as 1 μM of DPMP showed a significant reduction in spore production compared to mock-pretreated plants (Fig. 1) and at 10 μM DPMP, this effect reached its maximal level of *Hpa*Noco2 immunity. Compared to our previously characterized synthetic elicitors DCA and BHTC^30^,^31^, DPMP displayed maximal suppression of spore formation at 10 times lower concentrations.

Based on the dose-response data shown in Fig. 1, we estimated the median effective concentration (EC_50_) of DPMP regarding its ability to protect Arabidopsis from *Hpa*Noco2 as 514 nM (see Supplementary Fig. S2). This value is much lower than those estimated for DCA and BHTC, which are 6.5 μM and 5.5 μM, respectively^30^,^31^. Although these EC_50_ values are only estimations based on extrapolations of a small number of measurements, it is clear from our data that the EC_50_ values for *Hpa* protection assays of DCA and BHTC are substantially above 1 μM, while that of DPMP is below 1 μM. These results suggest that DPMP is particularly potent in mediating protection of Arabidopsis against *Hpa* infections. This may be due to efficiency regarding its uptake and/or ability to interact with its targets, but may also have different reasons, as we cannot rule out toxicity of DPMP against *Hpa.*

**Figure 2:**
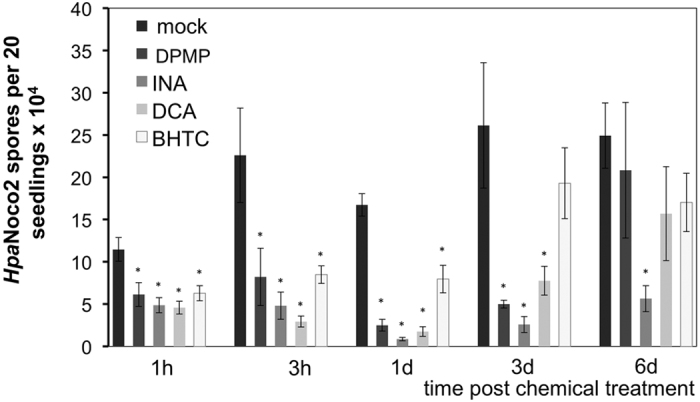
Kinetic analysis of DPMP-induced disease resistance against *Hpa.* Two-week-old Col-0 seedlings were sprayed with 10 μM DPMP or 100 μM INA, DCA or BHTC or mock solution (0.2% DMSO) at the indicated times prior to *Hpa*Noco2 (3 × 10^4^ spores mL^−1^) spray-infection. *Hpa* spores were counted 7 days post infection. Mean and SE values were calculated from a minimum of three biological replicates and the average of those is shown above. Spore numbers that are significantly reduced after synthetic elicitor treatments compared to mock-treatments based on Student *t*-test (p < 0.05) are marked by asterisks.

We further analyzed the kinetics of DPMP-induced defense induction and compared it with the kinetic behavior of other synthetic elicitors. Col-0 plants were pretreated with 100 μM of BHTC, DCA or INA or 10 μM of DPMP at various time points ranging from 1 hour to 6 days prior to pathogen challenge (Fig. 2). At these concentrations each of the tested compounds exhibits maximal activity. Mock treatment itself reduced spore growth when time points between *Hpa*Noco2 infection and chemical treatment were less than one day apart. This effect is likely due to residual liquid coating of plants before being treated with pathogen. At 1-hour post treatment (hpt), all of the tested chemicals reduced *Hpa* spore production (Fig. 2). At 1-day post treatment (dpt), 10 μM DPMP exhibited a similar strength of defense induction as 100 μM DCA or 100 μM INA. Of all of four tested treatments, BHTC showed the weakest activity in inducing disease resistance.

Between 3 and 6 dpt, levels of DPMP-mediated immunity began to decline while

INA-mediated immunity remained constant. At 6 dpt, DPMP did not trigger any significant immunity against the pathogen. Consistent with previous reports, DCA and BHTC also induced plant immunity transiently^30^,^31^, while the activity of INA is long-lasting^27^,^32^,^21^. Taken together, our results clearly showed that the defense-inducing activity of DPMP against *Hpa*Noco2 is stronger than that of other synthetic elicitors we characterized before. Furthermore, its activity is rapid and transient, like that of DCA and BHTC.

### DPMP provides disease protection against the bacterial plant pathogen *Pseudomonas syringae*

**Figure 3:**
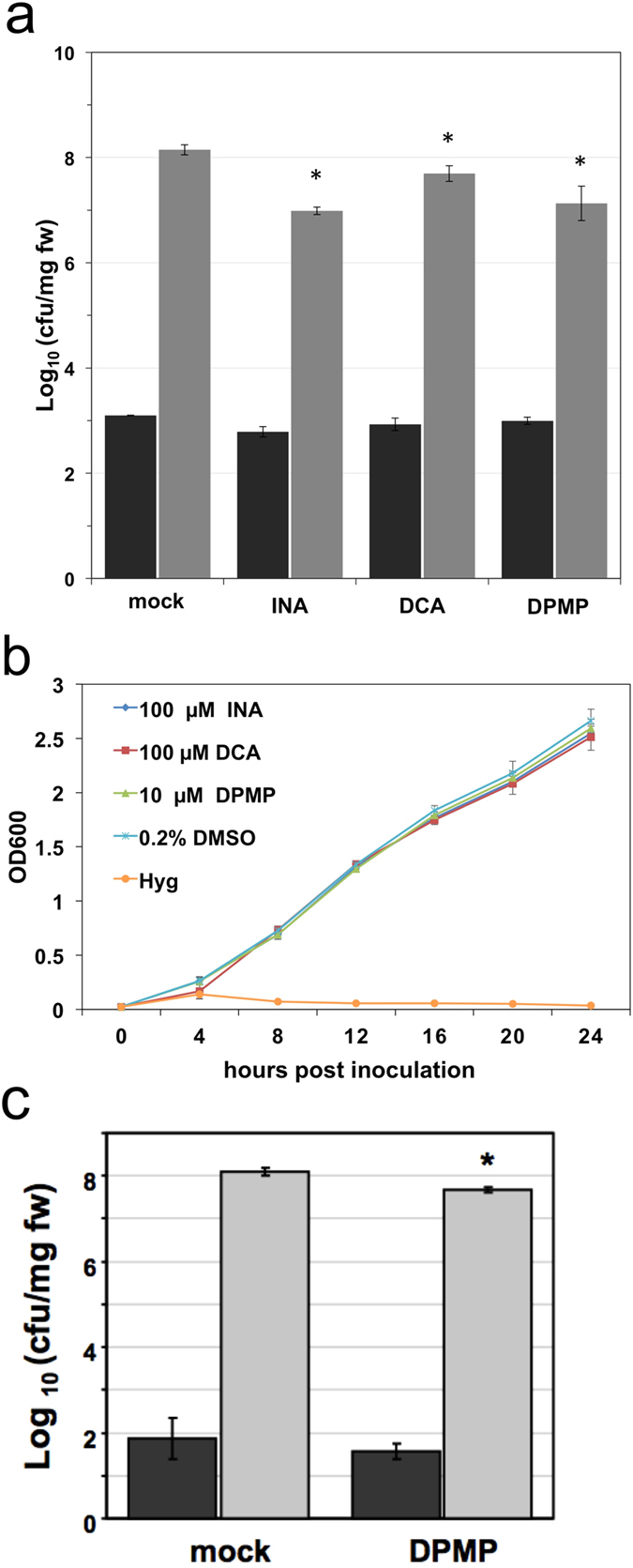
DPMP induces disease resistance against *Pst*. **(a)** Quantification of *Pst* DC3000 growth on Arabidopsis Col-0 plants by the number of colony forming units (cfu). Two-week-old Col-0 seedlings were pre-treated with 10 μM DPMP, 100 μM INA, DCA or mock solution (0.2% DMSO) 24 h prior to dip-inoculation with virulent *Pst* DC3000 (OD_600_ = 0.005). Bacterial titers in the infected tissues were determined at day 0 (black bars) and day 3 (gray bars). Significant differences were identified using Student’s *t*-test (p < 0.05). The error bars are based on technical replicates. The shown data represent a typical example of five nearly identical biological replicates. **(b)** *Pst* DC3000 grown in liquid culture with 10 μM DPMP, 100 μM INA, 100 μM DCA or mock solution (0.2% DMSO) or 100 μg mL^−1^ hygromycin (Hyg). The optical density at 600 nM (OD600), representing the density of bacteria was measured at indicated times after inoculation. Error bars represent the standard error of the mean based on at least 3 independent replicates. **(C)** Tomato plants root drenched with 50 μM DPMP display lower levels of *Ps*t growth in leaves compared to mock-treated (solvent only) plants three days post infection, n  =  3, Student’s *t*-test *p*  =  0.026. The error bars are based on technical replicates. Data shown is representative of at least four independent experiments.

We further tested the ability of DPMP to induce resistance against the virulent bacterial plant pathogen *Pseudomonas syringae* pathovar *tomato* strain DC3000 (*Pst*). Arabidopsis plants were pretreated with synthetic elicitors at concentrations at which they trigger strong immunity against *Hpa* 24 h prior to dip-inoculation with *Pst*. As with INA and DCA, DPMP-pretreated plants showed a significant reduction in bacterial growth (Fig. 3a). To test for a potential direct toxic activity of DPMP against bacteria, we grew *Pst* in liquid medium containing DPMP, other synthetic elicitors or the antibiotic hygromycin. None of the tested synthetic elicitors reduced bacterial growth, while hygromycin completely eliminated growth of *Pst* (Fig. 3b). DPMP is also effective in tomato (*Solanum lycopersicum*) against *Ps*t at a dose at which it is non-toxic for these bacteria. We repeated the experiment shown in Fig. 3b with a DPMP concentration of 50 μM and did not observe toxicity against Pst (data not shown). For our tomato/*Pst* assays, we tested both foliar-spray and root-drench application and found the latter to be more efficient. Tomato plants root drenched with 50 μM DPMP displayed a significant reduction in *Ps*t growth in leaves three days post infection relative to plants treated with a mock solution (Fig. 3c). In previous experiments^31^, 200 μM of BHTC were needed for similar results in tomato against *Ps*t, confirming that DPMP is a more potent synthetic elicitor than BHTC. Taken together, DPMP can induce plant immunity against *Pst* without being directly toxic to this pathogen.

### DPMP interacts with targets operating downstream or independently from SA biosynthesis and is fully dependent on NPR1

**Figure 4:**
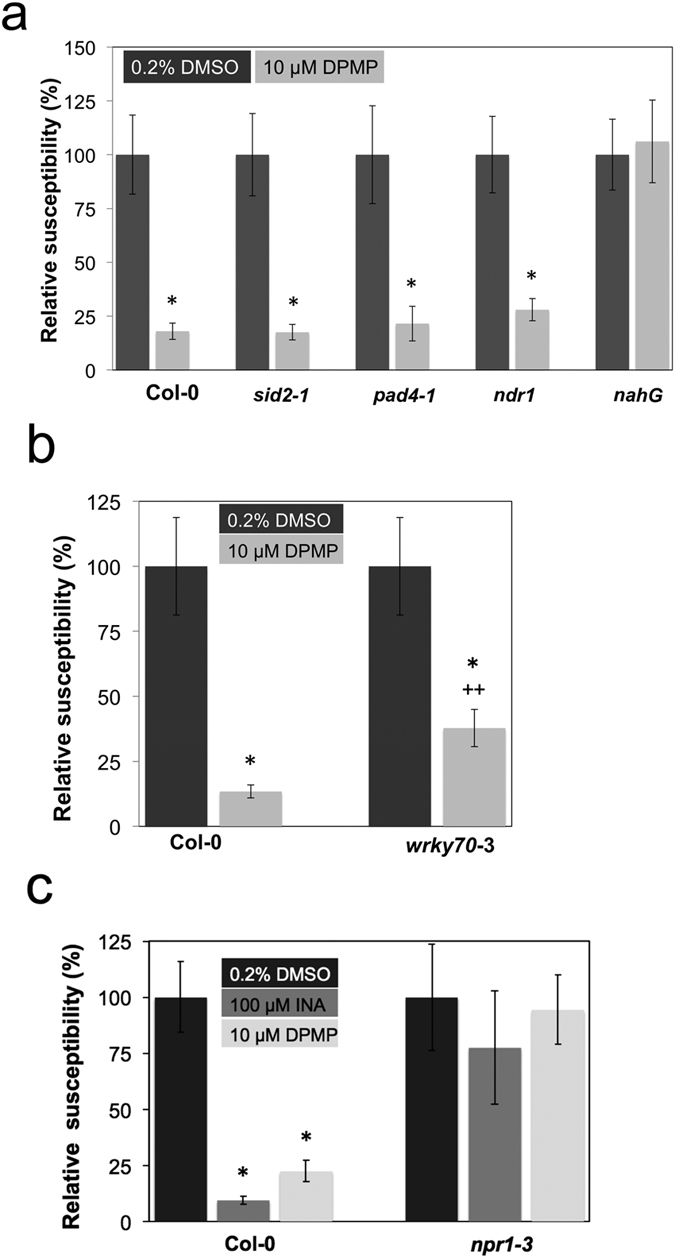
Analysis of DPMP activity in known Arabidopsis defense mutants. (**a,b**) Analysis of DPMP activity in Col-0 plants and Col-0 defense mutants. Three week-old Col-0 seedlings were sprayed with 10 μM DPMP or mock solution (0.2% DMSO) 24 h prior to *Hpa*Noco2 (3 × 10^4^ spores mL^−1^) spray infection. Error bars represent the standard error of the mean based on at least four independent replicates. Relative susceptibility significantly increased in *wrky70* mutants compared to Col-0 after synthetic elicitor treatments based on Student’s *t*-tests (p < 0.05) are marked by a double plus sign (++) (**c**) Analysis of DPMP and INA activity in Arabidopsis Col-0 and the *npr1-3* mutant. Two-week-old Col-0 seedlings were sprayed with 10 μM DPMP, 100 μM INA or mock solution (0.2% DMSO) 24 h prior to *Hpa*Noco2 (3 × 10^4^ spores mL^−1^) spray infection. Error bars represent the standard error of the mean based on at least three independent replicates. In all figures, *Hpa* spores were counted 7 days post infection. 100% equals the respective spore/seedlings value observed in mock-treated controls. Spore numbers that are significantly reduced after synthetic elicitor treatments compared to mock-treatments based on Student’s *t*-tests (p < 0.05) are marked by asterisks.

To determine the mode of action of this new synthetic elicitor, we analyzed the defense-inducing activity of DPMP in the *ndr1-1, pad4-1, sid2-2, npr1-3* and *wrky70-3* Arabidopsis defense mutants as well as the transgenic *nahG* line. While the *ndr1, sid2* and *pad4* mutant plants are known to be compromised in defense-associated SA biosynthesis and the transgenic *nahG* line does not accumulate significant levels of this defense hormone^14^, *npr1* mutants are deficient in the perception of SA^33^,^34^,^35^,^36^. WRKY70 is a transcription factor that partially operates in defense signaling downstream from NPR1 and is partially NPR1-independent^30^,^37^,^38^,^39^. Col-0 and mutants plants were treated with *Hpa*Noco2 24 h after single foliar-spray applications with 10 μM DPMP. *Hpa* spores were counted 7 dpi. DPMP induced strong resistance against *Hpa* in Col-0 wild type plants and the *sid2-2, pad4-1* and *ndr1* mutants (Fig. 4a). DPMP-mediated immunity was slightly, but significantly reduced in the *wrky70* mutant compared to Col-0 (Fig. 4b). However, in *npr1*-3 plants the defense inducing activity of DPMP was fully abolished; similar to that of INA (Fig. 4c). Our lab previously reported that DCA is partially dependent on *NPR1* and *WRKY70*^30^. DPMP is weakly dependent on *WRKY70* and completely dependent on *NPR1*, which discriminates this compound from DCA. Surprisingly, in *nahG* transgenic plants, no significant protection was observed against *Hpa* after the application of DPMP (Fig. 4a). The *nahG* transgenic plants express a SA hydroxylase, an enzyme that converts SA to catechol. It is possible that this enzyme is also converting DPMP or one of its bioactive metabolic products.

### Structure activity analysis of a selection of PMP-related derivatives

**Figure 5:**
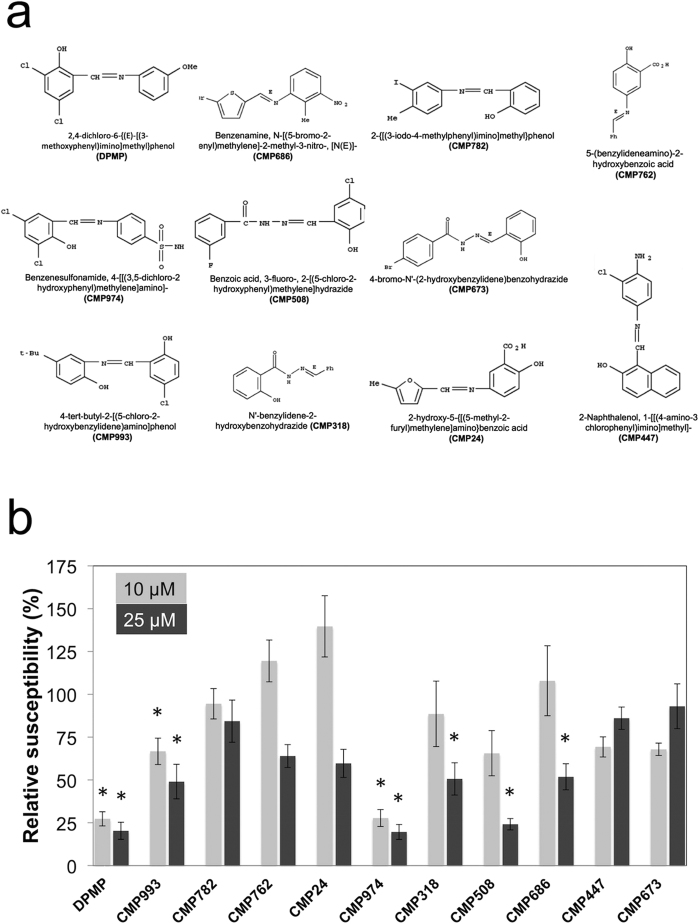
Structure activity analysis of PMP-related compounds. (**a)** Chemical structures of compounds analyzed. **(b)** Relative susceptibility of Arabidopsis against *Hpa*Noco2. Two-week-old seedlings were sprayed with 10 μM or 25 μM of the indicated compounds or their respective mock controls (1% DMSO or 2.5% DMSO respectively) 24 h prior to *Hpa*Noco2 (3 × 10^4^ spores mL^−1^) spray infection. *Hpa* spores were counted 7 days post infection. Shown are relative numbers of spores per seedling compared to values obtained with the respective mock-treated controls. Error bars represent the standard error of the mean based on at least 3 independent replicates. Spore numbers that are significantly reduced after synthetic elicitor treatments compared to mock-treatments based on Student’s *t*-tests (p < 0.05) are marked by asterisks.

DPMP is only one member of a set of 33 PMPs or –related compounds identified by our high throughput synthetic elicitor screen. While not all of these 33 compounds share a common phenyl-imino-methyl-phenol skeleton, they all bear an imine group linked to a phenyl moiety. We tested ten compounds of this set of 33 that were commercially available and compared their defense-inducing activities to DPMP (Fig. 5a,b). Arabidopsis Col-0 plants were pretreated with a concentration of 10 μM or 25 μM of the respective chemicals 24 h prior to *Hpa*Noco2 infection. We observed three major activity trends of these compounds: strong, moderate or weak compared to DPMP, which we considered a strong defense inducer. PMP-related compounds that induced significant levels of immunity against *Hpa* at 10 μM and 25 μM were categorized as “strong”. If they only induced significant protection against *Hpa* at 25 μM, we categorized them as “moderate”. If they did not exhibit any significant defense induction at the tested concentrations, we classified them as “weak” inducers, as they were still able to induce *CaBP22*^*−333*^*::GUS* expression in our initial compound screen (data not shown).

4-[[(3,5-dichloro-2-hydroxyphenyl)methylene]amino] benzenesulfonamide (CMP974) and 4-tert-butyl-2-[(5-chloro-2-hydroxybenzylidene)amino]phenol (CMP993), provided strong protection against *Hpa* infection. CMP974 and CMP993 suppressed *Hpa* spore growth at concentrations of both 10 μM and 25 μM and levels of protection mediated by them reached up to 80% and 50%, respectively.

Benzoic acid, 3-fluoro-2-[(5-chloro-2-hydroxyphenyl)methylene]hydrazide (CMP508), N′-benzylidene-2-hydroxybenzohydrazide (CMP318), Benzenamine, N-[(5-bromo-2-thienyl)methylene]-2-methyl-3-nitro-[N(E)]- (CMP686) mediated moderate levels of defense induction and reduced susceptibility only at the 25 μM concentration to 75% to 50%. Although the five remaining PMP-related compounds did not trigger defense induction at concentrations of 10 μM or 25 μM, and are considered “weak” inducers, they may induce plant defense at higher concentrations. The strongest compounds of the 10 tested candidates are true PMPs structurally similar to DPMP, while the class of moderate defense inducers also includes CMP318 and CMP686, compounds that deviate from the PMP core structure.

### Synthetic elicitor activity of possible metabolic products of DPMP

The imine bond of PMPs may be subject to hydrolysis in the aqueous environment of biological tissues and cells^40^. Members of the imine bond containing compound class imprimatin C1 are known to remain stable in aqueous solution before application to plants and the synthetic auxin sirtinol, which also contains an imine bond, is hydrolyzed after uptake into cells^41^,^42^.

We tested whether the molecular structure of DPMP dissociates in aqueous solution *in vitro*. NMR analysis combined with HSQC (Heteronuclear Single Quantum Coherence) showed that DPMP is not hydrolyzed in aqueous medium *in vitro* (data not shown). However, upon uptake by plant tissues, DPMP may get hydrolyzed and further converted by plant enzymes and the resulting products may be the actual biologically active compounds *in vivo.*

**Figure 6:**
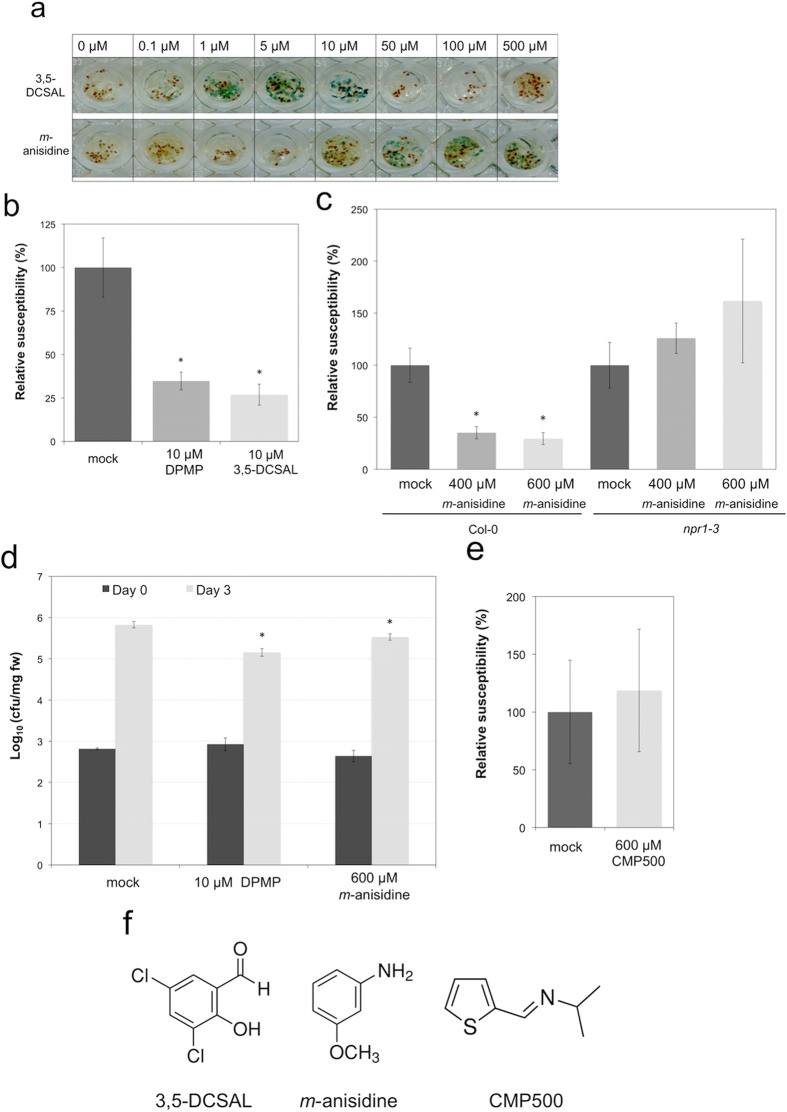
Analysis of possible metabolic products of DPMP and PMP bridge activities. **(a)** X-Gluc histochemical stainings were performed according to protocol described in Material and Methods. Blue/green color of cotyledons indicates induction of the *GUS* gene expression. All histochemical staining analyses were performed three times with similar results. Shown are typical examples. **(b)** Two-week-old seedlings were sprayed with 10 μM DPMP, 10 μM 3,5-DCSAL or mock control (1% DMSO) 24 h prior to *Hpa*Noco2 spray infection. *Hpa* Spores were counted 7 dpi. Mean and SE values were calculated from a minimum of five biological replicates and the average of those is shown above. Spore numbers that are significantly reduced based on Student *t*-test (p < 0.05) are marked by asterisks. **(c)** Two-week-old seedlings were sprayed with 400 μM *m*-anisidine, 600 μM *m*-anisidine or mock control (H_2_O) 24 h prior to *Hpa*Noco2 spray infection. *Hpa* Spores were counted 7 dpi. Mean and SE values were calculated from three biological replicates and the average of those is shown above. Spore numbers that are significantly reduced based on Student *t*-test (p < 0.05) are marked by asterisks. **(d)** *M*-anisidine induces disease resistance against *Pst*. Quantification of *Pst* DC3000 growth on Arabidopsis Col-0 plants by cfu. Two-week-old Col-0 seedlings were pre-treated with 10 μM DPMP, 600 μM *m*-anisidine or mock solution (0.2% DMSO) 24 h prior to dip-inoculation with virulent *Pst* DC3000 (OD_600_ = 0.005). Bacterial titer was evaluated at day 0 (black bars) and day 3 (gray bars). Significant differences were tested using Student *t*-test (p < 0.05). The error bars are based on technical replicates. The shown data represent a typical example of two identical biological replicates. **(e)** Two-week-old seedlings were sprayed 600 μM CMP500 or mock control (H_2_O) 24 h prior to *Hpa*Noco2 spray infection. *Hpa* Spores were counted 7 dpi. Mean and SE values were calculated from three biological replicates and the average of those is shown above. Spore numbers that are significantly reduced based on Student *t*-test (p < 0.05) are marked by asterisks. **(f)** Chemical structures of the compounds analyzed.

Therefore we tested 3,5-dichlorosalicylaldehyde (3,5-DCSAL) and *m*-anisidine (Fig. 6f). While m-anisidine should get released upon hydrolysis of DPMP, the production of 3,5-DCSAL should require an additional oxidation step. We first tested the ability of 3,5-DCSAL and *m*-anisidine to induce GUS expression in transgenic *CaBP22*^*−333*^*::GUS* Arabidopsis plants (Fig. 6a). 3,5-DCSAL induced expression of this pathogen-responsive reporter gene at the same concentration as DPMP (1–10 μM). At higher concentrations of 3,5-DCSAL (50 μM to 500 μM), the observed decline of GUS activity may be cause by 3,5-DCSAL -mediated phytotoxicity.

Surprisingly, *m*-anisidine also induced GUS expression in this plant line, albeit at much higher concentrations (50–500 μM). To confirm these results, we tested different concentrations of 3,5-DCSAL and *m*-anisidine in *Hpa* defense assays and compared their bioactivity to DPMP. At a concentration of 10 μM, 3,5-DCSAL exhibits a similar strength of defense activation as 10 μM DPMP against *Hpa* (Fig. 6b). *M*-anisidine does not trigger defense induction at a concentration of 100 μM (data not shown). However, at higher concentrations (400 μM and 600 μM) *m*-anisidine triggers immunity against *Hpa* and, like DPMP, its defense-inducing activity is completely dependent on *NPR1* (Fig. 6c). Much lower concentrations of m-anisidine were sufficient for the *CaBP22*^*−333*^*::GUS* expression studies shown in Fig. 6a than that for the *Hpa* defense assays shown in Fig. 6c. This difference is due to the nature of the respective assays. While the reporter gene assays were done under continuous exposure to the compound for 24 h, the synthetic elicitors were only applied once by single spray application in the *Hpa* assays. In addition to protection against *Hpa, m*-anisidine induced plant immunity against *Pst* (Fig. 6d). In order to test whether the imine bridge of PMPs itself is sufficient to induce immunity against *Hpa*Noco2, we analyzed N-[(E)-2-Thienylmethylidene]-2-Propanamine (CMP500) (Fig. 6f), a molecule of relative simple structure that contains an imine bridge. CMP500 did not induce detectable resistance of Arabidopsis Col-0 against *Hpa*Noco2 at the concentration we tested (Fig. 6e). Taken together these results show that, the PMP imine bridge structure itself seems not to trigger immunity. However, two possible metabolic products of DPMP have synthetic elicitor activity. As 3,5-DCSAL is of similar efficiency as DPMP, this potential metabolic product of DPMP may be mainly responsible for the observed immunity after DPMP application. Although we cannot exclude, that in plant tissues 3,5-DCSAL gets further oxidized to 3,5 Dichlorosalicylic acid, this aldehyde proved to be a powerful synthetic elicitor.

### Transcriptome changes triggered by DPMP in Arabidopsis shoots and roots

To profile global transcriptional patterns associated with DPMP-mediated defense activation in Arabidopsis roots and shoots separately, we performed mRNA-seq analysis. In soil grown plants, separating these plant parts from each other without any contamination by dirt or excessive wounding is difficult. Therefore Arabidopsis plants were grown for 14 days on ½ MS agar plates containing either 3 μM DPMP or mock solution (solvent only) and their shoot and root tissues were analyzed separately. This condition was chosen because continuous exposure to 3 μM DPMP triggers strong GUS expression in *CaBP22*^*−333*^*::GUS* plants (see Supplementary Fig. S1). We performed two independent biological replicates and sequenced the respective libraries using the Illumina HiSeq2500 platform. Differentially expressed genes (DEGs) after DPMP treatment were identified by comparing read counts to those observed in the respective mock-treated control samples using a Bonferroni-corrected false discovery rate (FDR)-cut off of 0.05.

In shoots, treatment with DPMP significantly altered transcript levels of 1364 genes, 1061 of which were transcriptionally up-regulated (DPMP-shoots-up) and 303 were transcriptionally down-regulated (DPMP-shoots-down) relative to the mock controls (see Supplementary Table S1). DPMP treatment in shoot tissue resulted in a typical defense-associated transcriptional profile. Standard defense marker genes, such as *PR1, PR5, CaBP22* and *LURP1*, as well as numerous WRKY transcription factor genes were transcriptionally up-regulated. Enriched gene ontology (GO) terms were calculated by the Botany Array Resource classification super viewer [ http://bar.utoronto.ca/welcome.htm;^43^] and suggested that collective roles of DPMP-shoots-up genes are in “response to stress” and “abiotic/biotic stimuli” as well as “signal transduction” (Table 1). Furthermore, the analysis of promoter motifs of these genes using the TAIR motif analysis tool (https://www.arabidopsis.org/tools/bulk/motiffinder/index.jsp) revealed that these DPMP-shoots-up genes are highly enriched for known defense-associated promoter motifs such as the hexameric motif TTGACT (p = 2.92E-38) that matches the WRKY-binding W box element (TTGACC/T)^44^. Also, a TGA box core motif (TGACG) was represented in the significantly enriched hexamer TTGACG (p = 2.41E-05). Interestingly, DPMP-shoot-down genes feature, along with “response to stress” and “abiotic/biotic stimuli”, the additional enriched GO term “electron transport or energy pathways”, which also includes genes involved in photosynthesis (see Supplementary Table S1). Defense responses are associated with increased demands for energy and plant respiration is highly stimulated during plant defense responses^45^. In contrast, studies on photosynthesis and plant defense have shown that photosynthetic metabolism is repressed locally during plant defense^45^,^46^,^47^. It has been suggested that this might be due the high sensitivity of the photosynthetic apparatus for ROIs produced during the defense-associated oxidative burst^45^.

**Figure 7:**
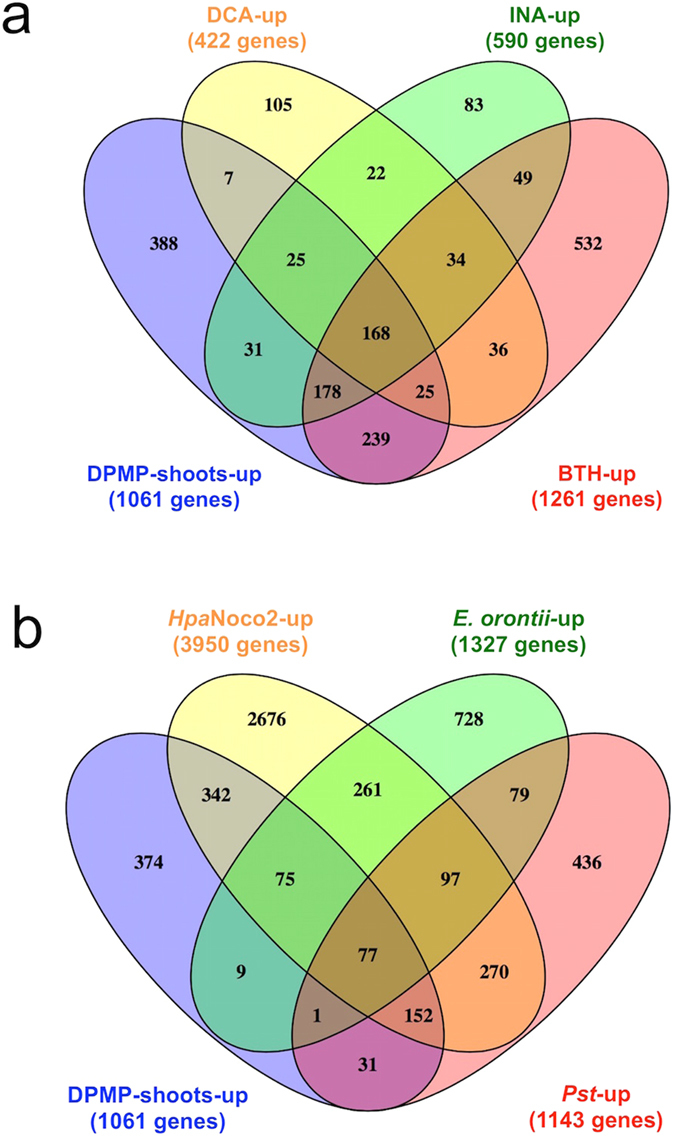
DPMP-triggered transcriptome changes. Venn diagram analysis highlighting differences and similarities between the gene sets that were up-regulated by 3 μM DPMP and the SA analogs DCA, INA or BTH **(a)**, set of Arabidopsis genes up-regulated by *E. orontii*^49^*, Hpa*^50^ and *Pst*^51^** (b).**

Comparison of sets of genes that were induced by pathogens or known elicitors revealed that 63% of all DPMP-shoots-up gene members are also inducible by the SA analogs DCA, INA and/or BTH^30^,^48^. This may indicate that, like DCA, INA and BTH, DPMP also acts as a partial agonist of SA and mimics some SA functions (Fig. 7a). Additionally, 62% of all DPMP-shoots-up gene members are also responsive to infections with the oomycete *Hpa,* the bacterium *P. syringae* and/or the powdery mildew fungus *Erysiphae orontii* (Fig. 7b). Transcriptional responses triggered by these virulent pathogens are associated with basal defense^49^,^50^,^51^.

We previously described the *ACID (Associated with Chemically Induced Defense*) cluster as a set of genes strictly associated with defense activation by two separate synthetic elicitors, DCA and INA^30^. Comparison of this cluster with the set of high dose (hd)-BHTC-shoots-up genes, we recently described^31^, and the set of DPMP-shoots-up defined a set of 75 genes that are commonly up-regulated by four distinct synthetic elicitors (DCA, INA, BHTC and DPMP). We termed this set *SUPER-ACID*s (see Supplementary Fig. S4 and Supplementary Table S1), as its members are particularly tightly associated with chemically induced disease resistance. Enriched gene ontology (GO) terms^43^ suggested that collective roles of *SUPER-ACID*s are in “response to stress” and “abiotic/biotic stimuli” as well as “signal transduction” (Table 1). Also the *SUPER-ACID* cluster contains many known defense-related genes and is highly enriched for genes associated with kinase activity (p = 2.808e-08) and the cellular components of plasma membrane (p = 5.920e-05), extracellular (p = 8.781e-03) and nucleus (p = 0.044). Furthermore, the analysis of promoter motifs of this cluster using the TAIR motif analysis tool revealed that these *SUPER-ACID*s are highly enriched for known defense-associated promoter motifs such as the hexameric motif TTGACT (p = 9.37e-151) that matches the WRKY-binding W box element (TTGACC/T)^44^. Also, a TGA box core motif (TGACG) was represented in the significantly enriched hexamer TTGACG (p = 297e-80). Additionally, almost all (97%) *SUPER-ACID* members are also responsive to infections with *Hpa, Pst*and/or *E. orontii* (see Supplementary Fig. S5). All of these results support the conclusion that *SUPER-ACID*s are important for disease resistance, and tightly associated with successful pathogen defense.

In roots, the number of DPMP-responsive DEGs was lower (558 DEGs), with 207 up-regulated (DPMP-roots-up) and 351 down-regulated genes (DPMP-roots-down) (see Supplementary Table S1 & Supplementary Fig. S3). However, the set of DPMP-roots-up genes shares similar putative collective roles with DPMP-shoots-up members. As in the case of DPMP-treated shoots, enriched gene ontology (GO) terms showed that this set also contains genes with likely collective roles in “response to stress” and “abiotic/biotic stimuli” as well as “signal transduction” (Table 1). Moreover, this set also contains established defense marker genes like *PR2, LURP1, CaBP22* and *WRKY* genes. Genes down-regulated by DPMP treatment in root tissues further contain genes related to “response to stress” and “abiotic/biotic stimuli”. These results indicate that the role of DPMP-root members is also likely related to defense responses.

### DPMP induces hormesis-like responses similar to BHTC in Arabidopsis

**Figure 8:**
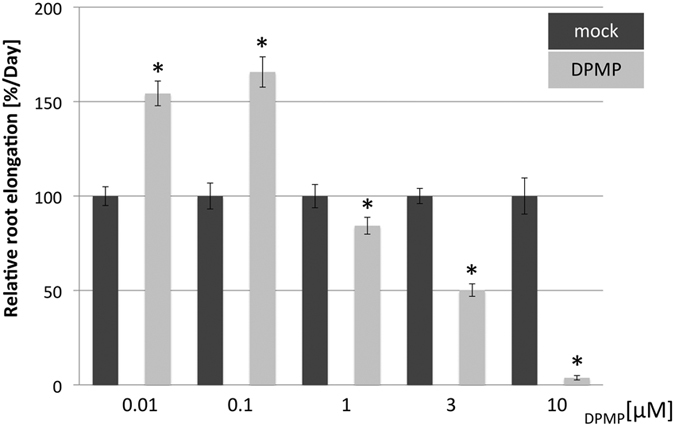
Relative root length of Col-0 plants grown on DPMP. Plants were grown on indicated concentrations of DPMP-containing ^1^/_2_ MS agar plates or the respective control (solvent only) for 14 days and the average relative changes on root length per day compared to mock treatment. Significant differences between DPMP and control-treated plants were determined by Student’s *t*-tests (p < 0.05) and are marked by asterisks.

Interestingly, similar to the synthetic elicitor BHTC, DPMP significantly enhanced root length of plate-grown Arabidopsis plants at concentrations below 1 μM, while high doses of DPMP reduced Arabidopsis root growth. In these assays, plants were grown on various concentrations of DPMP-containing ^1^/_2_ MS agar plates for 14 days. At concentrations 0.01 μM and 0.1 μM DPMP significantly enhanced the root length of Arabidopsis plants, while at concentrations above 1 μM inhibition of root growth was observed (Fig. 8). The reduction of root growth on 10 μM DPMP may be due to phytotoxic effects of this compound or due to re-allocation of metabolic resources away from growth-related processes to defense induction. Our data shown in Supplementary Fig. S1 suggest that DPMP at this concentration is not toxic to Arabidopsis. Interestingly, growth penalty effects are commonly observed when plant immunity is constantly induced due to mutations^52^,^53^. Thus, most likely DPMP causes at high doses reallocation of critical resources to defense reactions. This can be clearly seen by the massive induction of defense gene expression.

## Discussion

In this study, we identified and characterized the phenyl-imino-methyl-phenol (PMP) derivative DPMP as a particularly potent novel synthetic elicitor. DPMP strongly induced *CaBP22*^*−333*^*::GUS* reporter gene expression. Furthermore, it induced disease resistance against two phylogenetically distinct pathogens (*Hpa*Noco2 and *Pst*). Its defense-inducing activity is transient and strong, as it activates immune reactions at concentrations 10-fold lower than most previously identified SA analogs without being directly toxic to pathogens. DPMP is the most potent compound of those synthetic elicitor candidates we so far characterized^20^,^30^,^31^ with an unusually low estimated EC_50_ value of 514 nM. Low active concentrations are often correlated with high target affinity, high target specify and low levels of undesired side effects^54^. Interestingly, DPMP consists of two separate moieties, which could be released in planta and independently induce plant immune responses. Both of these moieties are linked by a labile imine bridge, which can subject to hydrolysis in the aqueous environment this compound encounters in plant tissues.

Our mode-of-action analysis of DPMP using Arabidopsis defense mutants revealed that DPMP acts downstream from SA biosynthesis and SA accumulation or acts independently from these defense-related processes. However, the defense-inducing activity of DPMP was completely blocked in the *npr1-3* mutant and partially reduced in the *wrky70-3* mutant. Based on these results, we propose that DPMP activates the NPR1-dependent branch of the defense-signaling network. It seems further to partially require WRKY70-dependent defense signaling processes for its activity. Although, DPMP activity is completely dependent on NPR1, similar to that of SA and the well-characterized SA analogs INA and BTH^21^,^22^,^26^,^29^,^55^, its active concentration is 10-fold lower than that of INA and its activity is transient unlike that of INA and BTH, which induce sustained disease resistance in plants.

Furthermore, DPMP is functionally distinct from our previously characterized synthetic elicitors DCA and BHTC. Unlike DCA, it is completely dependent on NPR1 and unlike BHTC, it is still active in *wrky70-3* plants. Based on its low EC_50_ value, the affinity of DPMP for its target protein(s) might be higher than that of other synthetic elicitors, such as INA, DCA or BHTC. Differences in the genetic requirements for synthetic elicitor activity further suggest that DPMP interacts either with different targets than DCA, BHTC or INA, or that it interferes with common targets of these synthetic elicitors in a distinct manner. Combined, these results made DPMP an interesting new bioactive compound for further studies.

With 3,5-DCSAL and *m*-anisidine we identified two possible metabolic conversion products of DPMP, which independently possess synthetic elicitor activity. It is possible that 3,5-DCSAL gets further oxidized to 3,5-dichloro salicylic acid (3,5-DCSA) *in planta*. 3,5-DCSA is a known active analog of SA and has been tested along with other SA derivatives in studies on plant defense induction^24^,^56^. It was shown that 3,5-DCSA, 4-chloro salicylic acid (4-CSA), and 5-chloro salicylic acid (5-CSA), functionally mimic SA in plants, induce *PR1* gene expression and enhance disease resistance to TMV infection in tobacco^24^. Also, 3,5-DCSA primed Arabidopsis plants for enhanced induction of defense similar to SA, BTH and the SA-derivatives 4-CSA, and 5-CSA^57^,^58^,^59^,^60^. Wu *et al.*^61^ reported that NPR1 directly binds to SA in an equilibrium dialysis assay with the Kd value about 140 nM. In addition to SA, they showed that 4-CSA, 5-CSA and 3,5-DCSA bind to NPR1 with similar or slightly higher affinity than SA but not inactive analogs of SA (catechol, methyl-salicylate, 4-hydroxy benzoic acid and 3-hydroxy benzoic acid)^61^. This observation is also consistent with our finding that the activity of DPMP is blocked in *npr1-3* mutants. Since release of 3,5-DCSAL may be mainly responsible for the defense-inducing activity of DPMP, this molecule may act after oxidation to 3,5-DCSAL by binding to NPR1.

The imprimatins C1 and C2, which also contain imine bonds, and their potential breakdown products 4-chlorobenzoic acid (4-CBA) and 3,4-dichlorobenzoic acid (3,4-DCBA) were identified as enhancers of pathogen-induced cell death in Arabidopsis suspension culture as well as inducers of disease resistance against both avirulent and virulent *Pst* in Arabidopsis^41^. It was also shown that 3,5-dichlorobenzoic acid (3,5-DCBA) exhibits a stronger defense-inducing activity than 3,4-DCBA and that 3,4-DCBA is stronger than 4-CBA^30^,^41^. These results indicate that the levels of bioactivity of these compounds depend on the number and the position of chlorine substituents at their benzene rings. At the 3-, 4- and 5-positions chlorines seem to enhance defense activation. 3,5-DCBA shares a common dichlorinated benzoic acid core structure with INA and DCA. The exchange of a carbon atom by a nitrogen atom at position 4 of the ring converts 3,5-DCBA to INA, while the addition of an amino group to a position 2 of the ring converts it to DCA. Compared to 3,5-DCBA, DCA and INA more efficiently induced *CaBP22*^*−333*^*::GUS* expression and defense activation against *Hpa* in our assays^30^ and DPMP is more potent than both DCA and INA.

To our knowledge, we showed here for the first time that DPMP and one of its possible metabolic products, 3,5-DCSAL, induce basal defense against virulent *Hpa*Noco2 and that DPMP induces plant defense against *Pst*. DPMP and 3,5-DCSAL share a dichloronated aromatic six-member ring, in addition to a hydroxyl group at position 2. This substituted benzene ring core structure represents the most efficient synthetic elicitor class (regarding efficiency against *Hpa* and *Pst* in Arabidopsis) we have tested so far. Although Wu and co-workers^61^ found SA and SA derivatives to show similar levels of NPR1 affinity in their *in vitro* binding studies, our results, along with those of others, suggest that subtle structural differences of these compounds affect their *in vivo* activity and, thus, the strength of the disease resistance they mediate against pathogens. Such structural differences may alter affinities of these molecules for target proteins under *in vivo* conditions. Alternatively, these changes might affect the efficiency of uptake, metabolic conversions or systemic dispersal they encounter *in planta* or their *in vivo* stability.

In addition to 3,5-DCSAL, a second possible metabolic product of DPMP, *m*-anisidine, also has defense-inducing activity. At concentrations of 400 μM and 600 μM, *m*-anisidine mediated immunity against *Hpa* and *Pst* (Fig. 7c,d). To our knowledge, *m*-anisidine has not been described as plant defense inducer before. Similar to DPMP this compound also requires NPR1 for defense induction.

DPMP is only one PMP representative identified in our original high throughput synthetic elicitor screen. We found DPMP and CMP974 to be the most efficient defense inducers of a set of 11 tested PMP-related compounds. Both molecules contain a 3,5-DCSAL-related moiety, which most likely is responsible for their strong defense inducing activity. Four additional PMP-related compounds protected Arabidopsis against *Hpa*. However, these compounds are less efficient than DPMP and CMP974. Two of them, CMP993 and CMP508, contain moieties that are similar to 3,5-DCSAL featuring an aromatic six-member ring with a hydroxyl group and a chlorine at position 5. The lack of an additional chlorine may be the reason for the weaker defense induction compared to 3,5-DCSAL-containing PMPs. The remaining two moderately active PMP-related defense inducers, CMP318 and CMP686, do not contain moieties similar to 3,5-DCSAL or *m*-anisidine. Five additional PMP-related compounds we tested did not trigger defense against *Hpa* at the tested concentrations and they seem less active compared to others. However, they may induce efficient plant defense responses at higher concentrations. Taken together, PMPs and related compounds are a large and structurally diverse class of synthetic elicitors that have the potential to be highly efficient.

In both shoots and roots, the application of DPMP triggered typical defense-associated transcriptional profiles. The set of DPMP-shoots-up genes largely overlap with genes induced by the known SA mimics DCA, INA and BTH defining DPMP as a new SA analog. Although DPMP seems to acts as a partial agonist of SA mimicking some SA functions, it also induces expression of 388 genes that are uniquely targeted by this compound.

Interestingly, we found that, like BHTC, DPMP triggered hormetic effects and significantly enhanced root length of plate-grown Arabidopsis plants when applied at concentrations below 1 μM, while high doses of DPMP reduced Arabidopsis root growth and induced defense gene expression. The hormetic effects of BHTC were tested in a set of defense and auxin signaling-mutants. These data suggested that the WRKY70 transcription factor contributes to BHTC-induced immunity along with hormetic root elongation. Although, most of the tested auxin-signaling mutants did not exhibit clear effects on BHTC-mediated hormesis, the *axr1-3* and *slr-1* mutants were compromised in this response^31^. Further studies with DPMP, in addition to BHTC, may reveal common and distinct roles of these two synthetic elicitors in hormesis and may also uncover links between plant immunity and hormetic growth effects.

## Materials and Methods

### Arabidopsis Growth Conditions, Plant material, Pathogen Infections and Tissue-Staining

Arabidopsis *(Arabidopsis thaliana*) plants were grown on soil or media under fluorescent lights (16 h of light/8 h of dark, 23 °C, 100 μE m^−2^ s^–1^) unless otherwise noted. The Arabidopsis mutants *wrky70-3*^39^, *pad4-1*^33^, *ndr1-1*^62^*, sid2-2*^63^, *npr1-3*^35^,^36^, *nahG*^64^ have been described. *Hyaloperonospora arabidopsidis (Hpa*) was grown and propagated as described previously^65^. *Hpa* infection assays were performed with either 2- or 3 week-old Arabidopsis seedlings. Although we have not observed any effect of this age-difference on the outcome of our experiments, we consistently performed each set of experiments (including all controls) with seedlings of a defined age (2- or 3-week old). Two- or three-week old Arabidopsis plants were spray-infected with *Hpa*Noco2 spore suspensions at 3 × 10^4^ spores ml^–1^ with Preval sprayers (http://www.prevalspraygun.com). Plants were scored for *Hpa* growth 7 days post infection (dpi) by counting spores/seedlings using a hemicytometer to determine the spore density of a suspension of 10 or 20 infected seedlings per 1 ml of water. The Student’s *t-*test was used to determine if the effects of the mutations or chemical treatments on sporulation were statistically significant. Trypan blue staining was performed as described previously^65^.

Arabidopsis plants were dip inoculated with *Pseudomonas syringae* pv *tomato* DC3000 (*Pst*) with an indicated inoculum concentration (optical density at 600 nm). For these experiments, infections and scoring were performed as described previously^66^. Plants were also visually scored for disease symptoms 2 days after inoculation. To test for a potential direct toxic activity of DPMP against bacteria *Pst* DC3000 grown in liquid culture with relevant synthetic elicitor, mock solution or 100 μg mL^−1^ hygromycin (Hyg). The optical density at 600 nM was measured at 0 hour (h), 4 h, 8 h, 12 h, 16 h, 20 h, 24 h after inoculation^30^.

### Pathogen infection experiments with tomato

Tomato (*Solanum lycopersicum*) plants cv. Moneymaker were grown, *Ps*t-infected and treated as previously described^31^ with either 50 μM DPMP or a mock treatment containing DMSO solvent.

### Analysis of GUS Activity and Treatment of Homozygous *CaBP22* ^–333^ *-promoter*::*GUS* with Synthetic Elicitor

Arabidopsis seedlings were grown in 96-well plates, treated with synthetic elicitors, and then stained (histochemically) for *GUS* expression as was previously described^30^.

### Synthetic Elicitors

DPMP (CSA# 303770-66-3), 3,5-DCSAL (CSA# 90-60-8) and *m*-anisidine (CSA# 536-90-3), CMP500 (MDL# MFCD00728745) and CMP974 (CSA# 20114-85-6) were all ordered from Sigma-Aldrich (https://www.sigmaaldrich.com). CMP993 (CSA# 414901-51-2), CMP762 (CSA# 857254-99-0), CMP24 (CSA# 414906-43-7), CMP508 (CSA# 350509-46-5), CMP686 (CSA# 1164456-88-5), CMP447 (CSA# 414880-47-0), CMP673 (CSA# 125741-51-7) were ordered from Interchim (https://www.interchim.com). CMP782 (CSA# 414897-80-6) and CMP318 (CSA# 59395-02-7) were purchased from Ryan Scientific (https://www.ryansci.com).

### Synthetic Elicitor Treatment before Pathogen Infection

Stock solutions of all synthetic elicitors were prepared in 100% DMSO. Stock solutions were diluted in water and 2 ml/pot sprayed on soil-grown plants at the indicated times and concentrations with Preval sprayers. Final DMSO concentrations never exceeded 2.5%. To test for chemically induced disease resistance, the plants were sprayed with 2 ml/pot of chemicals at the indicated concentrations and times prior to pathogen challenge. Disease symptoms were analyzed as described above.

### Arabidopsis Root Growth Assays

Col-0 seeds were surface-sterilized in a 75% ethanol then 0.02% Triton X, 10% bleach and water solution, for 10 and 15 min respectively. Seeds were then rinsed with sterile water and plated on solid media containing: ½ MS (Murashige and Skoog), 1.5% agar, 3% sucrose and defined concentrations of synthetic elicitors or the equivalent concentration of DMSO (control). Seeds were stratified for two days at 4 °C and then placed vertically under fluorescent lights. Plates were scanned at 3, 5, 7, 11, and 14 days after stratification and root lengths were measured using ImageJ^67^.

### Transcriptome-profiling by mRNA-seq of plate-grown Arabidopsis seedlings

Col-0 seeds were surface-sterilized in 75% ethanol and a 0.02% Triton X, 10% bleach solution, for 10 and 15 min respectively. Seeds were then rinsed with sterile water and plated on solid media containing ½ MS (Murashige and Skoog), 1.5% agar, 3% sucrose and 3 μM of BHTC or solvent only (0.1% DMSO). Seeds were stratified for two days at 4 °C and then placed on plates which were vertically positioned under fluorescent lights. After 14 days, plant tissue was separated into shoot and root parts using a blade. To prevent any tissue contamination, seedlings were cut into three parts, and root-shoot intersection areas were discarded. Total RNA was isolated from shoot and root separately by using TRIZOL (Invitrogen, http://www.invitrogen.com). RNA was processed and libraries were prepared with the NEBNext Ultra RNA library prep kit by following manufacturer’s instruction (New England Biolabs, http://www.neb.com). For each treatment type root and shoot tissues were separately analyzed. We performed two independent biological replicates for each experimental condition and sequenced the respective libraries using the Illumina HiSeq2500 platform. Sequence reads were analyzed using TopHat for alignment of reads to the TAIR10 Arabidopsis genome annotation. Differentially expressed genes (DEGs) were identified by comparing read counts from DPMP-treated samples versus their respective mock controls by EdgeR using a Bonferroni-corrected false discovery rate (FDR)-cut off of 0.05. All mRNA-seqdata generated for this study were deposited in the NCBI GEO (http://www.ncbi.nlm.nih.gov/geo/) data under the accession number GSE75288.

Comparisons between sets of DPMP-responsive genes and sets of genes responding to other stimuli were done using Venny 2.1, (http://bioinfogp.cnb.csic.es/tools/venny/). The respective gene sets represent all genes that were reported in the cited publications to be significantly up- or –down regulated.

## Additional Information

**How to cite this article**: Bektas, Y. *et al.* The Synthetic Elicitor DPMP (2,4-dichloro-6-{(E)-[(3-methoxyphenyl)imino]methyl}phenol) Triggers Strong Immunity in *Arabidopsis thaliana* and Tomato. *Sci. Rep.* **6**, 29554; doi: 10.1038/srep29554 (2016).

## Supplementary Material

Supplementary Table S1

Supplementary Figures

## Figures and Tables

**Table 1:** Set of Arabidopsis genes significantly differentially expressed in response to DPMP treatment in plate-grown Col-0 seedlings and the set of SUPER-ACID genes.

**Gene Set**	**Number of genes in set**	Enriched GO terms* (with p values)
DPMP-shoots-up	1061	response to stress (p = 1.452e-128);
		response to abiotic or biotic stimulus (p = 1.170e-95);
		signal transduction (p = 1.446e-86);
		transport (p = 3.892e-39);
		other biological processes (p = 3.822e-39)
DPMP-shoots-down	303	electron transport or energy pathways (p = 1.540e-36);
		other metabolic processes (p = 1.037e-11);
		transcription,DNA-dependent (p = 3.319e-08);
		response to stress (p = 9.890e-03);
		abiotic or biotic stimulus (p = 7.598e-03);
		other cellular processes (p = 8.862e-07);
		transport (p = 0.016)
DPMP-roots-up	207	response to stress (p = 1.269e-12);
		response to abiotic or biotic stimulus (p = 1.147e-11);
		signal transduction (p = 1.415e-05);
		transport (p = 2.139e-03);
		other metabolic processes (p = 9.875e-03)
DPMP-roots-down	351	other metabolic processes (p = 1.989e-03);
		response to stress (p = 7.419e-03);
		abiotic or biotic stimulus (p = 7.598e-03);
		other cellular processes (p = 8.862e-07);
		transport (p = 4.095e-03)
*SUPER-ACID*s	75	response to stress (p = 2.313e-31);
		response to abiotic or biotic stimulus (p = 1.742e-27);
		signal transduction (p = 7.759e-22);
		transport (p = 5.216e-12)
