# Supplementary material for: The Synthetic Elicitor DPMP (2,4-dichloro-6-{(E)-[(3-methoxyphenyl)imino]methyl}phenol) Triggers Strong Immunity in Arabidopsis thaliana and Tomato
Source: Sci Rep. 2016 Jul 14;6:29554. doi: 10.1038/srep29554 (PMC4944173; doi:10.1038/srep29554)
Supplement: Supplementary Table S1 [file srep29554-s1.pdf]

The Synthetic Elicitor DPMP (2,4-dichloro-6-{(E)-[(3-methoxyphenyl)imino]methyl}phenol) Trigg
Immunity in Arabidopsis thaliana and Tomato

Yasemin Bektas 1, 2, @ , Melinda Rodriguez-Salus 1, 2, 3, Mercedes Schroeder 1, 2, 3, Adilen
Isgouhi Kaloshian 1, 4 , Thomas Eulgem 1, 2, 3, *

Genes that exhibit in shoots significantly upregulated transcript levels after 3 uM concentration of DPMP (DPMP-shoots_up)

AgI	Desc	logFC	FDR
At2g29110	glutamate receptor 2.8	-10.57272708	4.94E-10
At3g60470	Plant protein of unknown function (DUF247)	-9.837515054	2.82E-12
At5g52740	Copper transport protein family	-9.702639274	8.12E-12
At3g57240	beta-1,3-glucanase 3	-9.592406673	2.74E-07
At4g23320	:eine-rich RLK (RECEPTOR-like protein kinase	-9.455541717	4.56E-06
At3g49340	Cysteine proteinases superfamily protein	-9.26325619	2.92E-06
At2g39030	NA	-9.152658014	0.000117
At5g22570	WRKY DNA-binding protein 38	-9.013485372	1.69E-20
At5g22530		-8.768125127	5.24E-07
At5g07780	tin-binding FH2 (formin homology 2) family prot	-8.699447171	9.24E-10
At4g09770	TRAF-like family protein	-8.685386379	4.24E-09
At1g47980		-8.662264427	3.60E-08
At1g66960	Terpenoid cyclases family protein	-8.447787177	9.45E-09
At4g14390	Ankyrin repeat family protein	-8.278732651	8.32E-09
At5g45180	Flavin-binding monooxygenase family protein	-8.278310074	1.84E-05
At5g22380	NAC domain containing protein 90	-8.12806046	6.79E-09
At2g19190	FLG22-induced receptor-like kinase 1	-8.073102285	3.01E-05
At1g33840	Protein of unknown function (DUF567)	-7.908535232	2.30E-05
At1g75040	pathogenesis-related gene 5	-7.445579424	2.72E-05
At1g65484		-7.366051066	0.002286
At4g10500	ɘ (2OG) and Fe(II)-dependent oxygenase supeı	-7.315018254	2.88E-11
At4g23140	;teine-rich RLK (RECEPTOR-like protein kinase	-7.311488179	9.18E-06
At3g47050	Glycosyl hydrolase family protein	-7.15352214	0.000208
At5g52720	Copper transport protein family	-7.1390585	0.000331
At5g22560	Plant protein of unknown function (DUF247)	-7.105103559	0.000103
At3g44326	F-box family protein	-7.091869645	0.000434
At2g14610	pathogenesis-related gene 1	-7.048034936	6.16E-12
At5g01900	WRKY DNA-binding protein 62	-6.97262381	1.21E-11
At1g10417		-6.85970662	0.000199
At3g48080	alpha/beta-Hydrolases superfamily protein	-6.675814691	2.73E-19
At5g38250	Protein kinase family protein	-6.668043211	8.22E-08
At1g58225		-6.655811842	8.17E-09
At4g23310	:eine-rich RLK (RECEPTOR-like protein kinase	-6.618625741	5.48E-07
At3g57260	beta-1,3-glucanase 2	-6.607145532	0.00055

At4g25000	alpha-amylase-like	-6.602270361	7.79E-15
At4g35180	LYS/HIS transporter 7	-6.572147973	8.51E-09
At1g14880	PLANT CADMIUM RESISTANCE 1	-6.558437024	0.000981
At4g39675		-6.543904857	0.011127
At1g13470	Protein of unknown function (DUF1262)	-6.480915152	4.39E-05
At2g32680	NA	-6.469389333	1.06E-08
At4g11000	Ankyrin repeat family protein	-6.426682729	1.60E-09
At3g56500	serine-rich protein-related	-6.410980534	0.00397
At4g16350	calcineurin B-like protein 6	-6.405731391	0.001534
At4g23150	steine-rich RLK (RECEPTOR-like protein kinase	-6.400614544	6.26E-06
At4g15270	glucosyltransferase-related	-6.399150273	0.006284
At1g67000	Protein kinase superfamily protein	-6.376171008	1.33E-07
At5g10760	Eukaryotic aspartyl protease family protein	-6.360179774	2.08E-05
At3g45130	lanosterol synthase 1	-6.340641912	8.12E-13
At4g13420	high affinity K+ transporter 5	-6.340620938	0.006852
At2g14560	Protein of unknown function (DUF567)	-6.338397303	4.98E-07
At2g43140	NA	-6.285797557	1.36E-07
At3g57460	catalytics;metal ion binding	-6.213849584	2.17E-06
At5g47850	CRINKLY4 related 4	-6.174898707	3.60E-08
At5g60900	receptor-like protein kinase 1	-6.172827491	3.30E-08
At5g54610	ankyrin	-6.164865311	8.29E-09
At3g24900	NA	-6.135611886	4.90E-09
At5g16900	Leucine-rich repeat protein kinase family protei	-6.135011203	0.005991
At5g35940	Mannose-binding lectin superfamily protein	-6.063656218	0.009034
At5g03350	Legume lectin family protein	-6.043138438	2.83E-10
At4g25110	metacaspase 2	-6.017487757	1.15E-05
At5g07040	RING/U-box superfamily protein	-5.971825255	0.01388
At2g47190	NA	-5.950047529	6.24E-09
At4g13890	osphate (PLP)-dependent transferases superfa	-5.910096693	1.09E-08
At4g39830	Cupredoxin superfamily protein	-5.846571005	9.97E-12
At1g51800	Leucine-rich repeat protein kinase family protei	-5.84484164	1.84E-10
At3g23120	NA	-5.842904221	4.94E-10
At2g04495		-5.837153698	9.92E-07
At1g35710	kinase family protein with leucine-rich repeat c	-5.836837358	4.79E-10
At4g16600	tide-diphospho-sugar transferases superfamily	-5.83290211	0.017824
At4g36950	itogen-activated protein kinase kinase kinase 2	-5.776542812	0.043466
At4g28520	cruciferin 3	-5.768293317	0.001317
At2g34940	NA	-5.763246149	1.46E-10
At4g18250	receptor serine/threonine kinase, putative	-5.756705614	6.16E-08
At2g43570	NA	-5.753538893	6.86E-17
At5g52390	PAR1 protein	-5.752188398	2.82E-12
At3g01760	NA	-5.746142697	0.037855
At1g56240	phloem protein 2-B13	-5.737256314	0.027175
At3g07195	NA	-5.733742287	6.16E-09
At3g09960	NA	-5.727953407	0.020794

At5g40990	GDSL lipase 1	-5.72789469	0.024942
At5g44585		-5.650712849	0.00162
At5g24900	:hrome P450, family 714, subfamily A, polypept	-5.63589767	0.029333
At1g70170	matrix metalloproteinase	-5.632185787	0.030369
At1g51790	.eucine-rich repeat protein kinase family proteir	-5.609217024	4.04E-05
At5g10380	RING/U-box superfamily protein	-5.60893967	1.94E-18
At1g09080	Heat shock protein 70 (Hsp 70) family protein	-5.56909688	5.28E-05
At2g18660	plant natriuretic peptide A	-5.565065496	7.09E-05
At1g02450	NIM1-interacting 1	-5.556220584	1.30E-10
At3g28580	NA	-5.551795979	4.29E-06
At3g18715	NA	-5.525267819	0.046476
At4g11890	Protein kinase superfamily protein	-5.518999217	7.03E-05
At5g48657	defense protein-related	-5.518343223	4.58E-10
At1g73805	Calmodulin binding protein-like	-5.509210242	2.08E-08
At4g12500	r/lipid-transfer protein/seed storage 2S albumin	-5.506346445	2.74E-05
At5g09290	Inositol monophosphatase family protein	-5.455210256	9.59E-13
At3g11010	NA	-5.452409628	1.48E-11
At1g02230	NAC domain containing protein 4	-5.420332754	3.03E-13
At5g26690	/y metal transport/detoxification superfamily prc	-5.401131964	4.54E-06
At1g10340	Ankyrin repeat family protein	-5.376910885	4.05E-07
At1g28480	Thioredoxin superfamily protein	-5.365995247	3.15E-07
At5g44568		-5.360724287	1.78E-07
At5g64810	WRKY DNA-binding protein 51	-5.3492074	1.71E-09
At5g24530	ɜ (2OG) and Fe(II)-dependent oxygenase supeı	-5.314512312	6.86E-17
At1g04600	myosin XI A	-5.300622465	1.52E-12
At5g66640	DA1-related protein 3	-5.294485689	1.38E-07
At2g29460	glutathione S-transferase tau 4	-5.269613964	1.53E-10
At3g28890	NA	-5.254888781	9.63E-15
At4g00700	lipid-binding plant phosphoribosyltransferase fa	-5.246223524	1.49E-05
At1g51860	.eucine-rich repeat protein kinase family proteir	-5.223403243	9.16E-09
At4g20000	VQ motif-containing protein	-5.157910472	1.84E-06
At1g23840		-5.111343423	4.34E-10
At3g61280	lopsis thaliana protein of unknown function (DL	-5.102951826	2.73E-10
At1g67980	caffeoyl-CoA 3-O-methyltransferase	-5.070484108	0.006979
At5g24110	WRKY DNA-binding protein 30	-5.018721691	5.83E-06
At5g27060	receptor like protein 53	-5.009417673	3.55E-08
At1g65790	receptor kinase 1	-5.008886534	4.34E-10
At2g47130	NA	-5.005307436	8.78E-09
At2g27660	Cysteine/Histidine-rich C1 domain family proteir	-4.961084022	9.19E-13
At3g25010	NA	-4.952497097	1.67E-14
At5g52760	Copper transport protein family	-4.933474645	0.003084
At1g66700	nethionine-dependent methyltransferases supe	-4.931408141	2.80E-06
At3g26210	NA	-4.930818129	2.50E-08
At5g45000	ɜase resistance protein (TIR-NBS-LRR class) fa	-4.894954051	1.86E-07
At5g57010	calmodulin-binding family protein	-4.86309265	9.40E-09

At5g42830	HXXXD-type acyl-transferase family protein	-4.861728386	1.81E-13
At4g34380	'ransducin/WD40 repeat-like superfamily protei	-4.850828006	5.23E-07
At4g21840	methionine sulfoxide reductase B8	-4.809636151	5.09E-07
At1g09932	Phosphoglycerate mutase family protein	-4.797933883	2.23E-11
At1g58420	Jncharacterised conserved protein UCP03127	-4.772443757	3.62E-06
At2g20142	-Interleukin-Resistance (TIR) domain family pro	-4.766821466	2.07E-07
At4g23220	:eine-rich RLK (RECEPTOR-like protein kinase	-4.765867236	0.000163
At1g73810	:hing beta-1,6-N-acetylglucosaminyltransferase	-4.765201282	2.60E-09
At1g02930	glutathione S-transferase 6	-4.745197494	1.36E-07
At4g14365	XB3 ortholog 4 in Arabidopsis thaliana	-4.743272974	3.22E-07
At1g24140	Matrixin family protein	-4.727635379	3.90E-06
At3g46280	protein kinase-related	-4.72412792	7.73E-07
At5g55460	r/lipid-transfer protein/seed storage 2S albumin	-4.711730299	3.38E-11
At1g24147		-4.686294165	2.13E-06
At3g60140	Glycosyl hydrolase superfamily protein	-4.682039468	0.00072
At5g08760		-4.679920203	3.62E-10
At3g28540	NA	-4.641598367	0.000439
At3g12220	NA	-4.638743333	1.97E-07
At5g59670	.eucine-rich repeat protein kinase family protei	-4.627420312	1.78E-11
At5g25910	receptor like protein 52	-4.623946362	1.23E-06
At5g60950	COBRA-like protein 5 precursor	-4.622358516	2.42E-13
At2g37820	NA	-4.613472118	0.002034
At1g51850	.eucine-rich repeat protein kinase family protei	-4.5837119	6.52E-07
At4g23230	:eine-rich RLK (RECEPTOR-like protein kinase	-4.575960239	1.41E-08
At2g33020	NA	-4.571531065	9.22E-07
At4g38560	dopsis phospholipase-like protein (PEARLI 4) f	-4.561972121	6.89E-07
At1g72060	serine-type endopeptidase inhibitors	-4.554561603	1.92E-08
At1g26200	1 and CLN8 (TLC) lipid-sensing domain contai	-4.552583207	2.50E-05
At2g40750	NA	-4.549287697	4.40E-16
At1g21250	cell wall-associated kinase	-4.540624709	1.33E-05
At3g01080	NA	-4.532755457	8.78E-09
At4g25845		-4.531573039	0.000423
At5g55170	small ubiquitin-like modifier 3	-4.525339777	1.80E-07
At5g55450	r/lipid-transfer protein/seed storage 2S albumin	-4.522157529	2.82E-12
At3g57270	beta-1,3-glucanase 1	-4.511269379	0.000138
At3g45860	steine-rich RLK (RECEPTOR-like protein kinase	-4.502707753	6.10E-08
At2g15390	fucosyltransferase 4	-4.501594718	1.26E-08
At5g35660	Glycine-rich protein family	-4.495410876	2.91E-05
At1g76960		-4.494847472	2.81E-07
At5g52750	y metal transport/detoxification superfamily pro	-4.485178798	6.90E-09
At2g02930	glutathione S-transferase F3	-4.474853395	4.56E-06
At4g27220	ARC domain-containing disease resistance pro	-4.467811194	3.70E-06
At3g25510	NA	-4.447048336	1.17E-08
At3g22231	NA	-4.439300843	1.24E-05
At1g53340	Cysteine/Histidine-rich C1 domain family protei	-4.420716313	0.000173

At3g22235	NA	-4.391497288	0.001693
At5g25250	'PHB domain-containing membrane-associated	-4.378342768	0.000153
At3g01290	NA	-4.375913715	7.67E-05
At1g66880	Protein kinase superfamily protein	-4.366276861	7.16E-10
At5g53110	RING/U-box superfamily protein	-4.36224396	4.34E-10
At2g31880	NA	-4.360159236	1.99E-12
At4g23160	steine-rich RLK (RECEPTOR-like protein kinase	-4.358214445	2.92E-05
At5g11920	6-&1-fructan exohydrolase	-4.358095942	2.23E-08
At3g23010	NA	-4.356805443	3.25E-07
At1g26970	Protein kinase superfamily protein	-4.332813312	0.000189
At1g01560	MAP kinase 11	-4.319480282	2.61E-05
At5g39670	Calcium-binding EF-hand family protein	-4.305593391	1.42E-06
At5g22540	Plant protein of unknown function (DUF247)	-4.305584702	3.94E-06
At4g02520	glutathione S-transferase PHI 2	-4.280502376	2.66E-06
At3g10320	NA	-4.253149486	0.000517
At1g26420	FAD-binding Berberine family protein	-4.24630693	0.000173
At3g28510	NA	-4.245493383	0.00046
At4g23130	steine-rich RLK (RECEPTOR-like protein kinase	-4.243139903	4.16E-09
At3g23510	NA	-4.221632319	5.35E-06
At3g21320	NA	-4.202501222	8.58E-05
At3g50470	homolog of RPW8 3	-4.200950674	8.32E-09
At2g44380	NA	-4.187222768	8.55E-06
At3g47210	Plant protein of unknown function (DUF247)	-4.184260196	0.001108
At2g46430	NA	-4.173510775	2.50E-08
At1g20350	translocase inner membrane subunit 17-1	-4.158010338	2.88E-06
At1g72070	Chaperone DnaJ-domain superfamily protein	-4.157078416	1.54E-07
At2g25510		-4.143502903	9.22E-07
At1g69720	heme oxygenase 3	-4.138032929	1.43E-07
At4g14640	calmodulin 8	-4.137777558	0.001183
At1g47890	receptor like protein 7	-4.134093108	0.000105
At2g21850	Cysteine/Histidine-rich C1 domain family protein	-4.133678783	1.67E-16
At2g26390	Serine protease inhibitor (SERPIN) family protei	-4.13030076	0.002234
At1g13830	rbohydrate-binding X8 domain superfamily prot	-4.11484192	1.44E-05
At1g30900	VACUOLAR SORTING RECEPTOR 6	-4.111236411	2.42E-13
At4g03450	Ankyrin repeat family protein	-4.102863402	0.001001
At1g07620	GTP-binding protein Obg/CgtA	-4.098570389	6.75E-07
At5g13080	WRKY DNA-binding protein 75	-4.086539332	1.51E-07
At2g17040	NAC domain containing protein 36	-4.083887434	8.04E-06
At1g02920	glutathione S-transferase 7	-4.083206386	1.73E-11
At3g62780	n-dependent lipid-binding (CaLB domain) family	-4.071812026	3.01E-06
At4g25070		-4.064062864	1.39E-09
At4g17660	Protein kinase superfamily protein	-4.052907702	0.002933
At4g00960	Protein kinase superfamily protein	-4.052165605	7.66E-05
At4g12490	r/lipid-transfer protein/seed storage 2S albumin	-4.051115191	0.002298
At3g56710	sigma factor binding protein 1	-4.04272176	1.32E-10

At3g53150	UDP-glucosyl transferase 73D1	-4.021276858	6.99E-06
At4g33355	r/lipid-transfer protein/seed storage 2S albumin	-3.998156332	0.001638
At4g14400	ankyrin repeat family protein	-3.96722783	1.01E-05
At3g47090	_eucine-rich repeat protein kinase family protei	-3.966172823	2.51E-06
At1g65510		-3.962583094	3.60E-08
At5g45380	sodium symporters;urea transmembrane trans	-3.961041301	3.03E-09
At2g26560	phospholipase A 2A	-3.957226678	4.14E-05
At3g60540	protein translocase Sec, Sec61-beta subunit pr	-3.949365145	9.44E-07
At1g35230	arabinogalactan protein 5	-3.937852561	0.039006
At2g24850	tyrosine aminotransferase 3	-3.919804822	0.009401
At1g24145		-3.912811603	4.14E-06
At1g03850	Glutaredoxin family protein	-3.892247928	4.34E-08
At3g56400	WRKY DNA-binding protein 70	-3.870145262	1.78E-11
At4g23610	enesis abundant (LEA) hydroxyproline-rich glyc	-3.864634336	3.09E-08
At3g50200	Plant protein of unknown function (DUF247)	-3.852208217	0.007348
At2g39210	NA	-3.840573927	3.25E-09
At1g58390	ease resistance protein (CC-NBS-LRR class) fa	-3.828371921	3.40E-05
At1g26390	FAD-binding Berberine family protein	-3.815274366	0.031844
At1g09930	oligopeptide transporter 2	-3.802129151	0.029826
At5g08240		-3.79322251	2.48E-08
At3g47480	Calcium-binding EF-hand family protein	-3.786827935	0.000657
At5g59680	_eucine-rich repeat protein kinase family protei	-3.782390433	3.87E-08
At3g23110	NA	-3.766077753	4.33E-08
At5g44820	cleotide-diphospho-sugar transferase family pro	-3.758056682	1.39E-09
At5g18470	urculin-like (mannose-binding) lectin family prot	-3.746687556	2.60E-09
At5g64000	Inositol monophosphatase family protein	-3.74461789	1.53E-07
At3g52430	alpha/beta-Hydrolases superfamily protein	-3.74013878	8.39E-13
At5g54720	Ankyrin repeat family protein	-3.7380412	7.28E-05
At3g01513	NA	-3.723202421	1.66E-05
At3g48630		-3.71998612	0.001706
At2g26400	acireductone dioxygenase 3	-3.714544561	2.32E-07
At2g41100	NA	-3.709095521	2.51E-07
At5g41160	purine permease 12	-3.70899249	7.88E-07
At3g25882	NA	-3.701752719	3.46E-11
At1g05675	UDP-Glycosyltransferase superfamily protein	-3.688007595	0.016385
At4g31230	protein with adenine nucleotide alpha hydrolas	-3.676209192	0.001742
At1g34420	rich repeat transmembrane protein kinase famil	-3.67446551	1.71E-09
At5g22520		-3.645886453	0.002677
At3g11340	NA	-3.622334732	0.000193
At5g25440	Protein kinase superfamily protein	-3.621538284	8.88E-07
At4g04972		-3.618446994	0.027533
At1g66920	Protein kinase superfamily protein	-3.612398946	0.000244
At2g39200	NA	-3.61143164	0.00047
At2g24600	Ankyrin repeat family protein	-3.611085382	1.18E-06
At1g31580	ECS1	-3.610825869	2.00E-05

At1g08450	calreticulin 3	-3.609245445	1.30E-10
At2g46440	NA	-3.608772813	6.89E-07
At1g33720	chrome P450, family 76, subfamily C, polypepti	-3.606953816	0.000125
At2g18690		-3.602378328	3.46E-06
At5g46050	peptide transporter 3	-3.581279951	6.22E-08
At3g13850	NA	-3.578548639	0.001074
At1g14870	PLANT CADMIUM RESISTANCE 2	-3.575883804	3.86E-08
At3g50480	homolog of RPW8 4	-3.563663646	1.03E-07
At2g33580	NA	-3.560132494	1.48E-11
At2g22860	phytosulfokine 2 precursor	-3.533030448	0.000414
At1g02360	Chitinase family protein	-3.52856769	1.29E-07
At1g07000	exocyst subunit exo70 family protein B2	-3.52640483	6.24E-09
At3g18485	NA	-3.523828472	6.77E-07
At2g31865	NA	-3.518668592	7.37E-08
At3g09940	NA	-3.514780336	1.42E-05
At5g41750	ase resistance protein (TIR-NBS-LRR class) fa	-3.512149352	0.000369
At4g30640	RNI-like superfamily protein	-3.50468555	3.64E-06
At5g54710	Ankyrin repeat family protein	-3.502269759	5.91E-05
At5g19240	Glycoprotein membrane precursor GPI-anchore	-3.497494995	4.13E-07
At5g67450	zinc-finger protein 1	-3.489336453	0.000513
At3g51440	m-dependent phosphotriesterase superfamily p	-3.48364374	1.38E-09
At2g25000	WRKY DNA-binding protein 60	-3.482357448	3.23E-07
At3g23240	NA	-3.478518976	0.023509
At5g65090	DNAse I-like superfamily protein	-3.475915977	0.016556
At1g23830		-3.472894933	1.69E-08
At3g07520	NA	-3.432736501	3.43E-15
At5g09470	dicarboxylate carrier 3	-3.430925781	0.003707
At4g02330	invertase/pectin methylesterase inhibitor super	-3.422291847	1.33E-06
At2g26440	invertase/pectin methylesterase inhibitor super	-3.416020014	2.53E-08
At2g15490	UDP-glycosyltransferase 73B4	-3.402198457	0.003233
At4g21380	receptor kinase 3	-3.40145132	4.52E-05
At3g46770	AP2/B3-like transcriptional factor family protein	-3.39956005	8.20E-05
At5g18350	ase resistance protein (TIR-NBS-LRR class) fa	-3.393291048	0.003542
At3g13437	NA	-3.383378371	1.13E-05
At4g00955		-3.375108234	9.45E-09
At5g11940	Subtilase family protein	-3.372063957	0.00463
At5g61010	exocyst subunit exo70 family protein E2	-3.368608644	4.65E-08
At5g42530		-3.366580358	0.001407
At5g44460	calmodulin like 43	-3.358863656	0.001256
At5g39580	Peroxidase superfamily protein	-3.350120845	0.004106
At2g43000	NA	-3.332089931	1.06E-06
At3g08870	NA	-3.331614526	1.84E-05
At1g32960	Subtilase family protein	-3.329497034	0.000329
At1g21240	wall associated kinase 3	-3.32834389	0.016044
At1g71890	Major facilitator superfamily protein	-3.320671586	0.000126

At5g49680	ation protein domain ;RNA pol II promoter Fmp	-3.316585708	9.72E-15
At3g21150	NA	-3.315398316	0.007176
At1g21520		-3.311627914	0.000344
At4g23700	cation/H+ exchanger 17	-3.302961529	5.35E-05
At1g57630	-Interleukin-Resistance (TIR) domain family pro	-3.292242229	0.001095
At3g04070	NA	-3.286503805	4.32E-05
At5g11930	Thioredoxin superfamily protein	-3.281432158	0.000274
At4g11521	eceptor-like protein kinase-related family protei	-3.27756364	0.000861
At4g21120	amino acid transporter 1	-3.273202808	1.96E-07
At1g65690	enesis abundant (LEA) hydroxyproline-rich glyc	-3.261081467	4.98E-07
At2g30140	UDP-Glycosyltransferase superfamily protein	-3.25843673	9.67E-09
At3g04220	NA	-3.258050854	0.000469
At3g51330	Eukaryotic aspartyl protease family protein	-3.256220363	1.59E-10
At5g02780	glutathione transferase lambda 1	-3.250178676	0.001507
At5g50200	nitrate transmembrane transporters	-3.246202883	8.76E-08
At1g01340	cyclic nucleotide gated channel 10	-3.238376318	1.69E-08
At3g29034	NA	-3.235818543	1.42E-05
At5g54490	pinoid-binding protein 1	-3.224200094	6.15E-07
At4g30270	xyloglucan endotransglucosylase/hydrolase 24	-3.220979689	3.46E-05
At1g34180	NAC domain containing protein 16	-3.220198685	5.24E-07
At2g25440	receptor like protein 20	-3.212603472	0.007267
At3g50930	cytochrome BC1 synthesis	-3.207835472	3.25E-09
At2g29350	senescence-associated gene 13	-3.202019876	0.000181
At5g56230	prenylated RAB acceptor 1.G2	-3.193718731	3.17E-05
At4g20110	VACUOLAR SORTING RECEPTOR 7	-3.186547713	2.00E-10
At2g29120	glutamate receptor 2.7	-3.179487549	1.32E-10
At3g29250	NA	-3.173729855	0.00132
At5g05460	Glycosyl hydrolase family 85	-3.168129058	1.54E-07
At4g39580	alactose oxidase/kelch repeat superfamily prote	-3.167579443	8.49E-05
At4g14370	ase resistance protein (TIR-NBS-LRR class) fa	-3.153557622	2.72E-05
At5g27420	carbon/nitrogen insensitive 1	-3.149973421	0.000173
At5g62770	Protein of unknown function (DUF1645)	-3.143429078	0.000357
At5g43420	RING/U-box superfamily protein	-3.1414642	6.22E-08
At1g35210		-3.139266169	0.000101
At1g56550	noGalacturonan speciﬁc Xylosyltransfe	-3.131336796	1.16E-08
At1g56120	eucine-rich repeat transmembrane protein kinas	-3.129988105	3.25E-09
At3g09010	NA	-3.111877031	1.19E-08
At4g21903	MATE efflux family protein	-3.086632744	3.09E-07
At5g05320	AD/NAD(P)-binding oxidoreductase family prote	-3.077443162	9.18E-08
At2g32030	NA	-3.075090228	4.29E-06
At4g04490	teine-rich RLK (RECEPTOR-like protein kinase	-3.073571214	1.46E-05
At1g53100	ching beta-1,6-N-acetylglucosaminyltransferase	-3.063830172	4.56E-06
At1g76970	Target of Myb protein 1	-3.054992892	1.18E-12
At2g33070	NA	-3.051028522	0.035799
At2g36780	NA	-3.0481507	0.000261

At4g34135	UDP-glucosyltransferase 73B2	-3.045731759	4.14E-07
At4g08850	e-rich repeat receptor-like protein kinase family	-3.038099799	8.64E-09
At4g37370	chrome P450, family 81, subfamily D, polypepti	-3.028779172	0.001414
At3g17690	NA	-3.026174433	0.0009
At5g16170	ching beta-1,6-N-acetylglucosaminyltransferase	-3.024681181	1.87E-06
At2g45220	NA	-3.023207593	0.001523
At5g60800	y metal transport/detoxification superfamily prc	-3.019332235	0.017782
At4g31800	WRKY DNA-binding protein 18	-3.018948297	3.13E-05
At5g07770	Actin-binding FH2 protein	-3.011178248	3.62E-10
At3g50800		-3.002353883	0.000785
At5g24200	alpha/beta-Hydrolases superfamily protein	-3.000215015	0.000393
At3g48090	alpha/beta-Hydrolases superfamily protein	-2.998409642	2.87E-12
At3g25020	NA	-2.966158507	6.22E-08
At1g08050	nc finger (C3HC4-type RING finger) family prote	-2.964994131	6.73E-05
At5g49690	UDP-Glycosyltransferase superfamily protein	-2.96112378	1.58E-05
At1g24150	formin homologue 4	-2.954431195	4.08E-09
At5g38210	Protein kinase family protein	-2.948108753	4.09E-07
At4g33050	calmodulin-binding family protein	-2.93966404	7.41E-09
At2g38860	NA	-2.939026281	2.22E-05
At1g72240		-2.936987133	0.001097
At5g37600	glutamine synthase clone R1	-2.935871474	5.90E-09
At1g51620	Protein kinase superfamily protein	-2.933610047	0.039751
At5g18780	F-box/RNI-like superfamily protein	-2.930318691	1.95E-07
At4g01750	rhamnogalacturonan xylosyltransferase 2	-2.929628569	0.000111
At3g13950	NA	-2.924531025	0.000111
At5g57550	xyloglucan endotransglucosylase/hydrolase 25	-2.920537358	0.041282
At1g72280	endoplasmic reticulum oxidoreductins 1	-2.91994644	3.10E-10
At1g72930	toll/interleukin-1 receptor-like	-2.919460747	2.33E-08
At3g15240	NA	-2.918508639	0.004955
At4g28390	ADP/ATP carrier 3	-2.916199838	4.71E-05
At2g32160	NA	-2.911956508	0.000192
At4g23170	eceptor-like protein kinase-related family protei	-2.911430087	3.26E-05
At3g09490	NA	-2.897591573	5.13E-08
At4g26120	peat family protein / BTB/POZ domain-containi	-2.896800856	3.59E-05
At5g25930	n kinase family protein with leucine-rich repeat c	-2.896306693	1.02E-10
At1g34750	Protein phosphatase 2C family protein	-2.892537802	1.63E-07
At1g04540	n-dependent lipid-binding (CaLB domain) family	-2.887727317	7.10E-08
At5g24655	response to low sulfur 4	-2.887643922	0.000672
At2g21510	AJ heat shock N-terminal domain-containing prc	-2.887549258	0.009777
At1g65500		-2.885171319	2.89E-05
At5g24210	alpha/beta-Hydrolases superfamily protein	-2.881075184	2.40E-08
At5g64120	Peroxidase superfamily protein	-2.867119477	0.000135
At2g44290	NA	-2.861817461	4.26E-08
At3g44350	NAC domain containing protein 61	-2.857286918	0.0392
At5g67340	ARM repeat superfamily protein	-2.847166886	2.28E-09

At1g33610	Leucine-rich repeat (LRR) family protein	-2.844741799	2.88E-08
At5g22890	C2H2 and C2HC zinc fingers superfamily protei	-2.841850392	0.001211
At5g39020	alectin/receptor-like protein kinase family prote	-2.840388535	6.22E-08
At3g22160	NA	-2.838235618	5.12E-07
At1g30720	FAD-binding Berberine family protein	-2.83499545	0.000201
At1g78410	VQ motif-containing protein	-2.831509777	0.000382
At1g55910	zinc transporter 11 precursor	-2.828038241	8.14E-07
At1g17600	ease resistance protein (TIR-NBS-LRR class) fa	-2.826998328	9.58E-06
At3g22930	NA	-2.826033672	0.001293
At3g61190	BON association protein 1	-2.82475817	0.000277
At3g44400	ease resistance protein (TIR-NBS-LRR class) fa	-2.811539485	5.07E-07
At1g72910	terleukin-Resistance (TIR) domain-containing p	-2.806810003	3.13E-05
At5g20400	e (2OG) and Fe(II)-dependent oxygenase super	-2.80623402	8.89E-08
At2g15042	Leucine-rich repeat (LRR) family protein	-2.800780576	0.001962
At3g10600	NA	-2.7906891	0.003959
At1g59590	ZCF37	-2.78679423	4.39E-05
At3g48850	phosphate transporter 3;2	-2.785228886	0.000662
At1g56540	ease resistance protein (TIR-NBS-LRR class) fa	-2.773022898	1.82E-05
At3g14280	NA	-2.771775283	0.000439
At3g20600	NA	-2.766713982	5.99E-07
At3g21720	NA	-2.762529922	0.034033
At3g26500	NA	-2.751788718	1.24E-06
At3g22060	NA	-2.748051229	2.65E-06
At1g45145	thioredoxin H-type 5	-2.744302529	2.49E-05
At1g80840	WRKY DNA-binding protein 40	-2.738054296	1.16E-05
At1g30730	FAD-binding Berberine family protein	-2.730177968	0.007675
At3g11080	NA	-2.726024102	0.000164
At3g44300	nitrilase 2	-2.723164074	0.000981
At5g60780	nitrate transporter 2.3	-2.722844741	0.025069
At2g42350	NA	-2.722730424	0.001198
At2g26190	calmodulin-binding family protein	-2.722538149	8.60E-08
At5g57220	chrome P450, family 81, subfamily F, polypepti	-2.699204554	0.002215
At2g30250	WRKY DNA-binding protein 25	-2.688716842	9.64E-06
At1g65486		-2.683310353	0.000447
At4g23240	teine-rich RLK (RECEPTOR-like protein kinase	-2.680898291	7.26E-05
At2g34655	NA	-2.66517905	0.001245
At3g54150	nethionine-dependent methyltransferases supe	-2.663017338	7.66E-05
At2g41090	NA	-2.652355153	0.00167
At1g17610	Disease resistance protein (TIR-NBS class)	-2.651084144	9.85E-05
At4g23810	WRKY family transcription factor	-2.645287341	1.82E-08
At1g65845		-2.645116527	0.001234
At3g25780	NA	-2.640996276	0.002031
At3g29000	NA	-2.640702386	0.014547
At3g21780	NA	-2.631847291	0.012277
At2g04450	nudix hydrolase homolog 6	-2.626502431	1.10E-09

At1g52200	PLAC8 family protein	-2.626095151	1.59E-05
At4g37010	centrin 2	-2.622092228	0.026608
At1g80120	Protein of unknown function (DUF567)	-2.619831298	0.006891
At4g36150	ease resistance protein (TIR-NBS-LRR class) fa	-2.60901255	1.84E-05
At5g52810	AD(P)-binding Rossmann-fold superfamily prote	-2.598596246	1.26E-05
At3g08970	NA	-2.598184589	0.000672
At2g30750	NA	-2.595353365	7.64E-05
At1g18390	Protein kinase superfamily protein	-2.594811208	1.19E-05
At3g20960	NA	-2.58771623	3.60E-08
At4g38550	dopsis phospholipase-like protein (PEARLI 4) f	-2.587203642	0.000465
At5g38900	Thioredoxin superfamily protein	-2.572568658	6.18E-05
At5g40780	lysine histidine transporter 1	-2.568625093	2.63E-05
At2g46400	NA	-2.565700801	1.44E-05
At3g15540	NA	-2.565120184	6.69E-06
At1g33030	O-methyltransferase family protein	-2.561484282	0.002923
At1g12160	Flavin-binding monooxygenase family protein	-2.559046019	7.69E-06
At1g26380	FAD-binding Berberine family protein	-2.557111657	0.037922
At1g17745	D-3-phosphoglycerate dehydrogenase	-2.552941249	0.013559
At1g31540	ease resistance protein (TIR-NBS-LRR class) fa	-2.549007029	1.51E-06
At4g29520		-2.545410041	1.94E-07
At1g15790		-2.542025276	2.52E-07
At5g16770	myb domain protein 9	-2.541739112	0.000513
At1g13750	Purple acid phosphatases superfamily protein	-2.540863372	1.10E-07
At2g46940	NA	-2.533061855	0.011026
At5g22550	Plant protein of unknown function (DUF247)	-2.530477309	0.028451
At3g28340	NA	-2.528980219	0.000197
At2g47800	NA	-2.525806443	0.000261
At3g54960	PDI-like 1-3	-2.524010436	0.002747
At5g58940	modulin-binding receptor-like cytoplasmic kinas	-2.522117775	1.46E-08
At5g07760	y 2 domain-containing protein / FH2 domain-co	-2.511741461	6.65E-06
At1g77510	PDI-like 1-2	-2.510238736	3.62E-10
At1g17744		-2.50553993	0.011618
At5g24510	60S acidic ribosomal protein family	-2.495771996	0.015136
At4g22305	alpha/beta-Hydrolases superfamily protein	-2.486626704	0.015907
At4g01740	Cysteine/Histidine-rich C1 domain family protein	-2.483789404	1.56E-05
At4g13820	Leucine-rich repeat (LRR) family protein	-2.48364446	0.001097
At2g31020	NA	-2.474313916	6.63E-05
At2g44460	NA	-2.469494417	0.001619
At5g43910	pfkB-like carbohydrate kinase family protein	-2.465806052	1.09E-08
At3g09830	NA	-2.465774263	3.60E-08
At3g60420	Phosphoglycerate mutase family protein	-2.465053352	0.000194
At5g47220	ethylene responsive element binding factor 2	-2.458869399	0.000175
At5g41740	ease resistance protein (TIR-NBS-LRR class) fa	-2.456326087	0.009257
At3g46690	UDP-Glycosyltransferase superfamily protein	-2.455680733	0.040335
At4g22485	r/lipid-transfer protein/seed storage 2S albumin	-2.45559737	0.043129

At5g58500	Protein of unknown function (DUF640)	-2.454545732	0.006999
At4g22530	nethionine-dependent methyltransferases supe	-2.436321282	0.000151
At3g26170	NA	-2.434837159	5.84E-08
At1g18570	myb domain protein 51	-2.433639236	9.08E-07
At4g01920	Cysteine/Histidine-rich C1 domain family protein	-2.428845785	5.75E-05
At3g26220	NA	-2.426780018	3.03E-05
At2g21840	Cysteine/Histidine-rich C1 domain family protein	-2.420166162	0.044465
At4g14746		-2.418907952	6.54E-06
At5g40690		-2.418255799	1.09E-05
At5g03360	DC1 domain-containing protein	-2.415511142	4.37E-05
At1g51890	Leucine-rich repeat protein kinase family protein	-2.414661432	0.0005
At1g21270	wall-associated kinase 2	-2.408462527	0.00073
At5g48540	eceptor-like protein kinase-related family protei	-2.406431791	0.009858
At2g35000	NA	-2.405777749	0.02406
At1g71910		-2.405742248	7.11E-06
At5g47960	RAB GTPase homolog A4C	-2.405340822	0.008851
At1g50180	ARC domain-containing disease resistance prc	-2.396589347	0.00216
At5g17760	ning nucleoside triphosphate hydrolases super	-2.393986026	0.001523
At5g42440	Protein kinase superfamily protein	-2.39113315	1.33E-05
At4g21490	NAD(P)H dehydrogenase B3	-2.389346328	0.029657
At4g28490	cine-rich receptor-like protein kinase family pro	-2.387467588	4.37E-05
At4g02420	ıcanavalin A-like lectin protein kinase family prc	-2.385327936	0.000207
At2g35980	NA	-2.379624168	0.022094
At4g37530	Peroxidase superfamily protein	-2.373661607	1.61E-05
At4g27740	Yippee family putative zinc-binding protein	-2.373643421	0.000228
At2g02810	UDP-galactose transporter 1	-2.36439331	1.02E-08
At5g64510		-2.352477263	0.000646
At5g26920	Cam-binding protein 60-like G	-2.348959904	0.004693
At4g21926		-2.347959242	0.037675
At3g62600	DNAJ heat shock family protein	-2.345079246	7.54E-05
At1g67330	Protein of unknown function (DUF579)	-2.341149944	4.85E-08
At3g04720	NA	-2.339593567	0.0005
At3g13080	NA	-2.33861205	7.14E-08
At3g21330	NA	-2.337060734	0.01617
At3g59700	lectin-receptor kinase	-2.333740181	0.00051
At4g08470	MAPK/ERK kinase kinase 3	-2.324157342	0.000181
At2g19130	S-locus lectin protein kinase family protein	-2.321885393	1.62E-09
At4g21850	methionine sulfoxide reductase B9	-2.314317215	9.96E-07
At5g66630	DA1-related protein 5	-2.312687647	2.69E-06
At2g32140	NA	-2.311052393	0.004718
At1g67970	heat shock transcription factor A8	-2.30795336	6.28E-09
At4g38830	teine-rich RLK (RECEPTOR-like protein kinase	-2.307503623	0.024191
At2g23680	Cold acclimation protein WCOR413 family	-2.304322453	0.001035
At3g14620	NA	-2.303193684	8.58E-05
At1g52290	Protein kinase superfamily protein	-2.297491984	0.003213

At2g37710	NA	-2.29625104	3.13E-05
At3g47220	phosphatidylinositol-speciwc phospholipase C9	-2.290024191	0.034033
At3g51860	cation exchanger 3	-2.28891006	0.001982
At1g02220	NAC domain containing protein 3	-2.285919756	0.003037
At1g74440	Protein of unknown function (DUF962)	-2.284480517	0.000417
At5g12880	proline-rich family protein	-2.284042051	0.028754
At1g49000		-2.282548542	0.037242
At4g27480	ching beta-1,6-N-acetylglucosaminyltransferase	-2.280047726	0.000152
At1g76040	calcium-dependent protein kinase 29	-2.27794444	3.64E-08
At1g21310	extensin 3	-2.273480589	0.000828
At4g11850	phospholipase D gamma 1	-2.271921249	2.49E-07
At3g52400	syntaxin of plants 122	-2.268395102	0.00729
At1g15040	l glutamine amidotransferase-like superfamily p	-2.266763642	0.001848
At4g37150	methyl esterase 9	-2.266605027	0.001096
At2g13790	somatic embryogenesis receptor-like kinase 4	-2.265357781	0.000403
At5g38340	ase resistance protein (TIR-NBS-LRR class) fa	-2.254308555	0.000593
At3g04210	NA	-2.248171655	0.016233
At3g60580	C2H2-like zinc finger protein	-2.239581735	1.24E-05
At4g24026		-2.231258115	0.011618
At1g55210	esistance-responsive (dirigent-like protein) fam	-2.224281824	4.14E-05
At1g69810	WRKY DNA-binding protein 36	-2.213777816	0.014196
At1g70990	proline-rich family protein	-2.208879705	0.010313
At1g67810	sulfur E2	-2.205803777	0.00367
At4g16960	ase resistance protein (TIR-NBS-LRR class) fa	-2.196894131	1.75E-05
At1g69520	nethionine-dependent methyltransferases supe	-2.187493592	0.002183
At1g16260	Wall-associated kinase family protein	-2.184742222	0.000481
At5g46260	ase resistance protein (TIR-NBS-LRR class) fa	-2.180491396	7.77E-05
At3g09020	NA	-2.171311283	0.00023
At1g27730	salt tolerance zinc finger	-2.168900049	0.002508
At4g23180	teine-rich RLK (RECEPTOR-like protein kinase	-2.164733587	8.25E-06
At5g46520	ase resistance protein (TIR-NBS-LRR class) fa	-2.160850756	0.000405
At1g14260	RING/FYVE/PHD zinc finger superfamily protein	-2.159927356	2.26E-05
At2g16870	ase resistance protein (TIR-NBS-LRR class) fa	-2.159383467	1.35E-05
At1g05575		-2.159360516	0.010484
At3g26230	NA	-2.158130717	0.000662
At1g04980	PDI-like 2-2	-2.150144918	3.73E-06
At3g11840	NA	-2.146325397	0.001049
At1g25400		-2.144888855	0.016227
At3g13090	NA	-2.139263365	0.013996
At5g25820	Exostosin family protein	-2.13842818	0.000217
At3g26470	NA	-2.138040902	0.000169
At5g26220	ChaC-like family protein	-2.136634217	0.007176
At3g50140	Plant protein of unknown function (DUF247)	-2.133118672	0.038068
At4g26070	MAP kinase/ ERK kinase 1	-2.108485375	1.26E-05
At4g01700	Chitinase family protein	-2.108355432	1.43E-07

At1g49050	Eukaryotic aspartyl protease family protein	-2.107976024	0.00014
At2g41410	NA	-2.106842035	0.011618
At5g59580	UDP-glucosyl transferase 76E1	-2.106550328	0.04181
At1g09560	germin-like protein 5	-2.104023945	1.02E-05
At2g39710	NA	-2.096691763	3.71E-06
At5g37540	Eukaryotic aspartyl protease family protein	-2.094818663	5.82E-06
At5g54860	Major facilitator superfamily protein	-2.093704675	5.61E-06
At1g79450	ALA-interacting subunit 5	-2.092124219	0.005177
At3g50950	HOPZ-ACTIVATED RESISTANCE 1	-2.090961083	9.92E-06
At5g08790	Meristem) domain transcriptional regulator sup	-2.086494644	3.18E-09
At1g76980		-2.084777205	2.49E-05
At3g11000	NA	-2.082829613	0.000608
At1g17430	alpha/beta-Hydrolases superfamily protein	-2.081153019	4.97E-05
At4g01720	WRKY family transcription factor	-2.077860534	9.22E-07
At4g05020	NAD(P)H dehydrogenase B2	-2.074018062	4.24E-06
At1g72920	-Interleukin-Resistance (TIR) domain family pro	-2.069828697	0.036548
At4g33300	ADR1-like 1	-2.069476641	7.68E-07
At5g48380	BAK1-interacting receptor-like kinase 1	-2.069153309	5.77E-06
At3g28940	NA	-2.067986759	0.000331
At1g14370	protein kinase 2A	-2.06259777	3.71E-06
At1g10990		-2.058067164	0.000182
At3g57700	Protein kinase superfamily protein	-2.044314195	0.000646
At3g25610	NA	-2.042862147	1.20E-06
At3g11820	NA	-2.042148252	0.000156
At3g55700	UDP-Glycosyltransferase superfamily protein	-2.040139579	0.0084
At1g11330	S-locus lectin protein kinase family protein	-2.039543727	3.73E-05
At4g12480	r/lipid-transfer protein/seed storage 2S albumin	-2.03579787	0.018132
At2g22500	uncoupling protein 5	-2.033421684	3.45E-08
At5g18310		-2.032158704	0.020081
At3g51920	calmodulin 9	-2.030561541	0.000332
At2g17120	lysm domain GPI-anchored protein 2 precursor	-2.030441103	0.000446
At5g20230	blue-copper-binding protein	-2.024295435	0.0493
At4g15975	RING/U-box superfamily protein	-2.020972821	0.020159
At1g30700	FAD-binding Berberine family protein	-2.009911915	0.016671
At2g05380	glycine-rich protein 3 short isoform	-2.006758892	0.012703
At5g46230	Protein of unknown function, DUF538	-2.004920744	0.015287
At1g67800	Calcium-dependent phospholipid-binding protei	-2.002614177	0.000135
At3g11640	NA	-1.998423791	0.011127
At1g01010	NAC domain containing protein 1	-1.997682569	0.00384
At4g23030	MATE efflux family protein	-1.996588756	0.001363
At4g23470	PLAC8 family protein	-1.99176991	0.019743
At1g48320	Thioesterase superfamily protein	-1.99051131	0.000121
At4g34150	ı-dependent lipid-binding (CaLB domain) family	-1.98515432	0.000731
At4g02410	ıcanavalin A-like lectin protein kinase family pro	-1.979407893	0.004187
At3g24090	NA	-1.977344137	9.96E-07

At5g07010	sulfotransferase 2A	-1.973692882	0.000554
At1g74360	.eucine-rich repeat protein kinase family proteir	-1.97219407	6.80E-05
At1g68570	Major facilitator superfamily protein	-1.970145533	1.01E-05
At3g05650	NA	-1.9701088	0.000608
At4g15610	Uncharacterised protein family (UPF0497)	-1.965816488	0.006864
At4g19660	NPR1-like protein 4	-1.965745637	3.07E-05
At1g50520	hrome P450, family 705, subfamily A, polypepti	-1.964931208	0.009937
At2g15760	Protein of unknown function (DUF1645)	-1.961144143	0.017214
At5g48850	atricopeptide repeat (TPR)-like superfamily pro	-1.960372173	0.002954
At1g69730	Wall-associated kinase family protein	-1.957497589	0.002351
At4g39890	RAB GTPase homolog H1C	-1.957111919	0.011972
At5g61790	calnexin 1	-1.950553472	0.00594
At1g13260	related to ABI3/VP1 1	-1.942118399	0.000763
At5g44582		-1.941289784	0.006339
At5g53370	pectin methylesterase PCR fragment F	-1.936117804	7.30E-06
At1g55450	nethionine-dependent methyltransferases supe	-1.930906867	2.73E-05
At1g72900	terleukin-Resistance (TIR) domain-containing p	-1.929206442	0.000128
At5g57480	ning nucleoside triphosphate hydrolases super	-1.926270026	0.019209
At3g51430	m-dependent phosphotriesterase superfamily p	-1.924996645	1.69E-05
At3g49120	peroxidase CB	-1.92275446	4.14E-05
At2g15480	UDP-glucosyl transferase 73B5	-1.922279985	0.000419
At5g49520	WRKY DNA-binding protein 48	-1.920084577	0.004772
At1g58190	receptor like protein 9	-1.916675355	0.012241
At4g04570	:eine-rich RLK (RECEPTOR-like protein kinase	-1.912567262	1.54E-05
At4g11300	Protein of unknown function (DUF793)	-1.907615059	0.007304
At2g43150	NA	-1.905126315	0.010159
At3g59880		-1.904526437	0.010207
At1g52770	Phototropic-responsive NPH3 family protein	-1.903067529	0.001375
At4g35110	dopsis phospholipase-like protein (PEARLI 4) f	-1.899993535	0.019276
At3g08720	NA	-1.899463999	6.05E-05
At3g51890	Clathrin light chain protein	-1.895915294	2.91E-05
At3g44720	arogenate dehydratase 4	-1.893950172	0.000129
At2g24570	WRKY DNA-binding protein 17	-1.889239985	0.000248
At3g16990	NA	-1.887680362	0.019009
At1g13210	autoinhibited Ca2+/ATPase II	-1.885827758	0.000209
At4g04695	calcium-dependent protein kinase 31	-1.88139736	0.000157
At5g12890	UDP-Glycosyltransferase superfamily protein	-1.874401059	0.004168
At3g54640	tryptophan synthase alpha chain	-1.870520154	4.24E-07
At3g13790	NA	-1.863511279	0.000418
At5g42020	Heat shock protein 70 (Hsp 70) family protein	-1.863172486	0.021168
At1g75750	GAST1 protein homolog 1	-1.86207564	0.028212
At4g04220	receptor like protein 46	-1.860923071	0.00059
At4g31500	chrome P450, family 83, subfamily B, polypepti	-1.857307676	0.017087
At4g24190	Chaperone protein htpG family protein	-1.856425203	0.000347
At4g32870	cyclase/dehydrase and lipid transport superfar	-1.856173193	0.006923

At2g16595	inslocon-associated protein (TRAP), alpha subu	-1.84793674	0.000116
At4g16260	Glycosyl hydrolase superfamily protein	-1.846100014	0.045437
At3g09790	NA	-1.842617804	0.035808
At1g67920		-1.840281802	0.002779
At2g38470	NA	-1.8394379	0.029144
At1g65490		-1.839217307	0.008956
At3g52710		-1.838597413	7.13E-06
At1g21750	PDI-like 1-1	-1.832572188	0.000163
At1g71040	Cupredoxin superfamily protein	-1.831392867	1.38E-06
At1g71880	sucrose-proton symporter 1	-1.830235609	0.000674
At4g21940	calcium-dependent protein kinase 15	-1.829824067	0.028451
At4g17670	Protein of unknown function (DUF581)	-1.829429671	0.004205
At1g13990		-1.825526922	0.010808
At1g52800	e (2OG) and Fe(II)-dependent oxygenase super	-1.825177987	0.000793
At1g10050	family 10 protein / carbohydrate-binding domain	-1.817287068	0.006681
At2g43620	NA	-1.816982376	0.010183
At3g51340	Eukaryotic aspartyl protease family protein	-1.813593522	0.049058
At3g17700	NA	-1.81334502	0.00066
At3g16510	NA	-1.806883417	0.003848
At4g34131	UDP-glucosyl transferase 73B3	-1.803839127	0.002218
At1g22990	y metal transport/detoxification superfamily pro	-1.80117598	0.012022
At1g43910	ning nucleoside triphosphate hydrolases super	-1.796993253	1.24E-05
At1g49750	Leucine-rich repeat (LRR) family protein	-1.795923936	3.48E-05
At5g23850	lopsis thaliana protein of unknown function (DU	-1.795719182	7.77E-05
At3g44260	otidyl transferase, ribonuclease H-like superfam	-1.792991142	0.004814
At5g40230	dulin MtN21 /EamA-like transporter family prote	-1.792064346	0.032231
At1g63860	ease resistance protein (TIR-NBS-LRR class) fa	-1.786056022	0.002155
At3g28930	NA	-1.783057918	0.000369
At4g23450	RING/U-box superfamily protein	-1.777291835	0.01513
At2g30550	NA	-1.771486907	2.33E-05
At3g57550	guanylate kinase	-1.768865535	0.006315
At2g40270	NA	-1.765681627	6.38E-07
At4g27280	Calcium-binding EF-hand family protein	-1.765630997	7.60E-05
At3g19930	NA	-1.765255293	0.012886
At4g13510	ammonium transporter 1;1	-1.764096771	0.043151
At3g47010	Glycosyl hydrolase family protein	-1.762872504	9.47E-05
At1g63350	ease resistance protein (CC-NBS-LRR class) fa	-1.756856982	0.009311
At2g30930	NA	-1.756843239	0.001706
At2g40095	NA	-1.7564191	0.001706
At1g51270	molecules;transmembrane receptors;structural	-1.754081738	0.033233
At5g14930	senescence-associated gene 101	-1.747858422	4.37E-05
At2g23170	Auxin-responsive GH3 family protein	-1.74349356	0.042895
At3g26830	NA	-1.741686357	0.045516
At4g16660	heat shock protein 70 (Hsp 70) family protein	-1.736448916	2.34E-05
At2g17220	Protein kinase superfamily protein	-1.73507083	3.43E-06

At2g45510	NA	-1.731096129	1.42E-05
At3g45640	mitogen-activated protein kinase 3	-1.726025701	4.49E-05
At1g19960		-1.722975472	0.002981
At2g27920	serine carboxypeptidase-like 51	-1.720651343	0.001363
At4g24970	ıse-, DNA gyrase B-, and HSP90-like ATPase f	-1.720198703	0.002666
At1g27330	₹ibosome associated membrane protein RAMP	-1.713043864	0.003631
At4g01010	cyclic nucleotide-gated channel 13	-1.712379887	0.001827
At1g05680	Uridine diphosphate glycosyltransferase 74E2	-1.705267348	0.021981
At3g16030	NA	-1.703976725	0.001245
At2g28940	Protein kinase superfamily protein	-1.703746153	0.012039
At1g65040	RING/U-box superfamily protein	-1.703565416	0.003496
At2g23200	Protein kinase superfamily protein	-1.701496969	3.94E-05
At3g26600	NA	-1.700621477	7.48E-07
At4g23880		-1.697136821	0.004547
At5g02290	Protein kinase superfamily protein	-1.696618048	6.98E-06
At4g24920	secE/sec61-gamma protein transport protein	-1.693252761	0.013317
At4g17500	ethylene responsive element binding factor 1	-1.691420235	0.041881
At5g41400	RING/U-box superfamily protein	-1.685437744	0.001355
At1g03210	Phenazine biosynthesis PhzC/PhzF protein	-1.683430489	0.000195
At1g16670	Protein kinase superfamily protein	-1.681731298	0.000209
At3g13380	NA	-1.678090366	7.22E-06
At1g16110	wall associated kinase-like 6	-1.677583776	0.001735
At1g20510	OPC-8:0 CoA ligase1	-1.676663109	0.000209
At2g18680		-1.670791916	0.035592
At5g19250	ɜlycoprotein membrane precursor GPI-anchore	-1.668290901	0.000723
At5g45510	Leucine-rich repeat (LRR) family protein	-1.659492758	0.001432
At4g12720	MutT/nudix family protein	-1.657553619	0.000588
At4g37640	calcium ATPase 2	-1.656357456	0.000338
At1g11125		-1.650235971	0.009311
At1g55265	Protein of unknown function, DUF538	-1.646522705	0.00055
At3g13560	NA	-1.646020662	2.65E-05
At3g50280	HXXXD-type acyl-transferase family protein	-1.64557789	0.041196
At4g13810	receptor like protein 47	-1.639623557	0.01554
At3g47380	ɛrtase/pectin methylesterase inhibitor superfam	-1.638486473	0.018486
At1g04530	atricopeptide repeat (TPR)-like superfamily pro	-1.632494798	0.004037
At4g27260	Auxin-responsive GH3 family protein	-1.627437454	0.000182
At4g02380	senescence-associated gene 21	-1.627264329	0.004154
At4g11530	:eine-rich RLK (RECEPTOR-like protein kinase	-1.626237804	0.001529
At5g61250	glucuronidase 1	-1.62588345	0.000182
At1g52780	Protein of unknown function (DUF2921)	-1.622386846	2.80E-06
At3g50260	ɔperatively regulated by ethylene and jasmonat	-1.617667401	0.020794
At5g10740	Protein phosphatase 2C family protein	-1.61095105	0.001258
At5g45500	RNI-like superfamily protein	-1.607132905	0.000331
At1g12420	ACT domain repeat 8	-1.607069836	1.80E-05
At5g44070	phytochelatin synthase 1 (PCS1)	-1.603794007	0.000152

At1g09210	calreticulin 1b	-1.600151252	0.000425
At1g30410	multidrug resistance-associated protein 13	-1.598931265	0.002159
At2g47000	NA	-1.597049712	0.007857
At2g31890	NA	-1.593059284	1.31E-05
At4g17490	ethylene responsive element binding factor 6	-1.592692318	0.023013
At2g42360	NA	-1.59060153	0.006852
At5g52870		-1.589682717	0.000135
At1g63720		-1.583232567	0.001613
At4g26060	Ribosomal protein L18ae family	-1.582554534	0.000152
At5g40720	Domain of unknown function (DUF23)	-1.578561367	0.002121
At5g14330		-1.575228834	0.033284
At5g26170	WRKY DNA-binding protein 50	-1.574585061	0.047749
At2g41940	NA	-1.568507212	0.007741
At2g46150	NA	-1.564699387	0.006917
At1g74200	receptor like protein 16	-1.563416635	0.020159
At3g16720	NA	-1.562448799	0.000146
At4g10970		-1.562048077	0.034495
At1g30420	multidrug resistance-associated protein 12	-1.562039638	0.001884
At4g00330	modulin-binding receptor-like cytoplasmic kinas	-1.558242425	0.015823
At1g74100	sulfotransferase 16	-1.557165542	0.000672
At1g03400	(2OG) and Fe(II)-dependent oxygenase supe	-1.555660596	0.03428
At3g57330	autoinhibited Ca2+-ATPase 11	-1.553722413	0.004975
At3g55980	salt-inducible zinc finger 1	-1.551487182	0.000423
At3g45620	ransducin/WD40 repeat-like superfamily protei	-1.550544446	7.35E-05
At1g66970	SHV3-like 2	-1.548981653	0.007176
At5g25190	ntegrase-type DNA-binding superfamily protei	-1.548326951	0.008017
At2g46600	NA	-1.545944126	0.000598
At3g45290	Seven transmembrane MLO family protein	-1.542266905	0.003496
At4g26270	phosphofructokinase 3	-1.537134028	1.63E-05
At4g00240	phospholipase D beta 2	-1.536494395	0.003199
At1g62840	Protein of unknown function (DUF1442)	-1.535424509	0.005983
At5g61070	one deacetylase of the RPD3/HDA1 superfamil	-1.525586272	0.008956
At1g50740	Transmembrane proteins 14C	-1.524960755	0.004218
At1g77660	H3 K4-specific methyltransferase SET7/9 famil	-1.524165651	0.000588
At1g65800	receptor kinase 2	-1.523084855	0.019637
At5g11970	Protein of unknown function (DUF3511)	-1.517738373	0.000259
At2g28160	FER-like regulator of iron uptake	-1.51701168	0.029183
At3g43800	glutathione S-transferase tau 27	-1.516906427	0.012838
At2g02350	SKP1 interacting partner 3	-1.515964048	0.0493
At1g74300	alpha/beta-Hydrolases superfamily protein	-1.515349887	0.000893
At1g06000	UDP-Glycosyltransferase superfamily protein	-1.513425433	0.041818
At4g29110		-1.513376346	0.006923
At5g27920	F-box family protein	-1.508476695	0.017153
At4g23250	kinases;protein kinases	-1.505488046	0.000151
At4g23260	teine-rich RLK (RECEPTOR-like protein kinase	-1.498704196	0.017782

At1g31130		-1.496549754	0.01183
At4g34390	extra-large GTP-binding protein 2	-1.496329534	0.000248
At5g36220	cytochrome p450 81d1	-1.494435739	0.025752
At5g45730	Cysteine/Histidine-rich C1 domain family protei	-1.491962293	0.041282
At5g25450	Cytochrome bd ubiquinol oxidase, 14kDa subun	-1.491760024	0.048741
At3g45730		-1.489202528	0.045294
At4g18880	heat shock transcription factor A4A	-1.488541832	0.000296
At1g08830	copper/zinc superoxide dismutase 1	-1.484649381	0.020247
At4g25940	ENTH/ANTH/VHS superfamily protein	-1.48256622	0.008989
At3g44630	ease resistance protein (TIR-NBS-LRR class) fa	-1.48048157	0.014468
At2g23770	ly protein / peptidoglycan-binding LysM domai	-1.478389427	0.018679
At5g53550	YELLOW STRIPE like 3	-1.478303743	0.016648
At3g28480	NA	-1.477645184	0.000217
At5g66210	calcium-dependent protein kinase 28	-1.461462867	0.003774
At5g15860	prenylcysteine methylesterase	-1.460559238	0.006339
At1g77765		-1.459642562	0.044509
At5g39050	HXXXD-type acyl-transferase family protein	-1.455765954	0.000193
At1g75170	14p-like phosphatidylinositol transfer family pro	-1.455429735	0.00073
At3g19010	NA	-1.453996999	0.000127
At5g55560	Protein kinase superfamily protein	-1.447918427	0.041282
At2g43850	NA	-1.442788747	0.005022
At1g22280	tochrome-associated protein phosphatase type	-1.441628986	0.001579
At5g45110	NPR1-like protein 3	-1.440769783	0.001328
At4g27470	RING membrane-anchor 3	-1.440656071	0.000761
At4g36990	heat shock factor 4	-1.436226106	0.001996
At4g36988	nserved peptide upstream open reading frame	-1.436226086	0.001996
At1g65250	Protein kinase superfamily protein	-1.432742896	0.044287
At1g28660	DSL-like Lipase/Acylhydrolase superfamily prot	-1.428004972	0.028238
At5g36930	ease resistance protein (TIR-NBS-LRR class) fa	-1.427925922	0.000522
At1g74210	PLC-like phosphodiesterases superfamily protei	-1.42608102	0.011165
At3g57480	zinc finger (C2H2 type, AN1-like) family protein	-1.425250779	0.017087
At2g25110	tromal cell-derived factor 2-like protein precurs	-1.424511393	0.000205
At5g02760	Protein phosphatase 2C family protein	-1.424352852	0.006381
At5g01850	Protein kinase superfamily protein	-1.4230693	0.009719
At5g04930	aminophospholipid ATPase 1	-1.420609099	0.000261
At3g45260	C2H2-like zinc finger protein	-1.414632152	0.013452
At5g06300	Putative lysine decarboxylase family protein	-1.410393391	0.013914
At3g07580	NA	-1.409151468	0.027187
At4g24040	trehalase 1	-1.408976771	0.016419
At1g25370	Protein of unknown function (DUF1639)	-1.408310051	0.009257
At3g47780	ABC2 homolog 6	-1.402395376	0.003635
At5g46510	ease resistance protein (TIR-NBS-LRR class) fa	-1.401494803	0.017884
At4g05590		-1.400358882	0.022318
At5g66910	ease resistance protein (CC-NBS-LRR class) fa	-1.39933052	0.004106
At1g30755	Protein of unknown function (DUF668)	-1.399050186	0.010313

At1g20780	senescence-associated E3 ubiquitin ligase 1	-1.396340203	0.022221
At1g27770	autoinhibited Ca2+-ATPase 1	-1.39392093	0.000195
At2g01650	plant UBX domain-containing protein 2	-1.393079057	0.000355
At4g37520	Peroxidase superfamily protein	-1.392886908	0.005306
At3g20250	NA	-1.392695507	0.002446
At1g33560	ease resistance protein (CC-NBS-LRR class) fa	-1.391885671	0.000522
At4g28720	Flavin-binding monooxygenase family protein	-1.387065033	0.029826
At1g68690	Protein kinase superfamily protein	-1.386058428	0.001386
At5g03160	homolog of mamallian P58IPK	-1.378025869	0.000562
At4g02660	/BEACH domain ;WD domain, G-beta repeat p	-1.374551147	0.002693
At1g19180	jasmonate-zim-domain protein 1	-1.373819914	0.020247
At1g07220	lopsis thaliana protein of unknown function (DU	-1.373222316	0.006917
At2g29090	chrome P450, family 707, subfamily A, polypept	-1.370059397	0.022491
At1g21050	Protein of unknown function, DUF617	-1.368633567	0.044467
At1g03370	ium/lipid-binding and GRAM domain containing	-1.367255103	0.002933
At1g18910	zinc ion binding;zinc ion binding	-1.36664597	0.000175
At2g28630	3-ketoacyl-CoA synthase 12	-1.36651756	0.013996
At5g05090	Homeodomain-like superfamily protein	-1.366346566	0.00039
At4g23270	teine-rich RLK (RECEPTOR-like protein kinase	-1.365987756	0.018055
At5g24520	Transducin/WD40 repeat-like superfamily protei	-1.365682873	0.012308
At1g79380	lependent phospholipid-binding protein (Copine	-1.364359541	0.015407
At3g47570	Leucine-rich repeat protein kinase family protein	-1.363177988	0.019571
At5g61210	e N-ethylmaleimide-sensitive factor adaptor pro	-1.3604772	0.001576
At1g17620	enesis abundant (LEA) hydroxyproline-rich glyc	-1.359544793	0.015682
At3g54810	fic GATA-type zinc finger transcription factor fa	-1.355806267	0.028231
At1g20490	IP-dependent synthetase and ligase family prot	-1.353585257	0.027552
At5g10695		-1.35356348	0.015957
At3g11650	NA	-1.351710688	0.016227
At3g50650	GRAS family transcription factor	-1.344345752	0.011165
At5g38990	alectin/receptor-like protein kinase family prote	-1.339652228	0.022262
At5g55860	Plant protein of unknown function (DUF827)	-1.33416797	0.028102
At1g72330	alanine aminotransferase 2	-1.333719114	0.000362
At2g46620	NA	-1.332836247	0.001513
At5g52900		-1.329811591	0.020081
At3g49370	um-dependent protein kinase (CDPK) family pr	-1.325240905	0.011441
At2g28890	poltergeist like 4	-1.323321999	0.000155
At2g42950	NA	-1.323247485	0.011066
At4g23010	UDP-galactose transporter 2	-1.319217929	0.011229
At3g63080	glutathione peroxidase 5	-1.316426375	0.003448
At2g38790	NA	-1.312630839	0.017307
At1g14360	UDP-galactose transporter 3	-1.311948613	0.00083
At2g20610	Tyrosine transaminase family protein	-1.31046036	0.009937
At3g15760	NA	-1.305603996	0.03456
At1g59660	Nucleoporin autopeptidase	-1.30545115	0.00033
At4g13180	AD(P)-binding Rossmann-fold superfamily prote	-1.304956386	0.024865

At1g05570	callose synthase 1	-1.302712328	0.00132
At5g10770	Eukaryotic aspartyl protease family protein	-1.298774635	0.015407
At5g25790	esmin/TSO1-like CXC domain-containing prote	-1.294458138	0.031598
At3g26180	NA	-1.293429197	0.006022
At3g05630	NA	-1.293243924	0.032873
At3g25600	NA	-1.284117671	0.038729
At4g14220	RING-H2 group F1A	-1.284006678	0.005096
At4g27070	tryptophan synthase beta-subunit 2	-1.283459874	0.00212
At1g73500	MAP kinase kinase 9	-1.280071995	0.012961
At4g37900	rotein of unknown function (duplicated DUF139	-1.278494888	0.036904
At1g09970	cine-rich receptor-like protein kinase family pro	-1.274690128	0.020159
At1g61560	Seven transmembrane MLO family protein	-1.273726822	0.047292
At3g61630	cytokinin response factor 6	-1.273303174	0.017884
At4g37560	Acetamidase/Formamidase family protein	-1.271182263	0.035257
At1g62422		-1.270421892	0.01584
At1g26690	emp24/gp25L/p24 family/GOLD family protein	-1.269997946	0.001386
At1g42990	basic region/leucine zipper motif 60	-1.268015188	0.016175
At5g05190	Protein of unknown function (DUF3133)	-1.264175797	0.005306
At1g59710	Protein of unknown function (DUF569)	-1.261908291	0.043926
At4g16780	homeobox protein 2	-1.261903668	0.002483
At4g36090	doreductase, 2OG-Fe(II) oxygenase family prot	-1.26040424	0.007176
At2g33530	NA	-1.258405075	0.009075
At3g45650	nitrate excretion transporter1	-1.25813509	0.017061
At1g21900	emp24/gp25L/p24 family/GOLD family protein	-1.257878057	0.035081
At2g04400	Aldolase-type TIM barrel family protein	-1.254053086	0.001167
At2g40600	NA	-1.25226182	0.002723
At1g06620	e (2OG) and Fe(II)-dependent oxygenase supei	-1.251564078	0.021828
At5g52540	Protein of unknown function (DUF819)	-1.245124343	0.001432
At5g45470	Protein of unknown function (DUF594)	-1.244626577	0.041195
At1g56510	Disease resistance protein (TIR-NBS-LRR class	-1.238641599	0.036927
At1g28280	VQ motif-containing protein	-1.23497155	0.045927
At5g11000	Plant protein of unknown function (DUF868)	-1.233940278	0.03272
At3g16700	NA	-1.233432423	0.030618
At5g43100	Eukaryotic aspartyl protease family protein	-1.231279299	0.04181
At3g61670	Protein of unknown function (DUF3133)	-1.22875338	0.001012
At1g18890	calcium-dependent protein kinase 1	-1.225732452	0.001784
At4g30560	cyclic nucleotide gated channel 9	-1.224967496	0.001249
At1g22070	TGA1A-related gene 3	-1.224543226	0.007403
At1g03290		-1.223256602	0.001033
At1g64710	ES-like zinc-binding dehydrogenase family pro	-1.222383957	0.015823
At3g53670		-1.221118261	0.012241
At3g53668	nserved peptide upstream open reading frame	-1.220141864	0.012241
At3g49210	O-acyltransferase (WSD1-like) family protein	-1.218462892	0.0376
At5g49280	hydroxyproline-rich glycoprotein family protein	-1.216794365	0.026137
At1g03430	histidine-containing phosphotransfer factor 5	-1.215746092	0.00826

At1g25220	anthranilate synthase beta subunit 1	-1.21526763	0.022491
At1g70530	steine-rich RLK (RECEPTOR-like protein kinase	-1.214844116	0.004016
At2g29990	alternative NAD(P)H dehydrogenase 2	-1.210980832	0.011064
At5g26340	Major facilitator superfamily protein	-1.209673352	0.007009
At4g39955	alpha/beta-Hydrolases superfamily protein	-1.204532803	0.021844
At3g14840	NA	-1.20397869	0.012974
At1g68470	Exostosin family protein	-1.20287231	0.042219
At2g24390	IG2-like (avirulence induced gene) family prote	-1.201828582	0.01334
At1g69850	nitrate transporter 1:2	-1.201087061	0.002024
At2g40140	NA	-1.200197931	0.012241
At4g22780	ACT domain repeat 7	-1.199497085	0.03333
At5g06860	polygalacturonase inhibiting protein 1	-1.199056783	0.020622
At5g56250	hapless 8	-1.197518751	0.013317
At1g64280	regulatory protein (NPR1)	-1.194092953	0.002651
At5g07340	Calreticulin family protein	-1.190167593	0.017556
At1g70740	Protein kinase superfamily protein	-1.189948187	0.008119
At5g07820	Plant calmodulin-binding protein-related	-1.189727292	0.04681
At1g63750	ease resistance protein (TIR-NBS-LRR class) fa	-1.188912829	0.035081
At1g14790	RNA-dependent RNA polymerase 1	-1.188821979	0.002004
At1g61260	Protein of unknown function (DUF761)	-1.185662049	0.030664
At1g28380	MAC/Perforin domain-containing protein	-1.184802402	0.009388
At5g04720	ADR1-like 2	-1.183976295	0.013692
At2g31990	NA	-1.183531154	0.036515
At3g55950	CRINKLY4 related 3	-1.178078021	0.024401
At2g35810	NA	-1.17593854	0.005806
At5g62070	IQ-domain 23	-1.172193393	0.02161
At3g12360	NA	-1.170250811	0.035799
At5g37740	n-dependent lipid-binding (CaLB domain) family	-1.16893224	0.025615
At1g05630	onuclease/exonuclease/phosphatase family pro	-1.161871068	0.020209
At3g13910	NA	-1.155889358	0.017091
At3g21810	NA	-1.150294982	0.020673
At5g07360	Amidase family protein	-1.150179393	0.002278
At5g08380	alpha-galactosidase 1	-1.149952154	0.025738
At5g63680	Pyruvate kinase family protein	-1.147843896	0.002947
At1g63880	ease resistance protein (TIR-NBS-LRR class) fa	-1.144924343	0.025179
At4g21800	ning nucleoside triphosphate hydrolases super	-1.144756671	0.025193
At5g18490	Plant protein of unknown function (DUF946)	-1.141034732	0.011674
At1g76930	extensin 4	-1.137230407	0.012308
At5g19930	otein of unknown function DUF92, transmembra	-1.135932853	0.010155
At2g20960	dopsis phospholipase-like protein (PEARLI 4) f	-1.131079929	0.040704
At3g44670	ease resistance protein (TIR-NBS-LRR class) fa	-1.130529731	0.024427
At2g24360	Protein kinase superfamily protein	-1.127884705	0.024807
At2g02800	protein kinase 2B	-1.125368944	0.009311
At1g12990	-1,4-N-acetylglucosaminyltransferase family pro	-1.124294432	0.004922
At4g35310	calmodulin-domain protein kinase 5	-1.118655444	0.036248

At5g28830	calcium-binding EF hand family protein	-1.118270074	0.030201
At5g11250	)isease resistance protein (TIR-NBS-LRR class	-1.114625736	0.010155
At3g18370	NA	-1.10933648	0.003574
At4g34480	O-Glycosyl hydrolases family 17 protein	-1.108705987	0.010523
At3g60520		-1.107099983	0.01513
At4g13920	receptor like protein 50	-1.103946281	0.033405
At3g61460	brassinosteroid-responsive RING-H2	-1.098084547	0.047751
At3g27890	NA	-1.097013073	0.006999
At1g32700	PLATZ transcription factor family protein	-1.095892543	0.029175
At5g62630	hipl2 protein precursor	-1.095290931	0.033492
At5g23510		-1.093567192	0.03323
At5g06750	Protein phosphatase 2C family protein	-1.08991112	0.006486
At1g18590	sulfotransferase 17	-1.087016161	0.005409
At5g12930		-1.085764357	0.01616
At5g49570	peptide-N-glycanase 1	-1.08350731	0.01334
At5g15870	glycosyl hydrolase family 81 protein	-1.082774981	0.007306
At3g54200	enesis abundant (LEA) hydroxyproline-rich glyc	-1.081438769	0.032297
At3g04480	NA	-1.081129547	0.012449
At3g10500	NA	-1.078950975	0.004922
At1g74280	alpha/beta-Hydrolases superfamily protein	-1.067633768	0.044509
At3g10640	NA	-1.063176918	0.029743
At1g72790	hydroxyproline-rich glycoprotein family protein	-1.063098131	0.026763
At2g03530	ureide permease 2	-1.062624258	0.017368
At2g17290	Calcium-dependent protein kinase family protei	-1.062568355	0.012002
At5g10610	chrome P450, family 81, subfamily K, polypepti	-1.05688574	0.023475
At5g24810	ABC1 family protein	-1.052448154	0.009005
At2g01670	nudix hydrolase homolog 17	-1.051807409	0.033405
At5g47120	BAX inhibitor 1	-1.048092383	0.020247
At1g22410	Class-II DAHP synthetase family protein	-1.044117711	0.041919
At5g54140	IAA-leucine-resistant (ILR1)-like 3	-1.041479243	0.039155
At3g63030	methyl-CPG-binding domain 4	-1.040526696	0.006735
At4g25840	glycerol-3-phosphatase 1	-1.039657736	0.016419
At4g36210	Protein of unknown function (DUF726)	-1.034458849	0.037088
At1g14170	RNA-binding KH domain-containing protein	-1.030877385	0.037931
At5g46470	ase resistance protein (TIR-NBS-LRR class) fa	-1.029644128	0.020424
At1g22650	Plant neutral invertase family protein	-1.029555522	0.024469
At3g59660	in-containing protein / GRAM domain-containin	-1.028473958	0.048373
At4g26090	ARC domain-containing disease resistance pro	-1.024005688	0.019917
At5g21090	Leucine-rich repeat (LRR) family protein	-1.023043314	0.020794
At4g25900	Galactose mutarotase-like superfamily protein	-1.021525805	0.009441
At2g45010	NA	-1.021366609	0.012449
At1g61250	secretory carrier 3	-1.019813131	0.01513
At3g56410	Protein of unknown function (DUF3133)	-1.017812483	0.019001
At4g20830	FAD-binding Berberine family protein	-1.016613293	0.043607
At1g64610	Transducin/WD40 repeat-like superfamily protei	-1.013089117	0.027175

At4g08230	glycine-rich protein	-1.008177862	0.009442
At2g45500	NA	-1.006303791	0.019068
At4g19670	RING/U-box superfamily protein	-1.005091519	0.010375
At2g19710	julator of Vps4 activity in the MVB pathway pro	-1.004376352	0.039969
At3g28450	NA	-1.002824141	0.015323
At1g51660	mitogen-activated protein kinase kinase 4	-1.002024599	0.025737
At2g44500	NA	-1.001556797	0.027189
At2g44180	NA	-0.998846033	0.025454
At3g07570	NA	-0.997325228	0.012974
At1g15110	phosphatidyl serine synthase family protein	-0.992510429	0.044379
At5g49760	.eucine-rich repeat protein kinase family proteir	-0.984162325	0.031809
At2g40520	NA	-0.982529543	0.044585
At2g32800	NA	-0.976282798	0.046682
At2g29720	\D/NAD(P)-binding oxidoreductase family prote	-0.974937146	0.043052
At2g40950	NA	-0.964541327	0.011229
At1g59960	AD(P)-linked oxidoreductase superfamily prote	-0.964316227	0.031322
At2g17130	isocitrate dehydrogenase subunit 2	-0.959387399	0.037675
At5g57035	ox domain-containing protein kinase family pro	-0.954244734	0.040796
At1g11300	ses;protein kinases;ATP binding;sugar binding;l	-0.95001124	0.026033
At5g25770	alpha/beta-Hydrolases superfamily protein	-0.939145869	0.036773
At1g67940	non-intrinsic ABC protein 3	-0.935504895	0.041282
At4g18930	ase/cyclic nucleotide phosphodiesterase family	-0.923490376	0.048759
At1g70520	iteine-rich RLK (RECEPTOR-like protein kinase	-0.923028147	0.020929
At1g03230	Eukaryotic aspartyl protease family protein	-0.910280415	0.038893
At4g12020	protein kinase family protein	-0.909076091	0.025647
At4g17720	NA-binding (RRM/RBD/RNP motifs) family prote	-0.90828785	0.020069
At1g72700	family protein / haloacid dehalogenase-like hyd	-0.903254451	0.025403
At1g17440	Transcription initiation factor TFIID subunit A	-0.901207196	0.049424
At5g05730	anthranilate synthase alpha subunit 1	-0.894238643	0.046331
At1g54320	id-effect modulator 3) family protein / CDC50 fa	-0.881905175	0.039553
At1g64810	lopsis thaliana protein of unknown function (DU	-0.881732657	0.030315
At1g34300	lectin protein kinase family protein	-0.876415395	0.033868
At1g68710	family protein / haloacid dehalogenase-like hyd	-0.875626744	0.048373
At4g28400	Protein phosphatase 2C family protein	-0.859712428	0.025623
At2g14720	vacuolar sorting receptor 4	-0.83658233	0.027378
At3g24180	NA	-0.829350595	0.043932
At2g45910	NA	-0.814358169	0.048713
Genes that exhibit in shoots significantly downregulated transcript levels after 3 uM concentration of DPMP (DPMP-shoots_down)			
At5g64460	Phosphoglycerate mutase family protein	0.831341659	0.03728
At1g17650	glyoxylate reductase 2	0.877772652	0.02913
At1g18650	plasmodesmata callose-binding protein 3	0.901323342	0.047552
At5g57800	Fatty acid hydroxylase superfamily	0.923994469	0.028231
At1g01790	K+ efflux antiporter 1	0.924651655	0.027053
At5g67260	CYCLIN D3;2	0.942275705	0.028281

At1g32470	Single hybrid motif superfamily protein	0.953757046	0.037538
At3g05800	NA	0.956568107	0.048257
At2g38820	NA	0.960139511	0.022399
At3g15570	NA	0.973642222	0.043376
At4g33000	calcineurin B-like protein 10	0.979714517	0.018562
At1g21790	;1 and CLN8 (TLC) lipid-sensing domain contai	0.979739745	0.037796
At4g25700	beta-hydroxylase 1	0.980179877	0.033284
At3g22150	NA	0.987428665	0.044465
At1g30520	acyl-activating enzyme 14	0.9959215	0.035074
At1g22370	UDP-glucosyl transferase 85A5	1.000366235	0.030201
At1g45474	photosystem I light harvesting complex gene 5	1.001273483	0.033664
At2g39470	NA	1.006456231	0.030201
At1g55810	uridine kinase-like 3	1.010458568	0.048373
At3g09780	NA	1.038079537	0.048713
At5g03190	nserved peptide upstream open reading frame	1.045501181	0.040825
At1g64770	NDH-dependent cyclic electron flow 1	1.046659347	0.048949
At2g20680	Glycosyl hydrolase superfamily protein	1.048584948	0.03958
At1g54740	Protein of unknown function (DUF3049)	1.060490914	0.022795
At5g62670	H(+)-ATPase 11	1.064068271	0.031064
At3g56160	Sodium Bile acid symporter family	1.073929507	0.029719
At2g37790	NA	1.074199989	0.013317
At1g20650	Protein kinase superfamily protein	1.074270693	0.006054
At3g61080	Protein kinase superfamily protein	1.082210443	0.043819
At3g24800	NA	1.086667928	0.008219
At4g19530	ase resistance protein (TIR-NBS-LRR class) fa	1.096678161	0.024401
At3g10940	NA	1.098073089	0.01001
At4g17810	C2H2 and C2HC zinc fingers superfamily protei	1.099948463	0.048497
At1g21070	Nucleotide-sugar transporter family protein	1.112214482	0.016739
At2g22730	Major facilitator superfamily protein	1.112691502	0.033387
At5g55970	RING/U-box superfamily protein	1.125156633	0.032578
At3g54720	Peptidase M28 family protein	1.130432564	0.046274
At1g06360	Fatty acid desaturase family protein	1.134201571	0.021299
At5g10150	Domain of unknown function (DUF966)	1.141135153	0.016127
At5g63780	RING/FYVE/PHD zinc finger superfamily protei	1.14154981	0.020294
At1g18730	NDH dependent flow 6	1.150273621	0.024865
At1g28100		1.151704704	0.019052
At1g54570	Esterase/lipase/thioesterase family protein	1.158086321	0.036927
At5g44410	FAD-binding Berberine family protein	1.160217081	0.04753
At2g46910	NA	1.167464099	0.025069
At1g74780	odulin-like / Major Facilitator Superfamily prote	1.170142836	0.020794
At1g22630		1.170452311	0.039033
At3g15520	NA	1.177029707	0.026495
At4g13770	chrome P450, family 83, subfamily A, polypepti	1.179732558	0.047161
At5g09820	d-lipid associated protein PAP / fibrillin family p	1.189692945	0.045516
At2g18890	Protein kinase superfamily protein	1.19534382	0.006339

At5g17670	alpha/beta-Hydrolases superfamily protein	1.195723874	0.013195
At4g04630	Protein of unknown function, DUF584	1.196403802	0.022778
At5g04360	limit dextrinase	1.199084327	0.00341
At1g64200	vacuolar H+-ATPase subunit E isoform 3	1.20020066	0.008627
At3g25670	NA	1.201423967	0.03759
At5g10920	L-Aspartase-like family protein	1.202328244	0.043927
At5g22920	IY-type/CTCHY-type/RING-type Zinc finger prot	1.204371801	0.046682
At5g57170	Tautomerase/MIF superfamily protein	1.204933378	0.012565
At2g01590	chlororespiratory reduction 3	1.205570396	0.003436
At2g43560	NA	1.207500853	0.035417
At2g18710	SECY homolog 1	1.211876335	0.037978
At3g14810	NA	1.214331165	0.009103
At1g33170	nethionine-dependent methyltransferases supe	1.218287873	0.01007
At2g16750	protein with adenine nucleotide alpha hydrolas	1.220436744	0.02062
At2g38870	NA	1.231743182	0.047413
At1g03090	ıse alpha chain, mitochondrial / 3-methylcroton	1.233488059	0.012961
At5g14410		1.240474855	0.039006
At4g29905		1.243042294	0.047413
At1g64355		1.246422717	0.009289
At5g04770	cationic amino acid transporter 6	1.249846683	0.030369
At1g06080	delta 9 desaturase 1	1.258900428	0.019179
At5g46690	beta HLH protein 71	1.259374157	0.016227
At1g27030		1.265240756	0.004218
At5g55740	atricopeptide repeat (TPR)-like superfamily pro	1.26804827	0.004739
At2g35130	NA	1.269803332	0.006735
At2g38760	NA	1.270904329	0.029859
At2g41340	NA	1.27222582	0.023426
At2g29310	AD(P)-binding Rossmann-fold superfamily prote	1.27302603	0.007951
At3g19710	NA	1.27373258	0.047413
At5g59350		1.275639485	0.017556
At3g62750	beta glucosidase 8	1.277636864	0.001775
At5g52100	Dihydrodipicolinate reductase, bacterial/plant	1.281065018	0.003213
At4g24050	AD(P)-binding Rossmann-fold superfamily prote	1.283742465	0.007176
At1g44740		1.288049354	0.027428
At1g76570	Chlorophyll A-B binding family protein	1.300003638	0.022262
At4g20760	AD(P)-binding Rossmann-fold superfamily prote	1.310250758	0.017583
At5g62430	cycling DOF factor 1	1.311708452	0.043497
At2g37260	NA	1.312408687	0.03503
At5g05860	UDP-glucosyl transferase 76C2	1.315740326	0.001912
At5g65730	xyloglucan endotransglucosylase/hydrolase 6	1.317611998	0.043682
At2g41290	NA	1.328062268	0.038326
At2g01918	PsbQ-like 3	1.332716683	0.031801
At3g55290	AD(P)-binding Rossmann-fold superfamily prote	1.333344863	0.032236
At1g70310	spermidine synthase 2	1.343387108	0.023091
At1g65590	beta-hexosaminidase 3	1.352571304	0.020794

At1g16850		1.352754024	0.02965
At5g23120	ystem II stability/assembly factor, chloroplast (H	1.367161059	0.042219
At1g47580	entatricopeptide repeat (PPR) superfamily prote	1.371184909	0.039129
At2g22770	elix-loop-helix (bHLH) DNA-binding superfamily	1.371387922	0.004651
At5g49730	ferric reduction oxidase 6	1.373942163	0.005527
At3g15850	NA	1.376771204	0.000588
At2g14520	containing protein with a domain of unknown fur	1.383143077	0.007176
At2g05632		1.390381581	0.009324
At1g66100	Plant thionin	1.394001362	0.022175
At2g16990	Major facilitator superfamily protein	1.394740268	0.005911
At1g20190	expansin 11	1.394879062	0.00034
At5g58260	ting on NADH or NADPH, quinone or similar co	1.399315253	0.003494
At4g33666		1.418701191	0.029937
At1g67865		1.435558419	0.035322
At5g63160	BTB and TAZ domain protein 1	1.436135372	0.016648
At3g44020	thylakoid lumenal P17.1 protein	1.436947358	0.026407
At4g35720	rabidopsis protein of unknown function (DUF24	1.444396994	0.049541
At2g43550	NA	1.455957007	0.001245
At4g09350	Chaperone DnaJ-domain superfamily protein	1.456789669	0.025332
At2g33750	NA	1.461296823	0.04476
At3g04140	NA	1.461549279	0.017812
At5g43750	NAD(P)H dehydrogenase 18	1.469735679	0.035508
At5g63300	Ribosomal protein S21 family protein	1.477452346	0.033727
At1g61450		1.480588786	0.018055
At5g44005		1.481248564	0.021299
At4g20140	eucine-rich repeat transmembrane protein kinas	1.483528279	0.001536
At1g52510	alpha/beta-Hydrolases superfamily protein	1.489549151	0.008851
At2g38995	NA	1.493144481	0.020247
At1g50590	RmlC-like cupins superfamily protein	1.496248689	0.040351
At1g64380	Integrase-type DNA-binding superfamily proteir	1.504142705	0.020794
At5g26200	Mitochondrial substrate carrier family protein	1.506361376	0.044999
At3g19700	NA	1.516287993	0.019837
At4g37925	)H-M of NAD(P)H:plastoquinone dehydrogenas	1.52077621	0.001365
At5g21430	Chaperone DnaJ-domain superfamily protein	1.52764194	5.80E-05
At1g70430	Protein kinase superfamily protein	1.530304083	0.003876
At3g06890	NA	1.54282178	0.021173
At1g66760	MATE efflux family protein	1.54319161	0.001293
At1g70760	inorganic carbon transport protein-related	1.54754133	0.004751
At5g15970	duced protein (KIN2) / cold-responsive protein (	1.55706412	0.002602
At2g05540	Glycine-rich protein family	1.560004233	0.006945
At3g01440	NA	1.563454998	0.019631
At1g52100	Mannose-binding lectin superfamily protein	1.564801054	0.006999
At3g19850	NA	1.56534605	0.035083
At1g68540	AD(P)-binding Rossmann-fold superfamily prote	1.565537113	0.002518
At2g43535	NA	1.572153054	0.00213

At3g52840	beta-galactosidase 2	1.584947085	0.02637
At4g12980	Auxin-responsive family protein	1.594313315	2.43E-05
At3g48420	dehalogenase-like hydrolase (HAD) superfami	1.596058258	0.008106
At2g25680	molybdate transporter 1	1.598486916	0.036927
At2g43530	NA	1.59924618	0.001523
At1g14150	PsbQ-like 2	1.606252789	0.000154
At5g16410	HXXXD-type acyl-transferase family protein	1.617041082	0.020796
At1g49500		1.649741931	0.026331
At2g23000	serine carboxypeptidase-like 10	1.651692319	0.031613
At1g78750	F-box/RNI-like superfamily protein	1.652458053	0.036949
At1g61740	Sulfite exporter TauE/SafE family protein	1.659013572	0.011084
At2g27402		1.66643799	0.023397
At4g34900	xanthine dehydrogenase 2	1.671147475	0.017108
At5g45680	FK506-binding protein 13	1.671894408	0.001424
At1g50290		1.672709284	0.044379
At3g05640	NA	1.681519687	0.001144
At1g64110	ning nucleoside triphosphate hydrolases super	1.687877926	0.003035
At3g62070		1.698831539	0.014581
At2g47780	NA	1.706451172	0.043131
At1g68238		1.708828572	0.030904
At3g21670	NA	1.708897325	0.004274
At3g22750	NA	1.718682783	0.006054
At5g48090	EDM2-like protein1	1.727056629	0.04753
At1g18265	Protein of unknown function, DUF593	1.732163383	0.002749
At1g48260	CBL-interacting protein kinase 17	1.732762761	0.018145
At2g38480	NA	1.73522831	0.008627
At4g30180	cific DNA binding transcription factors;transcrip	1.746109037	0.019637
At1g13609	Defensin-like (DEFL) family protein	1.758711631	0.013559
At1g19150	photosystem I light harvesting complex gene 6	1.767947804	0.000365
At5g02940	Protein of unknown function (DUF1012)	1.769334025	0.020929
At4g12970	stomagen	1.781872498	0.007176
At4g23870		1.78857421	0.001258
At2g32640	NA	1.804291674	0.027428
At1g53520	Chalcone-flavanone isomerase family protein	1.812767948	0.008452
At2g29290	AD(P)-binding Rossmann-fold superfamily prote	1.815699516	5.01E-06
At2g22200	Integrase-type DNA-binding superfamily protein	1.821503668	0.029836
At3g62950	Thioredoxin superfamily protein	1.823369982	0.04476
At4g25780	proteins, Antigen 5, and Pathogenesis-related	1.826900621	0.001633
At3g24420	NA	1.831386175	0.000602
At1g34580	Major facilitator superfamily protein	1.839354906	0.016044
At2g03710	egion and MADS-box transcription factor family	1.850506683	0.031361
At1g33055		1.851708729	0.021941
At1g54040	epithiospecifier protein	1.854459191	0.001033
At5g66052		1.864167075	0.002611
At3g16690	NA	1.865290731	0.000198

At1g19940	glycosyl hydrolase 9B5	1.865750708	0.020949
At2g29300	\D(P)-binding Rossmann-fold superfamily prote	1.88354934	0.000633
At1g55330	arabinogalactan protein 21	1.897330657	0.020159
At4g13575		1.900398343	0.016385
At4g12830	alpha/beta-Hydrolases superfamily protein	1.916614118	0.006917
At4g28140	ntegrase-type DNA-binding superfamily protein	1.928308223	0.016612
At3g21870	NA	1.930077407	0.007941
At2g40435	NA	1.93132844	0.024046
At1g62510	r/lipid-transfer protein/seed storage 2S albumin	1.941920451	0.029918
At1g07430	highly ABA-induced PP2C gene 2	1.957416287	0.015991
At5g46350	WRKY DNA-binding protein 8	1.964977725	0.039567
At1g69523	nethionine-dependent methyltransferases supe	1.968795969	0.004535
At4g02850	henazine biosynthesis PhzC/PhzF family protei	1.970611759	0.01388
At5g10930	CBL-interacting protein kinase 5	1.971537064	0.016385
At1g47578	Biotin/lipoate A/B protein ligase family	1.977208042	0.027768
At2g35345	NA	1.978688053	0.026853
At1g52400	beta glucosidase 18	2.021795886	0.009777
At3g45430	ıcanavalin A-like lectin protein kinase family prc	2.035539797	0.007043
At5g01015		2.038555759	0.02965
At4g10910		2.044680475	3.22E-05
At5g15240	nsmembrane amino acid transporter family pro	2.046888749	0.017864
At1g19050	response regulator 7	2.051764051	0.016416
At4g33280	AP2/B3-like transcriptional factor family protein	2.065719192	0.022732
At5g44050	MATE efflux family protein	2.099372692	0.011533
At1g61795	PAK-box/P21-Rho-binding family protein	2.100391194	0.011229
At5g24420	6-phosphogluconolactonase 5	2.112195109	0.007176
At5g58310	methyl esterase 18	2.117917982	0.000641
At1g18990	Protein of unknown function, DUF593	2.136502546	0.019309
At5g10890	myosin heavy chain-related	2.141324475	0.011342
At3g15720	NA	2.162438431	0.002194
At2g39800	NA	2.179622374	0.002804
At4g06536	ːYanodine receptor (SPRY) domain-containing	2.180831107	0.004466
At3g14440	NA	2.186106435	0.012039
At1g50732		2.190286426	0.008332
At2g28500	LOB domain-containing protein 11	2.207691315	0.006692
At4g11211		2.215403873	0.033284
At5g37300	O-acyltransferase (WSD1-like) family protein	2.237275176	0.04648
At4g23290	:eine-rich RLK (RECEPTOR-like protein kinase	2.238602578	0.003427
At1g45616	receptor like protein 6	2.240863977	0.043367
At5g43840	heat shock transcription factor A6A	2.333721875	0.001385
At1g22380	UDP-glucosyl transferase 85A3	2.334521558	8.57E-06
At3g58070	C2H2 and C2HC zinc fingers superfamily protei	2.344450929	0.003573
At4g15690	Thioredoxin superfamily protein	2.360560799	0.048765
At5g14110	Protein of unknown function (DUF 3339)	2.363857028	0.036555
At3g59250	F-box/RNI-like superfamily protein	2.380119017	0.000487

At2g22980	serine carboxypeptidase-like 13	2.395392365	0.000422
At3g13404	NA	2.419440277	0.024807
At5g04370	nethionine-dependent methyltransferases supe	2.450900776	0.005527
At1g47510	inositol polyphosphate 5-phosphatase 11	2.490957929	0.021666
At5g50360		2.519182612	0.0087
At5g65800	ACC synthase 5	2.540174845	0.046133
At4g12320	:hrome P450, family 706, subfamily A, polypept	2.545801738	6.33E-07
At3g45940	Glycosyl hydrolases family 31 protein	2.553279679	0.039006
At1g01250	Integrase-type DNA-binding superfamily proteir	2.573485375	0.037855
At5g24105	arabinogalactan protein 41	2.669483079	0.001122
Atcg00120	ATP synthase subunit alpha	2.698971812	0.025722
At5g62920	response regulator 6	2.705518672	0.00533
At5g24155	\D/NAD(P)-binding oxidoreductase family prote	2.716451735	0.011746
At3g14630	NA	2.731487321	0.003199
At2g07724		2.812399338	0.04753
At5g43290	WRKY DNA-binding protein 49	2.853687474	0.043927
At1g70640	ıpeptide/Phox/Bem1p (PB1) domain-containing	2.866398089	0.015255
At4g33467		2.904466348	0.000257
Atmg00410	ATPase subunit 6-1	2.931436549	0.033284
At1g52040	myrosinase-binding protein 1	3.025012927	1.83E-05
At2g07727	ytochrome, transmembrane;Cytochrome b/b6,	3.0295043	0.038653
Atcg00680	photosystem II reaction center protein B	3.062693449	0.026425
Atcg00140	ATP synthase subunit C family protein	3.082454781	0.028281
Atcg01060	uster binding;electron carriers;4 iron, 4 sulfur cl	3.136086002	0.019426
Atmg00220	apocytochrome b	3.195611848	0.041961
Atcg01050	ιDH-Ubiquinone/plastoquinone (complex I) prot	3.206330687	0.019826
Atcg01100	NADH dehydrogenase family protein	3.231715162	0.014974
At3g28220	NA	3.242994819	1.80E-05
At5g61350	Protein kinase superfamily protein	3.247865532	0.003302
Atcg00380	chloroplast ribosomal protein S4	3.248872289	0.032231
Atcg00530	CemA-like proton extrusion protein-related	3.285045933	0.028965
Atcg00130	ase, F0 complex, subunit B/B', bacterial/chloroן	3.297487551	0.025036
Atcg00710	photosystem II reaction center protein H	3.298522161	0.020794
Atcg00020	photosystem II reaction center protein A	3.300931877	0.04476
Atcg00490	ribulose-bisphosphate carboxylases	3.305778538	0.030344
At2g41240	NA	3.333112478	0.003985
Atcg00150	ATPase, F0 complex, subunit A protein	3.356126306	0.034033
Atmg01360	cytochrome oxidase	3.356814648	0.019443
Atcg00720	photosynthetic electron transfer B	3.360005245	0.010183
Atcg00540	photosynthetic electron transfer A	3.387164617	0.021666
Atmg00280	bisphosphate carboxylase large chain, catalyti	3.414324834	0.009937
Atmg00650	NADH dehydrogenase subunit 4L	3.435998845	0.043015
Atcg01090	NADPH dehydrogenases	3.46028505	0.010313
Atcg00520	unfolded protein binding	3.469789758	0.015407
At3g59710	\D(P)-binding Rossmann-fold superfamily prote	3.472471343	7.30E-06

Atcg00040	maturase K	3.480407621	0.020247
Atmg00400		3.484596371	0.033405
Atcg01080	·l:ubiquinone/plastoquinone oxidoreductase, ch	3.522199619	0.024317
Atcg00440	›iquinone/plastoquinone oxidoreductase, chain	3.591849586	0.039129
Atcg00340	Photosystem I, PsaA/PsaB protein	3.623360953	0.022221
Atcg00430	photosystem II reaction center protein G	3.69514678	0.020159
Atcg00350	Photosystem I, PsaA/PsaB protein	3.70285985	0.016653
Atcg00420	NADH dehydrogenase subunit J	3.703225543	0.023078
At4g35690	·abidopsis protein of unknown function (DUF24	3.709229063	0.027265
Atcg00330	chloroplast ribosomal protein S14	3.74127123	0.026853
Atcg00510	photsystem I subunit I	3.813310661	0.031352
Atcg00690	photosystem II reaction center protein T	3.819121538	0.01616
Atcg01010	Jbiquinone oxidoreductase (complex I), chain 5	3.835252614	0.021166
Atmg00640	nsporting ATP synthases, rotational mechanism	3.885848711	0.024317
Atcg00280	photosystem II reaction center protein C	3.925003725	0.018492
At3g61920		3.957336082	2.26E-05
Atcg00270	photosystem II reaction center protein D	4.022564145	0.012911
Atcg00300	YCF9	4.027767023	0.01334
Atcg00730	photosynthetic electron transfer D	4.029539449	0.001834
Atcg00630	PSAJ	4.030570437	0.035193
At4g19690	iron-regulated transporter 1	4.159118098	0.016556
Atcg00080	photosystem II reaction center protein I	4.2221003	0.034273
At4g25480	dehydration response element B1A	4.299728634	0.000884
At5g03210		4.462116854	0.002254
At1g60190	ARM repeat superfamily protein	4.639791185	0.000522
Atmg00270	NADH dehydrogenase 6	4.796173305	0.028231
At3g58060	Cation efflux family protein	5.200631993	0.035799
Genes that exhibit in roots significantly upregulated transcript levels after 3 uM concentration of DPMP (DPMP-roots_up)			
At2g30670	NA	-5.307519161	8.09E-19
At2g30660	NA	-4.715353966	1.19E-17
At1g31580	ECS1	-4.608350295	1.16E-07
At5g26690	/y metal transport/detoxification superfamily prc	-4.51582311	9.99E-08
At2g25510		-4.373286669	3.22E-08
At3g60420	Phosphoglycerate mutase family protein	-4.228685958	3.73E-06
At1g21250	cell wall-associated kinase	-4.203494948	2.36E-06
At2g26400	acireductone dioxygenase 3	-4.11959452	3.48E-19
At5g01900	WRKY DNA-binding protein 62	-3.608761908	1.52E-07
At3g12230	NA	-3.602222823	2.73E-20
At3g60470	Plant protein of unknown function (DUF247)	-3.541103251	5.16E-09
At5g52720	Copper transport protein family	-3.500866608	1.55E-07
At5g41290	.eceptor-like protein kinase-related family protei	-3.348873685	2.46E-09
At5g12020	17.6 kDa class II heat shock protein	-3.304310483	6.54E-07
At3g22231	NA	-3.291769591	1.15E-05
At2g27535	ribosomal protein L10A family protein	-3.262340219	2.10E-06

At2g23170	Auxin-responsive GH3 family protein	-3.227399033	9.01E-07
At3g50170	Plant protein of unknown function (DUF247)	-3.223093694	0.00022
At3g47480	Calcium-binding EF-hand family protein	-3.218580155	1.67E-06
At3g28510	NA	-3.140035974	1.70E-14
At1g15610		-3.13369417	2.24E-11
At2g04050	MATE efflux family protein	-3.127094314	0.000379
At2g41090	NA	-2.831930254	3.63E-05
At4g16240		-2.759336519	1.84E-05
At1g02450	NIM1-interacting 1	-2.744201581	1.44E-06
At1g52120	Mannose-binding lectin superfamily protein	-2.712678161	1.52E-07
At5g41280	eceptor-like protein kinase-related family protei	-2.677035457	2.83E-07
At2g04040	MATE efflux family protein	-2.607662329	0.000185
At5g42530		-2.606893303	0.013058
At4g10860		-2.585028271	6.01E-08
At2g14560	Protein of unknown function (DUF567)	-2.500948387	1.24E-05
At1g12805	nucleotide binding	-2.495269862	0.00472
At4g04710	calcium-dependent protein kinase 22	-2.435956978	5.17E-07
At3g01600	NA	-2.395648965	0.001427
At5g22555		-2.389205465	2.67E-06
At5g12030	heat shock protein 17.6A	-2.388067812	6.79E-06
At5g20240	egion and MADS-box transcription factor family	-2.383809355	0.003624
At2g36800	NA	-2.383255792	0.000409
At2g02580	chrome P450, family 71, subfamily B, polypepti	-2.381601359	9.55E-05
At3g46190	TRAF-like family protein	-2.370458211	2.65E-07
At5g22570	WRKY DNA-binding protein 38	-2.363109679	1.78E-07
At1g73805	Calmodulin binding protein-like	-2.350334626	0.000166
At3g21720	NA	-2.325626722	0.000621
At1g52130	Mannose-binding lectin superfamily protein	-2.322210385	1.17E-10
At3g26170	NA	-2.299946324	0.004022
At3g57260	beta-1,3-glucanase 2	-2.297081319	0.039178
At1g52700	alpha/beta-Hydrolases superfamily protein	-2.273465341	2.60E-05
At2g13810	AGD2-like defense response protein 1	-2.184903415	0.001202
At2g41730	NA	-2.07960242	0.003748
At3g11402	NA	-2.074565002	0.001563
At5g24860	flowering promoting factor 1	-2.071498636	0.012628
At5g24640		-2.045571268	0.012395
At2g45760	NA	-2.007026261	0.000207
At4g35180	LYS/HIS transporter 7	-2.000266496	0.006871
At1g15620		-1.986882123	0.014854
At3g50200	Plant protein of unknown function (DUF247)	-1.980910863	0.001179
At5g64810	WRKY DNA-binding protein 51	-1.973086253	4.28E-08
At2g18190	ning nucleoside triphosphate hydrolases super	-1.963838114	0.012983
At4g09780	TRAF-like family protein	-1.963585583	0.000975
At5g09720	Magnesium transporter CorA-like family protein	-1.960151824	1.84E-05
At4g25200	ochondrion-localized small heat shock protein 2	-1.957345647	0.003003

At2g40750	NA	-1.942900563	2.17E-06
At4g15350	:hrome P450, family 705, subfamily A, polypept	-1.920608666	0.001077
At2g05380	glycine-rich protein 3 short isoform	-1.92048343	2.60E-05
At5g40010	AAA-ATPase 1	-1.916424588	1.89E-05
At1g72930	toll/interleukin-1 receptor-like	-1.916235085	0.000854
At5g56080	nicotianamine synthase 2	-1.911736258	0.000129
At1g07900	LOB domain-containing protein 1	-1.903543715	0.004132
At4g15370	baruol synthase 1	-1.894712531	0.006244
At2g26150	heat shock transcription factor A2	-1.880586565	0.005082
At1g49570	Peroxidase superfamily protein	-1.876406588	0.012628
At1g51460	ABC-2 type transporter family protein	-1.860616041	0.007272
At3g20470	NA	-1.850194182	0.000151
At1g07180	alternative NAD(P)H dehydrogenase 1	-1.813813481	0.041258
At2g47520	NA	-1.806860989	0.004884
At5g49850	Mannose-binding lectin superfamily protein	-1.804778201	0.018046
At1g71140	MATE efflux family protein	-1.780963802	1.52E-06
At1g53540	HSP20-like chaperones superfamily protein	-1.772555754	0.017739
At3g56400	WRKY DNA-binding protein 70	-1.750429176	0.000619
At4g33560	Wound-responsive family protein	-1.745276054	0.01384
At5g11920	6-&1-fructan exohydrolase	-1.735232176	1.23E-05
At5g48175		-1.733231728	0.007352
At3g27070	NA	-1.726852567	0.002172
At1g28480	Thioredoxin superfamily protein	-1.726852173	0.002187
At1g29020	Calcium-binding EF-hand family protein	-1.714503393	1.31E-05
At1g66600	ABA overly sensitive mutant 3	-1.711155445	0.007064
At5g39100	germin-like protein 6	-1.710344613	0.004822
At1g07400	HSP20-like chaperones superfamily protein	-1.701484544	0.004041
At2g16630	ollen Ole e 1 allergen and extensin family prote	-1.698589939	1.81E-05
At3g11180	NA	-1.697027412	0.003784
At5g09710	Magnesium transporter CorA-like family protein	-1.67658481	0.01384
At1g10340	Ankyrin repeat family protein	-1.674758036	0.002258
At5g45490	ning nucleoside triphosphate hydrolases super	-1.674411116	4.96E-05
At4g39330	cinnamyl alcohol dehydrogenase 9	-1.67010061	1.15E-05
At5g45670	)SL-like Lipase/Acylhydrolase superfamily prot	-1.665989025	1.64E-05
At2g02010	glutamate decarboxylase 4	-1.66576646	0.026224
At5g25610	BURP domain-containing protein	-1.663990586	0.000127
At4g37580	-CoA N-acyltransferases (NAT) superfamily prc	-1.660916327	0.002935
At1g69930	glutathione S-transferase TAU 11	-1.65682168	0.043092
At1g10400	UDP-Glycosyltransferase superfamily protein	-1.652698444	0.012152
At4g04700	calcium-dependent protein kinase 27	-1.6474705	1.48E-05
At3g61190	BON association protein 1	-1.64147888	0.001076
At5g11930	Thioredoxin superfamily protein	-1.634486	0.032434
At2g32690	NA	-1.614514215	0.004442
At1g15630		-1.614441195	0.000679
At4g01380	plastocyanin-like domain-containing protein	-1.609610586	0.020025

At4g37370	chrome P450, family 81, subfamily D, polypepti	-1.603695484	0.028576
At5g47220	ethylene responsive element binding factor 2	-1.600336638	0.028586
At3g15650	NA	-1.598367905	0.002437
At5g05900	UDP-Glycosyltransferase superfamily protein	-1.598223291	0.002779
At5g20230	blue-copper-binding protein	-1.582676205	0.033807
At4g29020	glycine-rich protein	-1.578836661	3.02E-05
At4g30140	)SL-like Lipase/Acylhydrolase superfamily prot	-1.566952322	1.64E-05
At5g13210	haracterised conserved protein UCP015417, v	-1.563718368	0.016911
At4g28170		-1.533202193	0.028389
At3g25882	NA	-1.532588094	0.000337
At5g66780		-1.530537655	0.01552
At1g67150	Plant protein of unknown function (DUF247)	-1.529924983	0.045638
At3g50480	homolog of RPW8 4	-1.517938119	0.040006
At3g11390	NA	-1.501115357	0.023384
At2g20800	NAD(P)H dehydrogenase B4	-1.500977277	0.015177
At5g59320	lipid transfer protein 3	-1.493596801	0.000507
At4g21620	glycine-rich protein	-1.486888257	0.000312
At3g12220	NA	-1.486617867	0.012332
At4g09770	TRAF-like family protein	-1.480675121	0.001738
At1g56430	nicotianamine synthase 4	-1.477789181	0.009242
At2g18193	ning nucleoside triphosphate hydrolases super	-1.477399853	0.013879
At1g05680	Uridine diphosphate glycosyltransferase 74E2	-1.473374891	0.002325
At3g53160	UDP-glucosyl transferase 73C7	-1.469237168	0.015772
At1g60095	Mannose-binding lectin superfamily protein	-1.465249384	0.00016
At4g12490	r/lipid-transfer protein/seed storage 2S albumin	-1.452392497	0.031734
At2g15090	3-ketoacyl-CoA synthase 8	-1.451967573	0.013523
At5g45470	Protein of unknown function (DUF594)	-1.450083969	0.001273
At4g19980		-1.448383055	0.020219
At2g45360	NA	-1.447987753	0.039178
At5g17760	ning nucleoside triphosphate hydrolases super	-1.447060879	0.000535
At5g22800	Alanyl-tRNA synthetase, class IIc	-1.444155498	0.001005
At5g14760	L-aspartate oxidase	-1.438973932	0.000459
At1g54050	HSP20-like chaperones superfamily protein	-1.43106569	0.039388
At1g19960		-1.418608872	0.000229
At1g02230	NAC domain containing protein 4	-1.417360103	0.001102
At4g25850	SBP(oxysterol binding protein)-related protein 4	-1.41108782	0.007711
At5g39090	HXXXD-type acyl-transferase family protein	-1.396701653	0.014254
At1g59860	HSP20-like chaperones superfamily protein	-1.396491775	0.036364
At2g27550	centroradialis	-1.396223647	0.001096
At2g02250	phloem protein 2-B2	-1.374973706	0.014158
At1g11600	chrome P450, family 77, subfamily B, polypepti	-1.373601152	0.021911
At3g13090	NA	-1.372218979	0.004858
At5g64060	NAC domain containing protein 103	-1.37204656	0.034419
At4g23260	teine-rich RLK (RECEPTOR-like protein kinase	-1.368710073	0.009507
At3g19200	NA	-1.359103354	0.009358

At1g63750	ease resistance protein (TIR-NBS-LRR class) fa	-1.351059841	0.004455
At5g25450	Cytochrome bd ubiquinol oxidase, 14kDa subun	-1.333515879	0.006387
At5g22300	nitrilase 4	-1.332246264	0.020038
At4g13890	osphate (PLP)-dependent transferases superfa	-1.327377485	0.005913
At1g71870	MATE efflux family protein	-1.324601937	0.045638
At1g15640		-1.323483673	0.004736
At2g30750	NA	-1.311292419	0.008711
At5g45110	NPR1-like protein 3	-1.300009739	0.000621
At5g52640	heat shock protein 90.1	-1.291960349	0.048243
At3g19990	NA	-1.28933614	0.006849
At3g23450	NA	-1.286589628	0.03808
At3g08770	NA	-1.284407816	0.006243
At5g13400	Major facilitator superfamily protein	-1.281622096	0.03514
At1g70810	-dependent lipid-binding (CaLB domain) family	-1.279737765	0.000829
At4g04570	teine-rich RLK (RECEPTOR-like protein kinase	-1.274890612	0.039146
At2g05520	glycine-rich protein 3	-1.270480071	0.001674
At5g45070	phloem protein 2-A8	-1.264288562	0.002252
At3g60140	Glycosyl hydrolase superfamily protein	-1.262552593	0.002345
At3g06020	NA	-1.260999683	0.014942
At4g10500	e (2OG) and Fe(II)-dependent oxygenase super	-1.251423338	0.002302
At3g25620	NA	-1.239694078	0.022264
At5g39160	RmlC-like cupins superfamily protein	-1.232382452	0.015254
At5g12890	UDP-Glycosyltransferase superfamily protein	-1.232202956	0.031785
At5g03545		-1.218735029	0.0029
At5g16980	Zinc-binding dehydrogenase family protein	-1.199088425	0.043381
At2g38530	NA	-1.180697873	0.011695
At5g39130	RmlC-like cupins superfamily protein	-1.175350443	0.031093
At2g26820	phloem protein 2-A3	-1.173866017	0.035878
At2g42840	NA	-1.165960457	0.005945
At5g16970	alkenal reductase	-1.155772831	0.004575
At3g47050	Glycosyl hydrolase family protein	-1.136271227	0.020465
At5g38900	Thioredoxin superfamily protein	-1.133180576	0.008637
At2g27660	Cysteine/Histidine-rich C1 domain family protei	-1.121113158	0.011034
At1g60750	AD(P)-linked oxidoreductase superfamily prote	-1.117573251	0.038465
At1g68530	3-ketoacyl-CoA synthase 6	-1.112137021	0.005148
At4g14390	Ankyrin repeat family protein	-1.107794674	0.015422
At2g43820	NA	-1.09926709	0.015177
At3g48180		-1.09805248	0.039178
At1g26770	expansin A10	-1.094876075	0.036364
At2g37770	NA	-1.08018661	0.018993
At2g39370	NA	-1.078652459	0.034799
At1g09310	Protein of unknown function, DUF538	-1.072408117	0.021901
At2g43570	NA	-1.067432402	0.038205
At4g08780	Peroxidase superfamily protein	-1.061649878	0.020376
At2g16060	hemoglobin 1	-1.058169593	0.048243

At2g41480	NA	-1.056211575	0.045638
At5g13370	Auxin-responsive GH3 family protein	-1.054194611	0.033191
At3g22235	NA	-1.043433373	0.018674
At5g38340	ease resistance protein (TIR-NBS-LRR class) fa	-1.03311685	0.040372
At2g15490	UDP-glycosyltransferase 73B4	-1.030383012	0.047185
At5g39050	HXXXD-type acyl-transferase family protein	-1.015358571	0.018091
At1g68880	basic leucine-zipper 8	-1.011220761	0.021162
At5g09290	Inositol monophosphatase family protein	-1.003158481	0.02322
At3g15300	NA	-0.978361024	0.037748
At4g08555		-0.972572071	0.040372
At1g78340	glutathione S-transferase TAU 22	-0.97042721	0.044426
Genes that exhibit in roots significantly downregulated transcript levels after 3 uM concentration of DPMP (DPMP-roots_down)			
At3g44550	fatty acid reductase 5	0.973666424	0.031922
At1g22550	Major facilitator superfamily protein	0.975976263	0.019373
At3g55720	Protein of unknown function (DUF620)	0.978446961	0.032508
At1g78260	NA-binding (RRM/RBD/RNP motifs) family prote	0.978897893	0.027806
At5g57625	proteins, Antigen 5, and Pathogenesis-related	0.982340253	0.028344
At3g60330	H(+)-ATPase 7	0.985526399	0.027993
At1g05810	RAB GTPase homolog A5E	0.989078157	0.042549
At5g42590	chrome P450, family 71, subfamily A, polypeptic	0.991212601	0.029904
At4g39950	chrome P450, family 79, subfamily B, polypepti	0.992161318	0.047703
At4g00230	xylem serine peptidase 1	1.006471333	0.017096
At2g34490	NA	1.010273401	0.017739
At4g26320	arabinogalactan protein 13	1.010388765	0.027085
At5g63590	flavonol synthase 3	1.012775793	0.036394
At1g19230	Riboflavin synthase-like superfamily protein	1.014728244	0.012613
At5g23210	serine carboxypeptidase-like 34	1.0155876	0.017841
At5g58010	LJRHL1-like 3	1.020499551	0.016042
At5g06930		1.024862268	0.038465
At1g52050	Mannose-binding lectin superfamily protein	1.025116472	0.032434
At5g53250	arabinogalactan protein 22	1.03539442	0.020025
At4g26140	beta-galactosidase 12	1.036024644	0.019214
At2g37980	NA	1.040258069	0.034419
At3g46700	UDP-Glycosyltransferase superfamily protein	1.043583262	0.013247
At5g09520	hydroxyproline-rich glycoprotein family protein	1.044831493	0.045638
At3g26300	NA	1.044877541	0.045638
At5g04970	invertase/pectin methylesterase inhibitor super	1.04605787	0.017739
At5g17820	Peroxidase superfamily protein	1.046197765	0.042505
At4g02270	root hair specific 13	1.052614647	0.007499
At1g14240	DA1/CD39 nucleoside phosphatase family prote	1.053607803	0.039178
At1g17190	glutathione S-transferase tau 26	1.053693927	0.011661
At3g09925	NA	1.056012946	0.011538
At1g48090	calcium-dependent lipid-binding family protein	1.056132473	0.015248

At4g10770	oligopeptide transporter 7	1.056934873	0.014158
At2g37440	NA	1.057356879	0.013561
At3g26330	NA	1.059134817	0.011661
At4g19230	:hrome P450, family 707, subfamily A, polypept	1.059738743	0.015527
At2g38760	NA	1.066333988	0.007913
At1g08500	early nodulin-like protein 18	1.066714188	0.013885
At1g73340	Cytochrome P450 superfamily protein	1.072142215	0.044642
At5g04960	invertase/pectin methylesterase inhibitor super	1.072162597	0.009209
At1g64590	AD(P)-binding Rossmann-fold superfamily prote	1.073028629	0.040006
At5g01050	Laccase/Diphenol oxidase family protein	1.078816763	0.045638
At1g53830	pectin methylesterase 2	1.079215474	0.009283
At1g28100		1.080511405	0.032434
At4g24580	GTPase activation protein (RhoGAP) with PH do	1.081644244	0.010065
At5g51890	Peroxidase superfamily protein	1.082893346	0.011021
At1g04700	PB1 domain-containing protein tyrosine kinase	1.084971664	0.028563
At5g60890	myb domain protein 34	1.085419036	0.044953
At1g55240	Family of unknown function (DUF716)	1.087577076	0.019314
At5g22410	root hair specific 18	1.090283545	0.007502
At3g62680	proline-rich protein 3	1.092112009	0.012628
At1g52750	alpha/beta-Hydrolases superfamily protein	1.095352645	0.011562
At4g30320	proteins, Antigen 5, and Pathogenesis-related	1.097313683	0.040372
At3g48520	chrome P450, family 94, subfamily B, polypepti	1.098172302	0.04218
At1g69870	nitrate transporter 1.7	1.114764891	0.008582
At5g17700	MATE efflux family protein	1.11665419	0.033524
At5g44530	Subtilase family protein	1.1191834	0.025219
At5g49900	Beta-glucosidase, GBA2 type family protein	1.121267814	0.008739
At5g61650	CYCLIN P4;2	1.124889476	0.037404
At4g00460	RHO guanyl-nucleotide exchange factor 3	1.127812337	0.027085
At2g17500	Auxin efflux carrier family protein	1.139622396	0.048207
At5g56540	arabinogalactan protein 14	1.148834128	0.011562
At5g47950	HXXXD-type acyl-transferase family protein	1.149530728	0.004455
At2g38080	NA	1.150905628	0.004736
At3g62390	TRICHOME BIREFRINGENCE-LIKE 6	1.15137969	0.022929
At2g38750	NA	1.151542237	0.009435
At2g22125	binding	1.156025602	0.020465
At4g11310	Papain family cysteine protease	1.157721761	0.044638
At2g43535	NA	1.158873131	0.031922
At3g48350	Cysteine proteinases superfamily protein	1.161416296	0.026921
At4g11190	esistance-responsive (dirigent-like protein) fam	1.161853457	0.004884
At4g23680	cyclase/dehydrase and lipid transport superfar	1.162071576	0.007799
At4g15230	pleiotropic drug resistance 2	1.163917782	0.004858
At3g47950	H(+)-ATPase 4	1.164278642	0.007442
At3g14850	NA	1.166355602	0.040372
At1g70460	root hair specific 10	1.169043897	0.017162
At5g61350	Protein kinase superfamily protein	1.177676222	0.011348

At2g27920	serine carboxypeptidase-like 51	1.178224692	0.017999
At5g61340		1.178748794	0.032219
At3g18773	NA	1.183468153	0.044307
At1g76020	Thioredoxin superfamily protein	1.183769929	0.027424
At5g11110	sucrose phosphate synthase 2F	1.18378763	0.009505
At1g16440	root hair specific 3	1.191655822	0.040398
At1g18140	laccase 1	1.19243067	0.003282
At5g15600	SPIRAL1-like4	1.195721336	0.025686
At1g35330	RING/U-box superfamily protein	1.196150291	0.021042
At5g58784	ecaprenyl pyrophosphate synthetase family prc	1.19765904	0.045858
At3g05150	NA	1.199049648	0.033468
At2g42850	NA	1.200657274	0.026896
At3g20460	NA	1.20142825	0.005082
At4g25250	ertase/pectin methylesterase inhibitor superfam	1.204996188	0.003202
At5g38970	brassinosteroid-6-oxidase 1	1.214405556	0.044953
At5g23110	nc finger, C3HC4 type (RING finger) family prot	1.215961129	0.004442
At3g18080	NA	1.217314431	0.028541
At5g06800	yb-like HTH transcriptional regulator family prot	1.217464211	0.04207
At1g72140	Major facilitator superfamily protein	1.219018836	0.002302
At3g21340	NA	1.236641666	0.03785
At1g78950	Terpenoid cyclases family protein	1.238702986	0.038158
At1g52240	RHO guanyl-nucleotide exchange factor 11	1.243529066	0.035779
At3g62020	germin-like protein 10	1.24368825	0.004536
At4g15215	pleiotropic drug resistance 13	1.243979161	0.013969
At5g36140	chrome P450, family 716, subfamily A, polypept	1.245968661	0.01008
At5g15410	clic nucleotide-regulated ion channel family prot	1.247268765	0.001652
At1g06090	Fatty acid desaturase family protein	1.250182147	0.015173
At2g21540	SEC14-like 3	1.253390121	0.035878
At2g37700	NA	1.254964974	0.027085
At1g52190	Major facilitator superfamily protein	1.255006593	0.026574
At5g10770	Eukaryotic aspartyl protease family protein	1.261119592	0.002337
At2g22920	serine carboxypeptidase-like 12	1.26231726	0.045372
At3g59710	AD(P)-binding Rossmann-fold superfamily prote	1.263279163	0.009947
At1g77520	O-methyltransferase family protein	1.263752408	0.005844
At1g09540	myb domain protein 61	1.264021141	0.032434
At4g25220	root hair specific 15	1.264588441	0.00087
At5g37690	GNH hydrolase-type esterase superfamily prote	1.264821178	0.018247
At5g42785		1.272127934	0.027173
At4g04460	Saposin-like aspartyl protease family protein	1.272837444	0.002302
At2g22970	serine carboxypeptidase-like 11	1.274790432	0.02488
At2g32620	NA	1.276844942	0.032434
At2g18210		1.277235783	0.01008
At3g59340	Eukaryotic protein of unknown function (DUF914	1.277998769	0.028307
At1g11190	bifunctional nuclease i	1.278857417	0.003065
Atmg01360	cytochrome oxidase	1.280510791	0.042505

At3g56000	cellulose synthase like A14	1.285238722	0.007402
At1g04160	myosin XI B	1.285785233	0.002456
At3g55290	\D(P)-binding Rossmann-fold superfamily prote	1.290042484	0.017739
At5g02170	nsmembrane amino acid transporter family pro	1.295636321	0.002337
At4g33120	nethionine-dependent methyltransferases supe	1.297873908	0.000799
At4g15233	2 and Plant PDR ABC-type transporter family p	1.299902366	0.012541
At3g29260	NA	1.302681508	0.036453
At3g62740	beta glucosidase 7	1.304811299	0.026939
At5g36130	Cytochrome P450 superfamily protein	1.305615622	0.008352
At4g26690	PLC-like phosphodiesterase family protein	1.306849319	0.003419
At2g01880	purple acid phosphatase 7	1.307686252	0.001713
At5g40890	chloride channel A	1.313368084	0.00335
At3g57630	exostosin family protein	1.322135377	0.035878
At1g73280	serine carboxypeptidase-like 3	1.32332709	0.020376
At3g43930	BRCT domain-containing DNA repair protein	1.328652505	0.049183
At3g52820	purple acid phosphatase 22	1.333031027	0.020078
At1g77530	O-methyltransferase family protein	1.33386379	0.003419
At5g07080	HXXXD-type acyl-transferase family protein	1.344058569	0.008739
At5g62310	dent, cGMP-dependent and protein kinase C) k	1.350831203	0.001417
At2g01520	MLP-like protein 328	1.361009263	0.042634
At5g36180	serine carboxypeptidase-like 1	1.361309008	0.013798
At4g28940	Phosphorylase superfamily protein	1.363309872	0.000573
At4g29270	AD superfamily, subfamily IIIB acid phosphatas	1.363529067	0.019289
At1g75620	glyoxal oxidase-related protein	1.364601134	0.012653
At5g43230		1.366893322	0.0168
At5g36150	putative pentacyclic triterpene synthase 3	1.367257491	0.000619
At1g43160	related to AP2 6	1.368144856	0.032434
At5g35940	Mannose-binding lectin superfamily protein	1.371601589	0.000651
At3g47740	ABC2 homolog 2	1.376083423	0.02004
At1g27740	root hair defective 6-like 4	1.376208359	0.001005
At4g25410	elix-loop-helix (bHLH) DNA-binding superfamily	1.380548786	0.003602
At1g31710	Copper amine oxidase family protein	1.381570209	0.000203
At5g07130	laccase 13	1.382930541	0.02322
At4g19680	iron regulated transporter 2	1.389526881	0.00578
At1g06520	glycerol-3-phosphate acyltransferase 1	1.390276287	0.019584
At4g12520	r/lipid-transfer protein/seed storage 2S albumin	1.391222573	0.003748
At4g12510	r/lipid-transfer protein/seed storage 2S albumin	1.392288639	0.001031
At2g34350	NA	1.393245053	0.014098
At4g26010	Peroxidase superfamily protein	1.393983021	0.001076
At2g35990	NA	1.395018596	0.01499
At5g49360	beta-xylosidase 1	1.398662816	0.000902
At3g05155	NA	1.399900328	0.02322
At2g37130	NA	1.406411719	0.017888
At3g46170	\D(P)-binding Rossmann-fold superfamily prote	1.411405499	0.007064
At1g22330	NA-binding (RRM/RBD/RNP motifs) family prote	1.420138362	0.001223

At1g65310	xyloglucan endotransglucosylase/hydrolase 17	1.4278017	0.000434
At2g31141	NA	1.437002067	0.025933
At3g62160	HXXXD-type acyl-transferase family protein	1.437176176	0.015772
At1g73300	serine carboxypeptidase-like 2	1.440245398	0.018307
At2g01530	MLP-like protein 329	1.445218607	0.004736
At3g29430	NA	1.447450138	0.016414
At2g16970	Major facilitator superfamily protein	1.455291396	0.042563
At2g20520	FASCICLIN-like arabinogalactan 6	1.457068907	0.000193
At4g13620	Integrase-type DNA-binding superfamily protein	1.463358914	0.017739
At5g23840	2-related lipid recognition domain-containing pr	1.470963066	0.001331
At1g05660	Pectin lyase-like superfamily protein	1.472802057	0.002325
At1g14960	cyclase/dehydrase and lipid transport superfar	1.473131021	0.000409
At5g03260	laccase 11	1.473686557	0.002437
At3g16530	NA	1.477381343	0.02322
At1g15405	other RNA	1.482885492	0.002456
At1g04330		1.489021054	0.006939
At2g23410	cis-prenyltransferase	1.4925985	0.011169
At3g18200	NA	1.49283933	0.006827
Atcg00500	l-CoA carboxylase carboxyl transferase subuni	1.498806361	0.006948
At2g14095		1.500946516	0.030609
At5g10230	annexin 7	1.501431729	0.000169
At5g63450	chrome P450, family 94, subfamily B, polypepti	1.502363238	0.000105
At5g02000		1.50343884	0.019366
At1g02310	Glycosyl hydrolase superfamily protein	1.509240628	0.016199
At1g23760	BURP domain-containing protein	1.511201928	0.000574
At5g03640	Protein kinase superfamily protein	1.515213278	0.001713
At3g16690	NA	1.517702424	0.00893
At5g24105	arabinogalactan protein 41	1.523696232	0.012628
At2g14760	elix-loop-helix (bHLH) DNA-binding superfamily	1.523967629	0.019991
At1g54970	proline-rich protein 1	1.525575965	1.44E-05
At4g29180	root hair specific 16	1.527608935	4.46E-05
At1g63450	root hair specific 8	1.529910583	0.005329
At5g47980	HXXXD-type acyl-transferase family protein	1.530548716	9.00E-05
At5g60020	laccase 17	1.532193232	0.00087
At4g25160	ox domain-containing protein kinase family pro	1.532586457	0.005525
At1g10360	glutathione S-transferase TAU 18	1.533261955	0.049731
At5g21080	Uncharacterized protein	1.533464874	0.012332
At5g55050	DSL-like Lipase/Acylhydrolase superfamily prote	1.534969402	0.017477
At2g17890	calcium-dependent protein kinase 16	1.538265719	0.015177
At5g45840	Leucine-rich repeat protein kinase family protein	1.538369825	0.000831
At3g55310	AD(P)-binding Rossmann-fold superfamily prote	1.539772129	0.018993
At3g14440	NA	1.541491014	0.014158
At1g61080	Hydroxyproline-rich glycoprotein family protein	1.542152785	0.024963
At2g31310	NA	1.542285085	0.035878
At1g55290	e (2OG) and Fe(II)-dependent oxygenase super	1.546301084	0.013258

At2g33205	NA	1.546509094	0.005054
At5g24780	vegetative storage protein 1	1.548311776	0.001415
Atcg01110	NAD(P)H dehydrogenase subunit H	1.550171121	0.036364
At4g13280	terpenoid synthase 12	1.550820522	0.002258
At1g62510	r/lipid-transfer protein/seed storage 2S albumin	1.55128307	0.048698
At1g32100	pinoresinol reductase 1	1.555900062	0.00607
At2g40113	NA	1.565137287	0.032434
At5g15180	Peroxidase superfamily protein	1.567273195	0.007272
At4g37700		1.5698618	0.002332
At1g66800	AD(P)-binding Rossmann-fold superfamily prote	1.573927438	1.16E-05
Atcg00040	maturase K	1.574886992	0.013798
At2g35210	NA	1.575235504	0.009878
At2g43470	NA	1.577670255	0.045638
At5g24770	vegetative storage protein 2	1.578686961	0.001319
At1g73860	ning nucleoside triphosphate hydrolases super	1.58127404	0.004736
At2g23010	serine carboxypeptidase-like 9	1.583002899	0.021321
At1g45015	2-related lipid recognition domain-containing pr	1.583391218	0.011888
At1g60050	odulin MtN21 /EamA-like transporter family prot	1.583391249	0.002064
At1g47610	Transducin/WD40 repeat-like superfamily protei	1.58454656	0.044794
At4g13390	Proline-rich extensin-like family protein	1.587994635	1.16E-05
At3g27884	NA	1.597194349	0.005913
At1g51470	beta glucosidase 35	1.604219212	0.000693
At1g21100	O-methyltransferase family protein	1.6096869	1.78E-05
At5g38020	nethionine-dependent methyltransferases supe	1.620342671	0.000216
At5g47450	tonoplast intrinsic protein 2;3	1.622048785	0.000406
At2g35980	NA	1.629236527	0.000178
At5g42210	Major facilitator superfamily protein	1.631888674	0.009507
At3g03500	NA	1.634600951	0.0014
At1g34510	Peroxidase superfamily protein	1.638944355	1.64E-05
At4g28850	xyloglucan endotransglucosylase/hydrolase 26	1.639251495	0.002593
At2g16980	Major facilitator superfamily protein	1.6407131	0.000986
Atcg00480	ATP synthase subunit beta	1.652756955	0.044534
At2g25150	HXXXD-type acyl-transferase family protein	1.658122343	3.07E-06
At2g35750	NA	1.659644064	0.035878
At1g47600	beta glucosidase 34	1.660850726	0.000167
At5g56870	beta-galactosidase 4	1.6848394	3.57E-05
At5g65690	phosphoenolpyruvate carboxykinase 2	1.685366258	0.000113
At5g44390	FAD-binding Berberine family protein	1.694652213	0.002462
At3g47760	ABC2 homolog 4	1.696236783	0.039178
At3g45680	Major facilitator superfamily protein	1.696878235	9.30E-06
At1g22290	14-3-3 family protein	1.698813278	0.017096
At2g35890	NA	1.700468963	0.002152
At5g35380	protein with adenine nucleotide alpha hydrolas	1.702622473	0.037067
At1g30370	alpha/beta-Hydrolases superfamily protein	1.704274145	0.011145
At5g44130	SCICLIN-like arabinogalactan protein 13 precur	1.714248641	0.022222

At1g58520	lipases;hydrolases, acting on ester bonds	1.714947462	0.000416
At5g15290	Uncharacterised protein family (UPF0497)	1.725483512	0.002131
At1g25230	:urin-like metallo-phosphoesterase superfamily	1.730015021	0.000467
At1g09890	Rhamnogalacturonate lyase family protein	1.732548924	0.019314
At1g09170	hate hydrolases superfamily protein with CH (C	1.732801135	0.001166
At2g34010	NA	1.734208429	0.028586
At5g57530	xyloglucan endotransglucosylase/hydrolase 12	1.737021401	4.29E-06
At1g14120	ɘ (2OG) and Fe(II)-dependent oxygenase supe	1.737993828	1.68E-05
At1g52410	TSK-associating protein 1	1.741842441	2.17E-06
At3g22570	NA	1.744020373	0.003202
At4g18425	Protein of unknown function (DUF679)	1.746258845	0.003602
At1g14220	Ribonuclease T2 family protein	1.74732028	0.002209
At5g46900	r/lipid-transfer protein/seed storage 2S albumin	1.751075224	2.17E-06
At1g14185	-methanol-choline (GMC) oxidoreductase famil	1.755539113	0.001981
At3g49780	phytosulfokine 4 precursor	1.762939816	0.008818
At3g25830	NA	1.769113901	0.000166
At1g52790	ɘ (2OG) and Fe(II)-dependent oxygenase supe	1.771033805	0.018514
At1g05310	Pectin lyase-like superfamily protein	1.772045141	0.026939
At4g12545	r/lipid-transfer protein/seed storage 2S albumin	1.77322593	9.01E-07
At1g25240	ENTH/VHS/GAT family protein	1.778352724	0.012297
At4g36410	ubiquitin-conjugating enzyme 17	1.782679626	0.013798
At3g61890	homeobox 12	1.78340457	0.035868
At5g60660	plasma membrane intrinsic protein 2;4	1.790733176	1.66E-07
At5g61550	ox domain-containing protein kinase family pro	1.791079226	1.88E-05
At5g37990	nethionine-dependent methyltransferases supe	1.79415296	0.001005
At4g37220	Cold acclimation protein WCOR413 family	1.794574614	0.012179
At3g19320	NA	1.797133809	0.005549
At2g07671	ATP synthase subunit C family protein	1.801504515	0.022122
At5g53380	O-acyltransferase (WSD1-like) family protein	1.812589526	0.037868
At4g08290	dulin MtN21 /EamA-like transporter family prot	1.82407349	0.001994
At2g26690	Major facilitator superfamily protein	1.827247324	0.000483
At3g15356	NA	1.837617261	0.000927
At3g25820	NA	1.838155065	9.55E-05
At4g15290	Cellulose synthase family protein	1.838228326	0.001038
At4g30420	dulin MtN21 /EamA-like transporter family prot	1.839320676	0.002692
At3g10740	NA	1.847842028	0.00051
At1g54790	)SL-like Lipase/Acylhydrolase superfamily prot	1.850572085	0.010782
At2g31540	NA	1.857601698	0.025156
At4g00780	TRAF-like family protein	1.861365482	0.0029
At1g18870	isochorismate synthase 2	1.863750691	0.000112
At3g06070	NA	1.866896083	0.006083
At1g06120	Fatty acid desaturase family protein	1.869086775	0.000108
At1g50930		1.874064413	0.009472
At1g06923		1.879756119	0.003066
At5g21120	ETHYLENE-INSENSITIVE3-like 2	1.880269201	0.025653

At3g09330	NA	1.882958816	0.012653
At5g23980	ferric reduction oxidase 4	1.889920139	0.010257
At2g37450	NA	1.897120439	0.000304
At5g04120	Phosphoglycerate mutase family protein	1.941833009	1.96E-06
At2g44110	NA	1.952967045	1.25E-06
At5g53902	U3B; snoRNA	1.958066296	0.015929
At3g21180	NA	1.969565745	1.16E-06
At3g45700	Major facilitator superfamily protein	1.984538473	0.000112
At5g52300	CAP160 protein	1.989691735	0.000495
At3g49190	O-acyltransferase (WSD1-like) family protein	2.018820507	5.49E-06
At5g46890	r/lipid-transfer protein/seed storage 2S albumin	2.022600527	4.47E-07
Atmg00270	NADH dehydrogenase 6	2.05522842	0.02277
At5g04370	nethionine-dependent methyltransferases supe	2.058052888	0.000831
At2g31083	NA	2.063019108	0.001319
At2g42250	NA	2.070031825	8.06E-05
At5g36870	glucan synthase-like 9	2.07504062	0.016897
At1g15580	indole-3-acetic acid inducible 5	2.091446897	0.031093
At3g45130	lanosterol synthase 1	2.094356942	4.55E-05
At2g42900	NA	2.110033735	0.006082
At5g61290	Flavin-binding monooxygenase family protein	2.12522544	0.003033
At2g33790	NA	2.180801896	3.27E-09
At5g26250	Major facilitator superfamily protein	2.181739433	0.001417
At5g52260	myb domain protein 19	2.197102426	0.005446
At1g52800	ɘ (2OG) and Fe(II)-dependent oxygenase supeı	2.198399779	1.96E-06
At3g21670	NA	2.207188406	5.70E-07
At1g45616	receptor like protein 6	2.299493712	0.008975
At5g42580	hrome P450, family 705, subfamily A, polypepti	2.311103244	2.82E-07
At1g19900	glyoxal oxidase-related protein	2.351210793	0.000151
At5g24140	squalene monooxygenase 2	2.373724287	1.25E-12
At3g56825	U2.4; snRNA	2.384406895	0.039913
At4g39770	dehalogenase-like hydrolase (HAD) superfami	2.422318871	0.000755
At2g26695	Ran BP2/NZF zinc finger-like superfamily protei	2.430810585	0.035878
At5g65800	ACC synthase 5	2.442035885	1.24E-05
At4g12550	Auxin-Induced in Root cultures 1	2.449731238	1.38E-10
At4g20450	Leucine-rich repeat protein kinase family proteiı	2.474849083	0.001713
At2g39040	NA	2.519026153	1.38E-09
At5g42600	marneral synthase	2.565855295	1.88E-14
At2g16005	2-related lipid recognition domain-containing pr	2.650402929	3.65E-07
At3g57765	U2.3; snRNA	2.700835064	0.02258
At4g01830	P-glycoprotein 5	2.787491142	5.91E-09
At3g56705	U2.6; snRNA	2.809652781	0.016911
At2g31085	NA	2.837685454	0.000201
At5g06900	chrome P450, family 93, subfamily D, polypepti	2.884450313	2.58E-07
At5g09585	U2.5; snRNA	2.993024105	0.008222
At5g61455	U2.7; snRNA	3.106724654	0.003101

At1g52820	e (2OG) and Fe(II)-dependent oxygenase super	3.110485866	1.88E-14
At3g48740	Nodulin MtN3 family protein	3.233682417	6.01E-08
At1g15540	e (2OG) and Fe(II)-dependent oxygenase super	3.356383857	4.30E-06
At5g06905	chrome P450, family 712, subfamily A, polypept	4.449996068	4.06E-12
At3g52970	chrome P450, family 76, subfamily G, polypepti	4.61745565	1.21E-12

SUPER ACIDS: 75 genes that are commonly upregulated by DPMP and BHTC

AT5G24530
AT3G14620
AT2G25510
AT1G74440
AT1G03850
AT2G14560
AT1G21250
AT5G39020
AT2G47130
AT3G09020
AT3G56400
AT2G31880
AT3G56710
AT1G13750
AT3G50480
AT5G44820
AT5G25440
AT2G40750
AT4G38550
AT4G33050
AT3G09490
AT4G37370
AT1G66880
AT5G45380
AT5G03350
AT5G55170
AT4G08850
AT5G60950
AT3G11840
AT4G20110
AT2G26440
AT1G10340
AT5G52760
AT5G52750

AT1G02360
AT4G23220
AT3G45860
AT1G67800
AT2G15390
AT5G39670
AT3G25610
AT1G07000
AT4G01700
AT2G23680
AT2G46400
AT2G17040
AT1G35710
AT1G24140
AT3G48090
AT4G11890
AT2G19130
AT1G73805
AT2G26560
AT1G01560
AT1G18390
AT2G29120
AT5G55450
AT5G66640
AT4G23610
AT4G11000
AT5G60900
AT5G22570
AT1G35210
AT4G39830
AT1G13470
AT1G24150
AT1G34420
AT5G67340
AT2G27660
AT2G32680
AT1G01340
AT3G50140
AT3G01080
AT4G18250
AT3G50930

ɟers Strong

e Gomez2,
