# Supplementary material for: The Synthetic Elicitor DPMP (2,4-dichloro-6-{(E)-[(3-methoxyphenyl)imino]methyl}phenol) Triggers Strong Immunity in Arabidopsis thaliana and Tomato
Source: Sci Rep. 2016 Jul 14;6:29554. doi: 10.1038/srep29554 (PMC4944173; doi:10.1038/srep29554)
Supplement: Supplementary Figures [file srep29554-s2.pdf]

The Synthetic Elicitor DPMP (2,4-dichloro-6-{(E)-[(3-methoxyphenyl)imino]methyl}phenol) Triggers Strong Immunity in *Arabidopsis thaliana* and Tomato

Yasemin Bektas [1, 2, @], Melinda Rodriguez-Salus [1, 2, 3], Mercedes Schroeder [1, 2, 3], Adilene Gomez[2], Isgouhi Kaloshian [1, 4], Thomas Eulgem [1, 2, 3, *]

[1]: Center for Plant Cell Biology, Institute for Integrative Genome Biology, University of California at Riverside, CA 92521, USA

[2]: Department of Botany and Plant Sciences, University of California at Riverside, CA 92521, USA

[3]: ChemGen Intergrative Graduate Education and Research Traineeship program, program, University of California at Riverside, CA 92521, USA

[4]: Department of Nematology, University of California at Riverside, CA 92521, USA

[*]: Corresponding author: Thomas Eulgem, E-mail: thomas.eulgem@ucr.edu

[@]: Present address of Yasemin Bektas: Department of Molecular Biology and Genetics, Faculty of Science and Arts, Gaziosmanpaşa University, Tokat, Turkey

Supplementary Figures

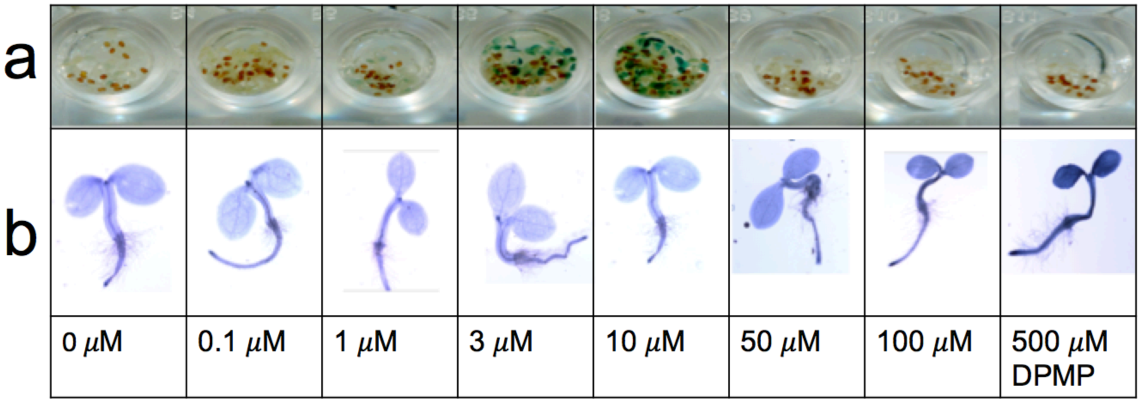

Supplementary Figure S1 online: Activity of DPMP in *CaBP22*$^{-333}$*::GUS* Arabidopsis plants.

(**a**) After 24 h incubation with the indicated DPMP concentrations, wells of "96-well plates" containing 7d-old liquid-grown *CaBP22*$^{-333}$*::GUS* seedlings were processed by X-Gluc histochemical staining. Blue/green color of cotyledons treated with 1 to 10 µM DPMP indicates induction of *GUS* gene expression. Chlorophyll was completely removed during the ethanol-destaining process after GUS staining.

(b) Trypan blue staining of *CaBP22*$^{-333}$*::GUS* seedlings incubated for 24 h in liquid medium containing DPMP at the indicated concentrations. Dark blue/black color of the cotyledons indicates cell death (toxicity). The seed coats of seedlings always stain blue/black and can been seen in some images. Extensive cell death is only detectable after treatment with 500 µM DPMP. **(A, B)** All histochemical staining analyses were performed at least three times with similar results. Shown are typical examples.

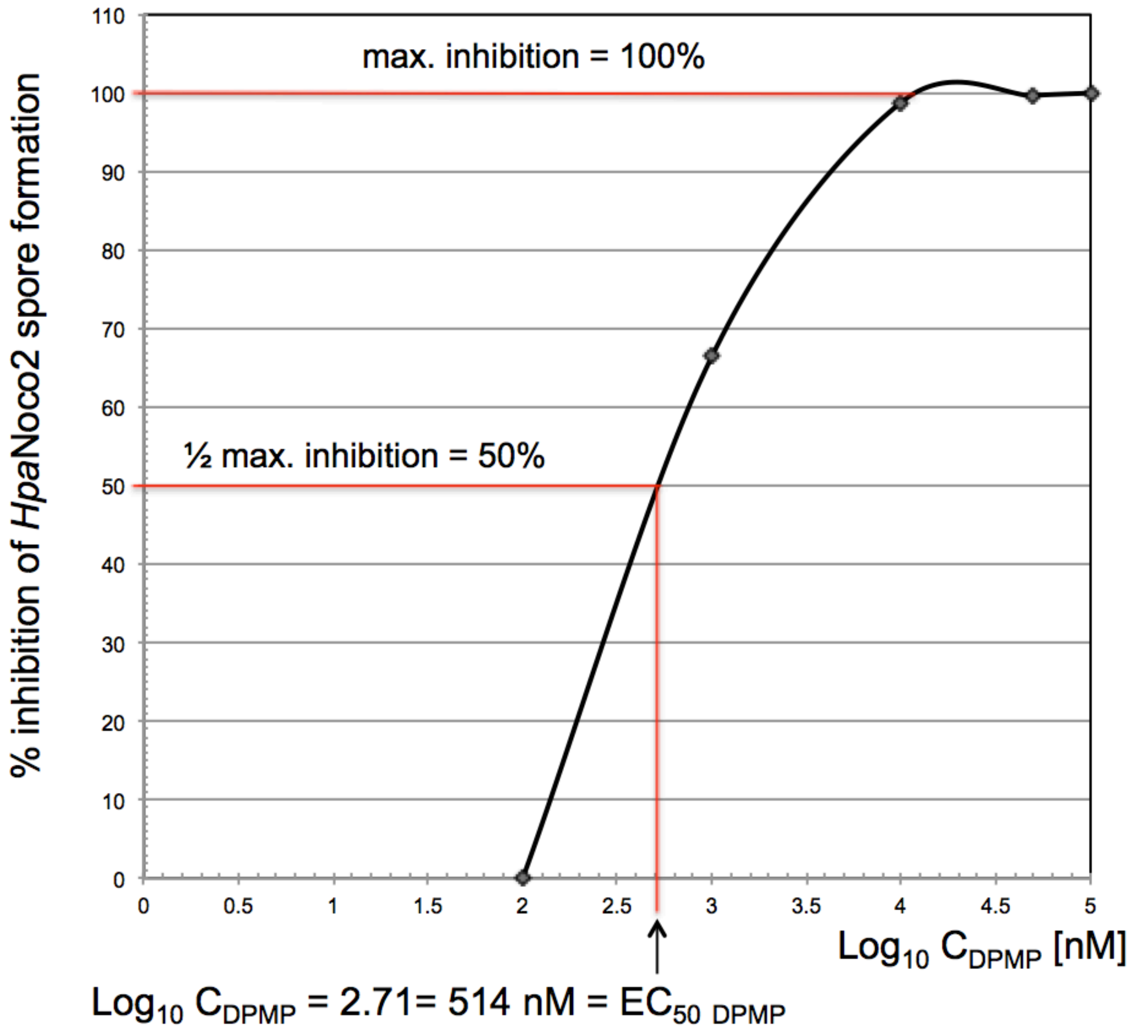

Supplementary Figure S2 online: Dose-dependency of DPMP mediated protection of Arabidopsis Col-0 against *Hpa*Noco2. Estimated EC_{50} value (concentration of a bioactive compound at which half-maximal biological activity is observed) based on the data shown in Figure 1 and new data for concentration of 0.1 µM DPMP. 100% inhibition = 0 spores; 0% inhibition = spore numbers after mock treatment.

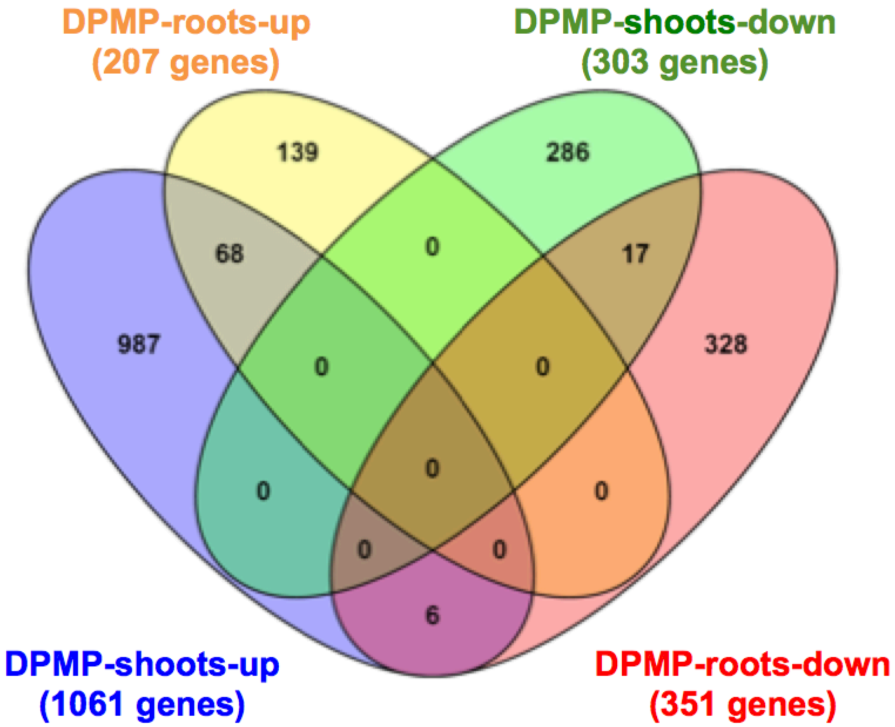

Supplementary Figure S3 online: Comparison of DPMP-triggered transcriptome changes in shoot and root. Venn diagram analysis highlights differences and similarities between the gene sets that were up-regulated from 3 μM DPMP in shoot and root.

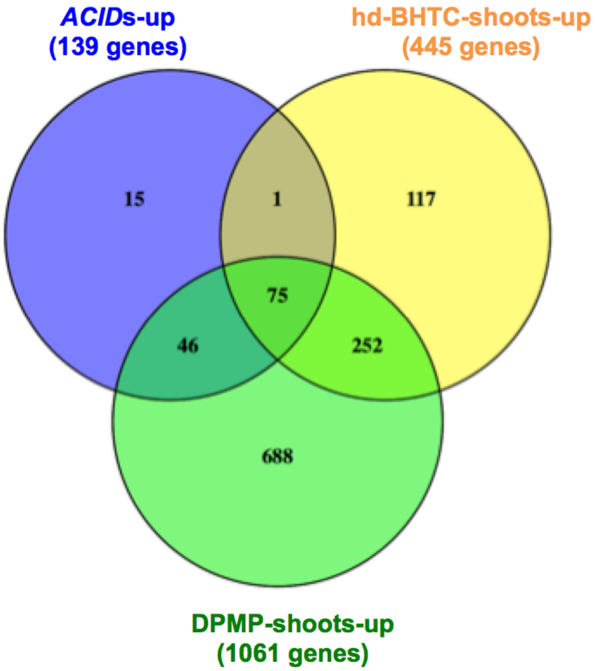

Supplementary Figure S4 online: Definition of a set of genes that are tighty accociated with chemically induced disease resistance. Comparison of up-regulated *ACID* genes[30] with sets of genes up-regulated in shoots in response to DPMP (this study) and a high dose of BHTC[32]. A set of 75 genes, termed "*SUPER ACID*s" is responsive to multiple synthetic elicitors.

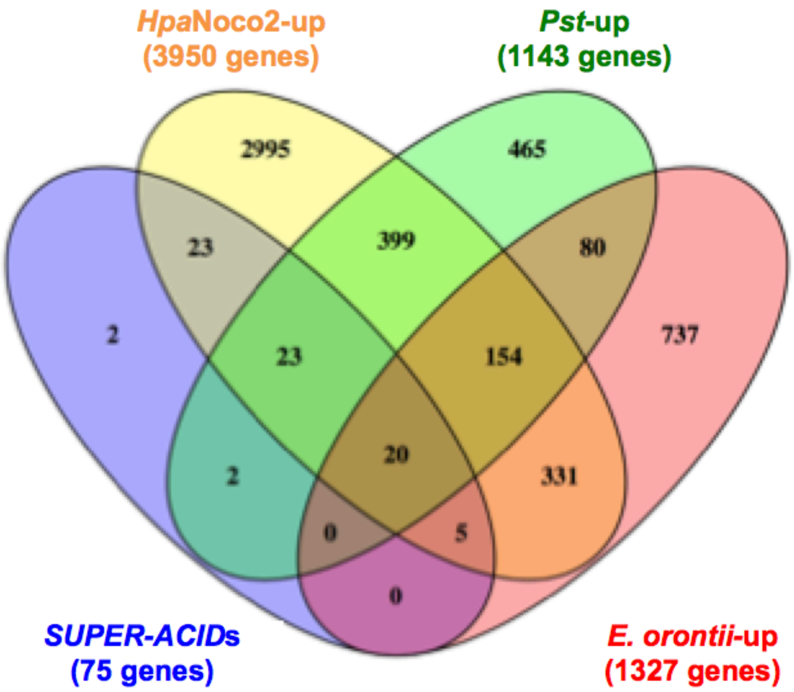

Supplementary Figure S5 online: Comparison of the set of *SUPER-ACIDs* with sets of genes up-regulated by *Erysiphae orontii, Hpa* and/or *Pst*.
